# The partial dehydrogenation of aluminium dihydrides†
†Electronic supplementary information (ESI) available. CCDC 1908297, 1916497, 1916498, 1908298 and 1919750. For ESI and crystallographic data in CIF or other electronic format see DOI: 10.1039/c9sc02750e


**DOI:** 10.1039/c9sc02750e

**Published:** 2019-08-13

**Authors:** Thomas N. Hooper, Samantha Lau, Wenyi Chen, Ryan K. Brown, Martí Garçon, Karen Luong, Nathan S. Barrow, Andrew S. Tatton, George A. Sackman, Christopher Richardson, Andrew J. P. White, Richard I. Cooper, Alison J. Edwards, Ian J. Casely, Mark R. Crimmin

**Affiliations:** a Department of Chemistry , Molecular Sciences Research Hub , Imperial College London , 80 Wood Lane, Shepherds Bush , London , W12 0BZ , UK . Email: m.crimmin@imperial.ac.uk; b Johnson Matthey Technology Centre , Blounts Court, Sonning Common , Reading , RG4 9NH , UK; c Department of Materials , University of Oxford , OX1 3PH , UK; d Australian Centre for Neutron Scattering , Australian Nuclear Science and Technology Organisation , Australia; e University of Wollongong , Wollongong , NSW 2522 , Australia; f Chemical Crystallography , Department of Chemistry , University of Oxford , 12 Mansfield Road , Oxford , OX1 3TA , UK

## Abstract

The reactions of a series of β-diketiminate stabilised aluminium dihydrides with ruthenium bis(phosphine), palladium bis(phosphine) and palladium cyclopentadienyl complexes is reported. In the case of ruthenium, alane coordination occurs with no evidence for hydrogen loss resulting in the formation of ruthenium complexes with a pseudo–octahedral geometry and *cis*-relation of phosphine ligands. These new ruthenium complexes have been characterised by multinuclear and variable temperature NMR spectroscopy, IR spectroscopy and single crystal X-ray diffraction. In the case of palladium, a series of structural snapshots of alane dehydrogenation have been isolated and crystallographically characterised. Variation of the palladium precursor and ligand on aluminium allows kinetic control over reactivity and isolation of intermetallic complexes that contain new Pd–Al and Pd–Pd interactions. These complexes differ by the ratio of H : Al (2 : 1, 1.5 : 1 and 1 : 1) with lower hydride content species forming with dihydrogen loss. A combination of X-ray and neutron diffraction studies have been used to interrogate the structures and provide confidence in the assignment of the number and position of hydride ligands. ^27^Al MAS NMR spectroscopy and calculations (DFT, QTAIM) have been used to gain an understanding of the dehydrogenation processes. The latter provide evidence for dehydrogenation being accompanied by metal–metal bond formation and an increased negative charge on Al due to the covalency of the new metal–metal bonds. To the best of our knowledge, we present the first structural information for intermediate species in alane dehydrogenation including a rare neutron diffraction study of a palladium–aluminium hydride complex. Furthermore, as part of these studies we have obtained the first SS ^27^Al NMR data on an aluminium(i) complex. Our findings are relevant to hydrogen storage, materials chemistry and catalysis.

## Abstract 2

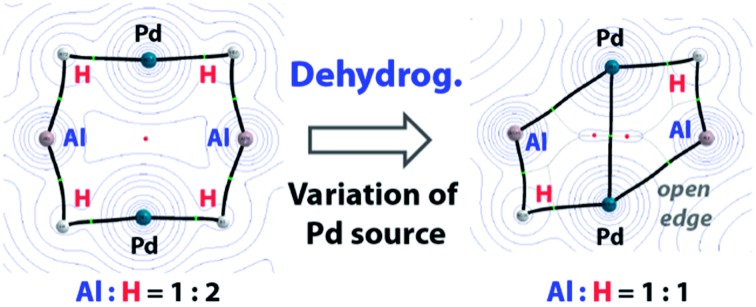
The reactions of a series of β-diketiminate stabilised aluminium dihydrides with ruthenium bis(phosphine), palladium bis(phosphine) and palladium cyclopentadienyl complexes is reported.

## Introduction

Metal aluminium hydrides (*e.g.* MAlH_4_, M = Li, Na, K) have been proposed as hydrogen storage materials.^1^ For example, LiAlH_4_ contains a high weight percentage of hydrogen and can undergo fast thermal dehydrogenation when doped with a transition metal, such as TiCl_3_, to form LiH and Al_(s)_.^2^,^3^ While the practical use of LiAlH_4_ for hydrogen storage remains a point of debate, the dehydrogenation step itself is of significant interest to materials and synthetic chemists alike. For example, very recently it has been shown that both the shape and size of aluminium nanocrystals formed during the dehydrogenation of AlH_3_·L can be controlled through careful selection of the ligand, L, on aluminium.^4^

The dehydrogenation of main group hydride compounds holds promise as a route to low-oxidation state intermediates. This approach has several advantages over current methods. The dehydrogenation of group 13 hydrides is potentially reversible through controlling the pressure of dihydrogen. It does not rely on the use of harsh reagents such as K or KC_8_ typical for metal dihalide reduction. Furthermore, many of the dihydride precursors are readily accessible on large scales through reactions of inexpensive and commercial hydrides such as LiAlH_4_ with suitable ligand precursors.

Although surprisingly little is known about the dehydrogenation of molecular aluminium hydrides,^5^–^7^ the microscopic reverse, the hydrogenation of low oxidation state aluminium compounds, is increasingly common. Dihydrogen undergoes oxidative addition to a handful of aluminium(i) compounds under mild conditions in solution.^8^–^10^ In a more extreme environment, Al_4_H_6_ has been generated by rapidly vaporising aluminium metal in the presence of dihydrogen. This cluster has been characterised as its anion in the gas-phase by photoelectron spectroscopy and shown to be just one of a broader series of molecules including Al_4_H_4_ and Al_*n*_H_*n*+2_ (*n* = 4–8).^11^,^12^

**Fig. 1:**
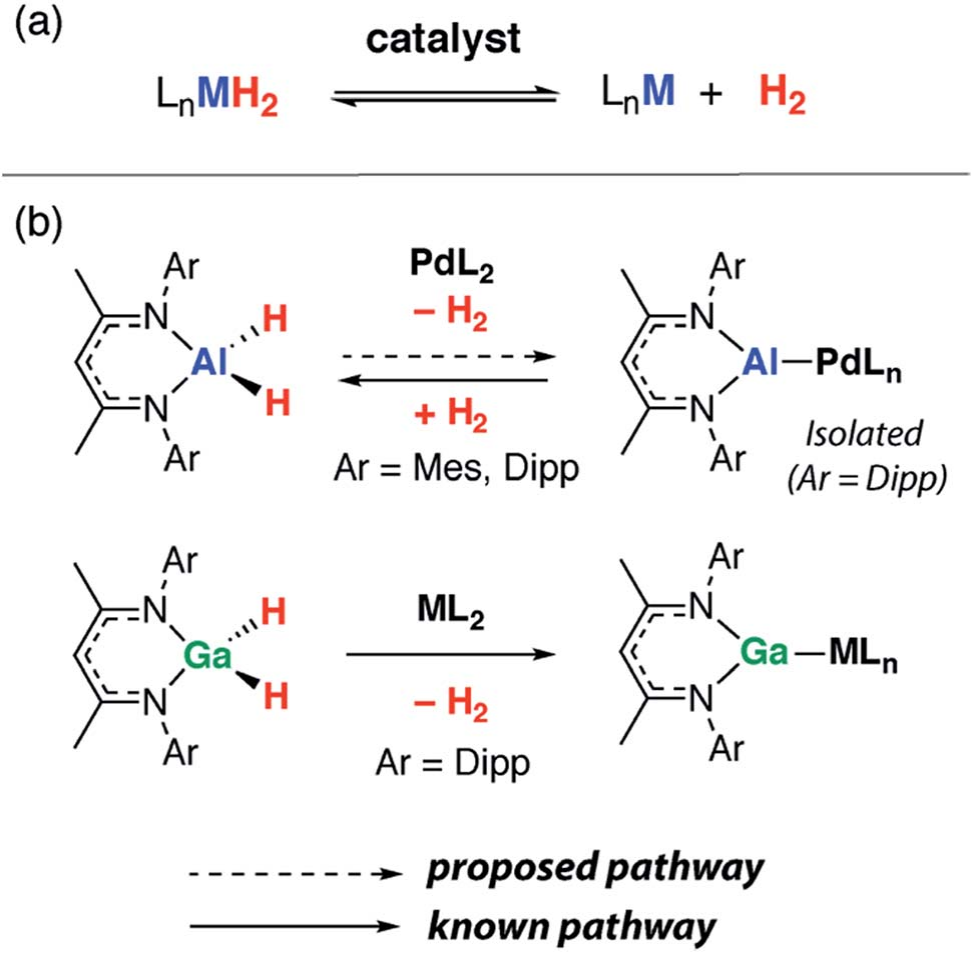
(a) Reversible dehydrogenation to form low-valent main group complexes. (b) Dehydrogenation of β-diketiminate Ga and Al dihydrides. (Mes = 2,4,6-trimethylphenyl, Dipp = 2,6-di-iso-propylphenyl).

As stated, precedent for the dehydrogenation step itself is limited. The formation of complexes containing {Al_2_H_4_} and {Al_6_H_6_} fragments following reduction of aluminium(iii) hydrides with a magnesium reagent has been reported.^13^,^14^ We have invoked the dehydrogenation of β-diketiminate stabilised aluminium(iii) dihydrides to form aluminium(i) ligands during the palladium catalysed transformation of C–H into C–Al bonds.^15^,^16^ The hypothesis is supported by the observation that analogous gallium(iii) dihydrides react with transition metal complexes by dehydrogenation (Fig. 1).^17^–^19^ In related studies, mesityl borane (MesBH_2_) has been shown to undergo reversible dehydrogenation on a ruthenium *bis*(phosphine) fragment to form a ruthenium borylene complex,^20^ while the aminoborane *i*-Pr_2_N–BH_2_ can be dehydrogenated with similar transition metal complexes provided a hydrogen acceptor (*tert*-butylethylene) is present.^21^

In this paper, we report a series of reactions that result in the partial dehydrogenation of the β-diketiminate stabilised aluminium dihydrides using transition metal complexes. While Ru—Al bimetallics involve expected three-dimensional geometries and show little sign of dihydrogen loss, related palladium compounds contain densely packed two-dimensional arrays of metal atoms bridged by hydride ligands. Although the complete dehydrogenation to form an aluminium(i) intermediate (or coordination complex thereof) is yet to be observed, we have isolated a series of complexes that contain different H : Al ratios (2 : 1 → 1 : 1) due to partial dehydrogenation. DFT calculations suggest that dehydrogenation is accompanied by the formation of covalent metal–metal bonds along with an associated increase in the negative charge on the Al centres; an effect that could be interpreted as lowering the formal oxidation state due to electron transfer to Al.

## Results and discussion

### Reactions of alanes with ruthenium complexes

**Scheme 1:**
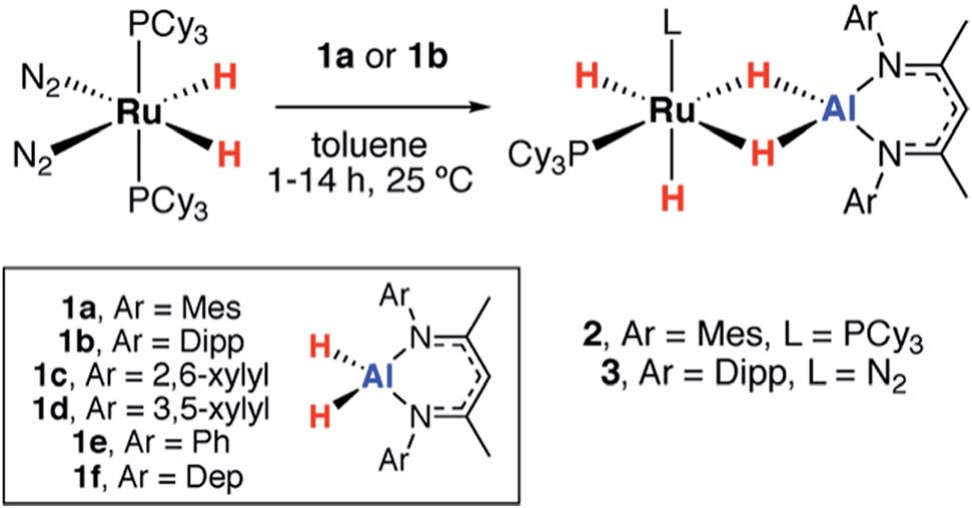
Reactions of ruthenium complexes with aluminium dihydrides. (Dep = 2,6-diethylphenyl).

The reactions of two aluminium dihydrides (**1a–b**) with a ruthenium *bis*(phosphine) complex were conducted. The choice of the metal fragment was dictated by the observation that closely related fragments have been shown to effect the reversible dehydrogenation of boranes.^20^ Addition of 1 equiv. of [Ru(H)_2_(N_2_)_2_(PCy_3_)_2_] to 1 equiv. of **1a** proceeded cleanly at 25 °C to generate the aluminium ruthenium heterobimetallic hydride complex **2** (Scheme 1).

**2** was characterised by a broad peak at *δ*_H_ = –11.77 ppm (fwhm = 1021 Hz) for all four hydrides in the ^1^H NMR spectrum at 293 K. Upon cooling to 273 K the hydride resonance decoalesced into two resonances at *δ*_H_ = –9.69 (fwhm = 77 Hz) and –13.49 (fwhm = 101 Hz) ppm assigned to the terminal and bridging hydrides respectively. At lower temperatures, a second fluxional process resolved at 213 K with four broad peaks observed at *δ*_H_ = –9.32 (fwhm = 84 Hz), –9.91 (fwhm = 77 Hz), –12.45 (fwhm = 217 Hz) and –13.86 (fwhm = 198 Hz) ppm in the ^1^H NMR spectrum for the four magnetically inequivalent hydrides. Long *T*_1(min)_ = 247 ms were measured for the hydride resonances at 313 K at 400 MHz, indicative of classical hydride behavior. At 293 K only one resonance was observed in the ^31^P{^1^H} NMR at *δ*_P_ = 69.9 ppm which upon cooling the reaction to 213 K decoalesced into two broad peaks observed at *δ*_P_ = 70.7 and 66.2 ppm. No ^2^*J*_P–H_ coupling could be resolved.

The data are consistent with at least one fluxional process operating which results in exchange of the hydride positions.^22^ The low temperature NMR spectroscopy data support the assignment of **2** in which the PCy_3_ ligands adopt a *cis*-arrangement. Although single crystals suitable for X-ray diffraction could not be obtained, the structure can confidently be assigned based on the multinuclear NMR data and comparison to the literature. Related σ-silane complexes have been reported by Sabo-Etienne and coworkers,^23^–^25^ while we recently disclosed a series of Ru–M (M = Al, Zn, Mg) complexes with similar coordination geometries.^26^

**Fig. 2:**
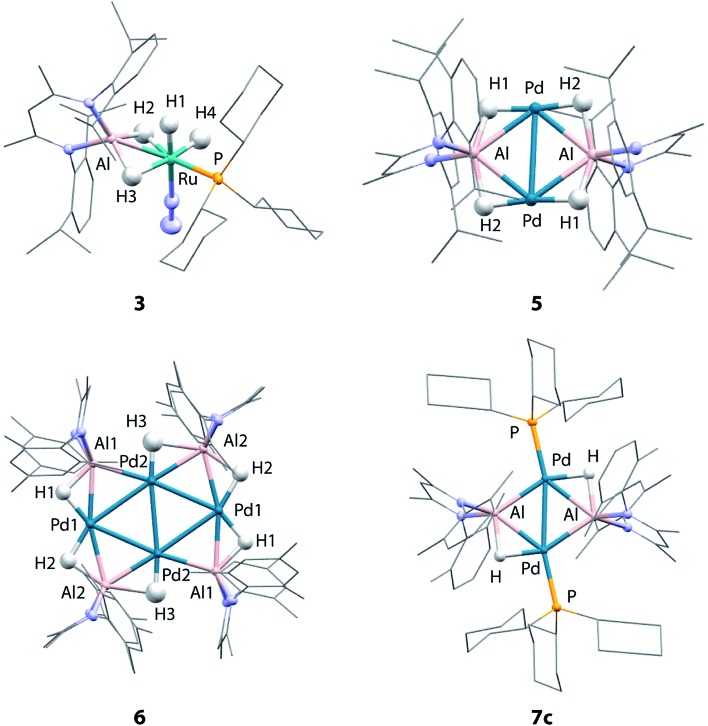
Crystal structures of **3**, **5**, **6** and **7c**.

A further reaction of 1 equiv. of [Ru(H)_2_(N_2_)_2_(PCy_3_)_2_] with 1 equiv. of **1b** proceeded cleanly at 25 °C to generate **3** (Scheme 1). This complex demonstrated three resonances for the hydride environments at 25 °C in the ^1^H NMR spectrum, *δ*_H_ = –9.05 (br s), –9.73 (d, ^2^*J*_P–H_ = 41.6 Hz), –16.11 (d, ^2^*J*_P–H_ = 15.8 Hz) ppm. At 233 K, the resonance at *δ*_H_ = –9.05 ppm decoalesced into two peaks at *δ*_H_ = –7.83 and –10.49 ppm. *T*_1_ measurements were taken across the 333–193 K range at 400 MHz and the four hydride signals exhibited a *T*_1(min)_ between 200–250 ms consistent with their assignment as classical hydrides.^27^,^28^ Monitoring the reaction between [Ru(H)_2_(N_2_)_2_(PCy_3_)_2_] and **1b** by ^31^P{^1^H} NMR spectroscopy revealed the formation of PCy_3_, *δ*_P_ = 9.9 ppm. The structure of **3** was ultimately confirmed by X-ray crystallography (Fig. 2). While the single crystal X-ray data are discussed in detail below, it is pertinent to note that, in contrast to **2**, **3** contains only a single coordinated PCy_3_ ligand. Dissociation of 1 equiv. of PCy_3_ is accompanied by coordination of N_2_ to Ru. The presence of a dinitrogen ligand in **3** was confirmed by IR spectroscopy with a strong absorbance at *ν*_N–N_ = 2135 cm^–1^ and the corresponding hydride stretches at *ν*_Ru–H_ = 1908 and 1872 cm^–1^.

### Reactions of alanes with palladium complexes

**Scheme 2:**
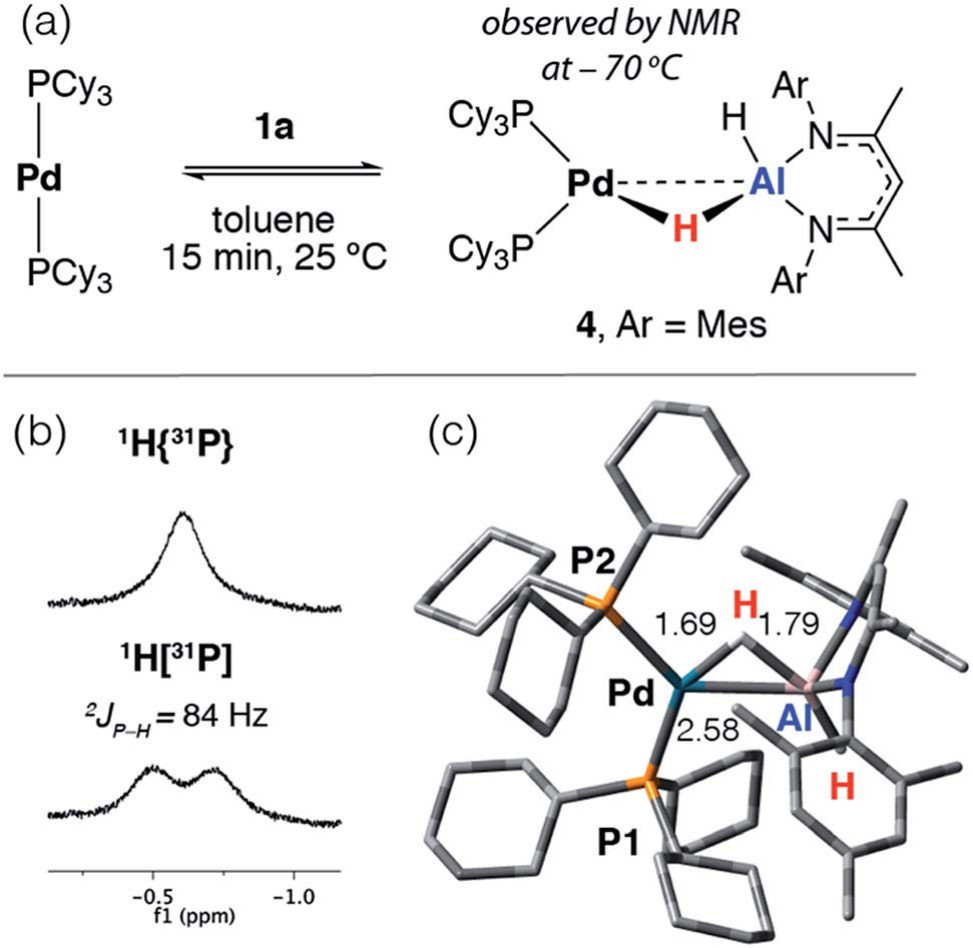
(a) Reversible reaction of **1a** with [Pd(PCy_3_)_2_]. (b) Hydride region of the ^1^H NMR spectrum at –70 °C in toluene-d_8_. (c) Calculated structure of **4** with selected bond lengths in Å.

The reaction of [Pd(PCy_3_)_2_] with **1a** in toluene-d_8_ at 25 °C quickly forms an equilibrium mixture due to reversible exchange of the phosphine and alane ligands (Scheme 2). The reaction was monitored using variable temperature NMR spectroscopy between 193 and 373 K. In the low temperature regime (below 203 K) two broad hydride resonances are observed at *δ* = –0.62 (d, ^2^*J*_P–H_ = 84 Hz) and +4.90 ppm attributed to bridging and terminal hydrides respectively in the σ-complex **4** (see ESI† for spectra). A ROESY experiment conducted at 193 K revealed chemical exchange between these two resonances, while their assignment as hydrides was unambiguously confirmed by repeating the experiment with the deuteride **1a-d_2_**. The large ^2^*J*_P–H_ coupling was confirmed by not only obtaining the ^1^H{^31^P} spectrum which shows selective decoupling but also the ^31^P NMR spectrum which shows a resonance at *δ* = 37.9 ppm (d, ^2^*J*_P–H_ = 84 Hz). In the high temperature regime (above 223 K), the diagnostic transition metal hydride resonance is no longer observed, suggesting a shift of the equilibrium back toward the starting materials. The ^2^*J*_P–H_ coupling constant of 84 Hz is significantly larger than the typical range of 0–20 Hz expected for Pd complexes with a *cis* relationship between the hydrogen and phosphorus atoms.^29^,^30^ Nevertheless, this *J* value is still smaller than the range of 150–200 Hz established for crystallographically characterised square-planar palladium complexes involving a *trans*^2^*J*_P–H_ coupling.^31^–^33^ An equivalent reaction using **1f** also gave a low intensity broad doublet resonance at *δ* = –0.61 ppm (^2^*J*_H–P_ ≈ 85 Hz) in the low temperature ^1^H NMR spectrum at 193 K which resolved to a broad singlet in the ^1^H{^31^P} NMR spectrum (see ESI†). Mixing [Pd(PCy_3_)_2_] with **1b** did not give an observable reaction by NMR spectroscopy, even at low temperatures, likely due to the increased steric bulk around the alane fragment.

The simplest explanation for these data is an associative process that forms the three-coordinate σ-alane complex **4**. We have isolated σ-alane complexes based on d^10^ Cu(i) fragments that are isoelectronic to [Pd(PCy_3_)_2_] and provided a detailed analysis of their electronic structure.^34^,^35^ In the current case, it appears that the σ-alane species is only stable at low temperature due to a weak binding event combined with an expected unfavourable reaction entropy. DFT calculations were used to interrogate the thermodynamics of ligand exchange and the formation of **4** was found to be modestly exergonic, consistent with the reversible process described above (see ESI† for details). The nature and magnitude of the ^2^*J*_P–H_ coupling in **4** are best described by considering the calculated geometry in which the bond angles approach those associated with *trans* (P1–Pd–H = 150°) and *cis* (P2–Pd–H = 87°) geometries. Due to line broadening effects it appears that only the larger of ^2^*J*_P–H_ couplings, associated with the *trans*-like relation between the hydride atom and a single PCy_3_ ligand, is resolved in the low temperature NMR data.

Related reversible processes involving the addition of silanes and germanes to [Pd_2_(dcpe)_2_] (dcpe = 1,2-*bis*(dicyclohexylphosphino)ethane) have been reported to form [Pd(dcpe)(H)(ER_3_)] (E = Si, Ge).^36^,^37^ In these instances, σ-silane or σ-germane complexes are invoked only as unstable intermediates in reversible redox processes that involve the oxidative addition and reductive elimination of the E–H bond to the palladium centre. Despite the precedent for **4**, at this time we cannot unambiguously rule out its assignment as a linear two coordinate complex formed from phosphine dissociation from Pd. Indeed, based on the calculated thermodynamics of phosphine dissociation (see ESI†) both the latter species and **4** may be formed as part of an equilibrium mixture.

Further evidence for the interaction of Al–H bonds with the Pd centre derives from H/D exchange reactions. The Al–H bonds of **1a** and **1c** were observed to undergo H/D scrambling when in the presence of [Pd(PCy_3_)_2_] and C_6_D_6_ under ambient conditions. Storage of a C_6_D_6_ solution of **1c** with a catalytic amount (5 mol%) of [Pd(PCy_3_)_2_] resulted in loss of the broad Al–H resonance at *δ* = 4.59 ppm in the ^1^H NMR spectrum and a corresponding increase (compared to a ferrocene standard in a capillary) of the residual solvent peak for C_6_D_5_H after 5 days. Removal of the solvent and dissolving in C_6_H_12_ (to prevent further reaction) allowed collection of the ^2^H NMR spectrum which showed the largest peak to be a broad resonance at *δ* = 4.39 ppm, corresponding with the Al–D resonance of **1c-d_2_**.

**Scheme 3:**
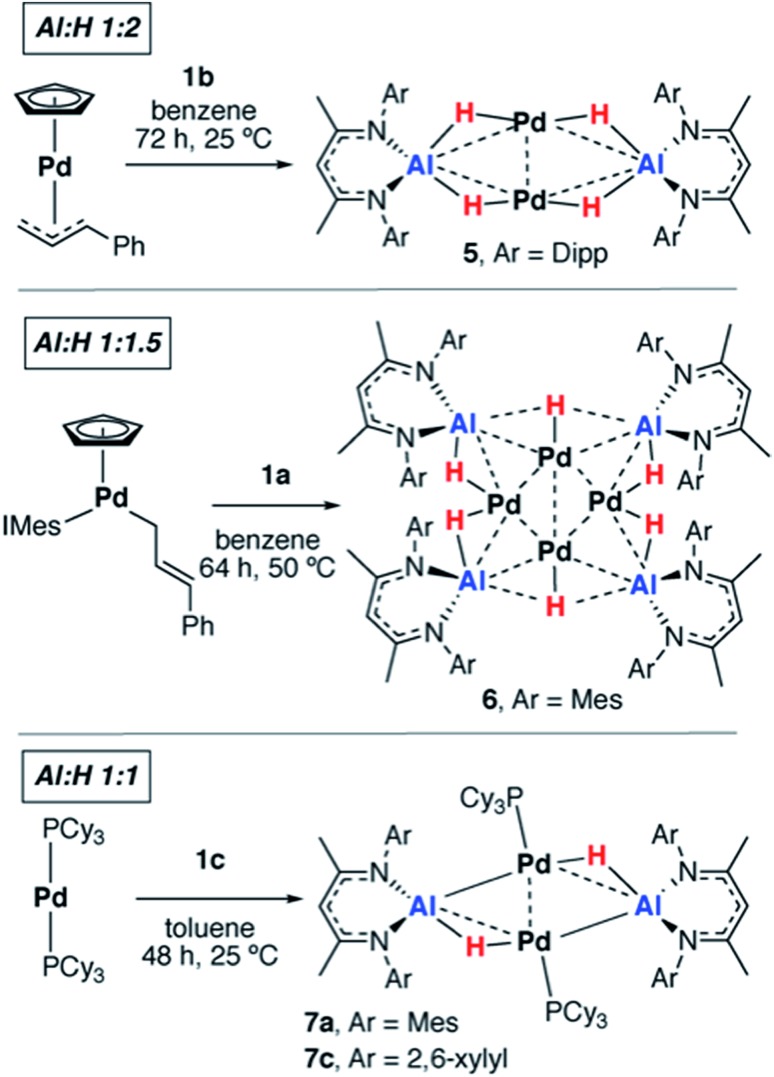
Non-reversible reactions of **1a–c** with palladium precursors.

We have previously reported that, upon standing at 25 °C solutions of [Pd(PCy_3_)_2_] and **1a** gradually yield red crystals of **7a** complex (Scheme 3) as a result of the non-reversible dimerization and dehydrogenation of **4**.^15^**7a** is insoluble in hydrocarbon solution and it appears the crystallisation may play a role in driving the equilibria towards this product. Modification of the palladium and alane precursors allowed identification of similar non-reversible reactivity and isolation of a series of remarkable intermetallic complexes all involving Pd–H–Al interactions but differing in the ratio of H : Al due to partial dehydrogenation. Hence, the reaction between **1b** and [Pd(η^5^-C_5_H_5_)(η^3^-C_3_H_4_Ph)] yielded the tetrametallic complex **5** with a 2 : 1 ratio of H : Al, while addition of **1a** to [Pd(η^5^-C_5_H_5_)(η^1^-C_3_H_4_Ph)(IMes)] (IMes = 1,3-*bis*(2,4,6-trimethylphenyl)imidazol-2-ylidene) led to the isolation of the octametallic cluster **6** with a 1.5 : 1 ratio of H : Al (Scheme 3). Dihydrogen was observed as a low intensity resonance at *δ* = 4.47 ppm in the ^1^H NMR spectra of the reaction mixtures of **6** and **7c** which disappeared upon degassing of the sample.

A systematic investigation of the alane (**1a–f**, Scheme 1) in the reactions involving [Pd(PCy_3_)_2_] showed that the dehydrogenation to form complexes with a 1 : 1 ratio of H : Al was highly dependent on the steric demands of the ligand (Scheme 3). **1c** reacted with [Pd(PCy_3_]_2_ to give red crystals of **7c**, an exact analogue of the mesityl substituted derivative **7a**. **1f** reacted with [Pd(PCy_3_)_2_] at 60 °C (160 h) to give an orange solution with a ^31^P{^1^H} NMR spectrum displaying broad peaks at *δ* = 38.9 and 9.8 ppm for [Pd(PCy_3_)_2_] and free PCy_3_ respectively and a new resonance at *δ* = 40.1 ppm. Dihydrogen was observed in the ^1^H NMR spectrum which along with free PCy_3_ suggests a product similar to **7c** but we were unable to isolate a pure solid or single-crystals from this reaction, preventing characterisation of this product. Further reactions of **1a–b** with [Pd(P^t^Bu_3_)_2_] or [Pd(PPh_3_)_4_] led to the formation of non-crystalline insoluble material and complex reaction mixtures respectively. There was no apparent reaction between **1b** and [Pd(PCy_3_)_2_] after heating to 80 °C for 120 h. Addition of **1d** and **1e** with [Pd(PCy_3_)_2_] led to the formation of complex mixtures. Although the complete product distribution could not be determined, a few single crystals obtained from the reaction of **1e** with [Pd(PCy_3_)_2_] allowed identification of a decomposition pathway involving hydride transfer from the metals to the electrophilic C=N position of the backbone of the ligand system (see ESI†). It appears that *ortho*-substituents on the aryl groups of the ligand are a necessary requirement to block the electrophilic site and prevent this mode of decomposition.

The new Pd–Al species are insoluble in common laboratory solvents (benzene, toluene, hexane, THF, Et_2_O) preventing acquisition of complete NMR data. Nevertheless, the partial solubility of **5** allowed the hydride resonances to be located at *δ* = –2.17 ppm (fwhm = 50 Hz) in C_6_D_6_ at 298 K provided the ^1^H{^27^Al} spectrum was obtained. All new species possess the expected complex set of IR stretches below 1600 cm^–1^ and, consistent with previous observations,^7^ the bridging nature of the Al–H–Pd interaction shifts the hydride vibrations into the fingerprint region of the spectra.

### Solid-state structures

Although the low solubility of **5**, **6** and **7c** in standard solvents precluded solution characterisation, these complexes have been characterised by single crystal X-ray diffraction (Fig. 2). In all four structures, the hydride ligands were located in the difference map and the positions refined freely. Where possible their presence has been confirmed by other spectroscopic or chemical methods. In the Pd/Al complexes **5**, **6** and **7c** the only hydride ligands were found to bridge Pd–Al edges and to lie in the plane of the metal centres. Crystallographically characterised structures of Pd dimers and cluster complexes containing Pd···H···Pd bridging interactions have Pd–Pd distances similar to those found in **5**, **6** and **7c**,^38^–^40^ but we do not find evidence of edge-bridging or face-capping Pd···H···Pd hydrides in the complexes reported herein. DFT studies have been carried out to confirm the presence and position of the crystallographically assigned hydride ligands (*vide infra*) with structural optimisation showing good correlation between the experimental and calculated structures.^41^

The structure of **3** confirms the loss of one PCy_3_ ligand from the starting material as indicated by the solution studies. The Ru(ii) centre is in a distorted octahedral environment with one PCy_3_ ligand, one terminally bound dinitrogen, and the alane bound through both hydrides. Two terminal hydrides complete the coordination sphere. The PCy_3_ ligand is *trans* to one of the Al bound hydrides. The Ru–Al distance of 2.3037(9) Å is within the sum of the single covalent bond radii^42^,^43^ despite the two bridging hydrides. The majority of previously reported Ru–Al complexes contain more than two metal centres and the Ru–Al distance in **3** is amongst the shortest reported.^26^,^44^–^47^ The examples of complexes with shorter Ru–Al distances result from reaction of low-valent [Cp*Al]_4_ with Ru precursors.^46^,^47^

**5** contains a rhomboidal core of {Pd_2_Al_2_} with Pd and Al in alternate positions and hydride ligands bridging each edge of the square. The Pd–Pd distance (3.0655(3) Å) is shorter than the Al–Al (3.934 Å) distance. The molecule contains an inversion centre with an asymmetric unit of half the {Pd_2_Al_2_H_4_} complex and the {Pd_2_Al_2_H_4_} unit is found to be almost planar. The plane created by Al and the two nitrogen atoms in the supporting ligand is almost perpendicular (89.8°) to the {Pd_2_Al_2_} plane, also indicating that the hydride ligands lie in the {Pd_2_Al_2_} plane. The alane is the sole ligand for the Pd–Pd unit and is bound slightly asymmetrically with Pd–Al distances of 2.4663(6) and 2.5203(6) Å. The Pd–Pd and Pd–Al distances are slightly longer than those found in a previously reported analogue **7a** (Pd–Pd 2.8717(4) Å; Pd–Al 2.4308(7) and 2.4538(8) Å) which shares the same metal connectivity.^15^

The structure of **6** features an octametallic {Pd_4_Al_4_} core, double that of **5**, **7a** and **7c**. The metal centres lie in a plane around an inversion centre with the Pd centres forming a diamond shape and Al centres bridging each edge of this diamond. The structure is found to contain 6 hydride ligands (H : Al = 1.5 : 1) which also lie in the plane of the metals. Al2 is still nominally bound to 2 hydrides while Al1 only bonds to one, although the uncertainty in the position of H3 means it is possible H3 is shared between Al1 and Al2. Pd1 forms two hydride bridges while Pd2 has only one. The Pd–Pd distances fall in a 0.1 Å range with the Pd2–Pd2′ distance the shortest (2.8883(5) Å) and the Pd–Al distances also fall in a 0.1 Å range of one another. The planes created by Al and the two nitrogen atoms in the supporting ligands are again almost perpendicular (89.3 and 87.6°) to the plane of the metal centres. There does not appear to be literature precedent for this type of planar Pd containing structure. Complexes with four or more Pd centres in this diamond configuration are generally not planar, those close to planarity are forced so by being sandwiched between planar aromatic ligands.^48^–^52^ A few high nuclearity clusters supported by main group ligands are known, including triangular (*tris*)platinum complexes and planar (*tetrakis*)palladium complexes. In the latter a single Pd centre is connected to three Pd and Si or Ge atoms.^53^–^55^ To the best of our knowledge, there are no reported structurally characterised examples of Pd_4_Al_4_ species or of Al complexes with any transition metal with the configuration observed in **6**. Furthermore, complexes **5** and **6** represent rare examples of palladium clusters free from addition phosphine ligands on the group 10 metal.

Two crystal forms of **7c** were identified by diffraction experiments. Single crystal X-ray diffraction of red crystals of form 1 (triclinic, P-1, *V* = 1597 Å^3^) obtained from benzene solution revealed that the metallic core of **7c** is virtually identical to that of **7a** with the same planar {Pd_2_Al_2_H_2_} motif and H : Al ratio of 1 : 1.^15^ The change in the supporting ligand for Al from containing mesityl groups in **7a** to 2,6-xylyl groups in **7c** appears to have little effect. The Pd–Pd (**7c**: 2.8760(3) Å; **7a**: 2.8717(4) Å) and Pd–Al (**7c**: 2.4360(5) and 2.4504(5) Å; **7a**: 2.4308(7) and 2.4538(8) Å) distances are similar in both cases leading to topologically identical structures. Form 2 (triclinic, P-1, *V* = 3717 Å^3^) formed as large red blocks from a C_6_D_6_ solution and was subject to single crystal neutron diffraction. This experiment revealed that H/D isotope exchange between the hydrides and the solvent had occurred during the crystallisation (see above) providing an isotopically enriched sample of **7c-d_2_**. Neutron diffraction of **7c-d_2_** confirmed the position and number of the deuteride ligands and hence the corresponding hydrides in **7c**. Two molecules are present in the asymmetric unit of this form. The Pd–D bond lengths of 1.80(2) Å is similar to the Al–D bond lengths of 1.77(3) Å and are consistent with the proposed bridging interaction. This rare example of a neutron structure of a palladium hydride species validates a general structural motif that has been invoked numerous times for related Pd and Pt complexes with group 14 hydrides (Si and Ge) (Table 1).^56^–^61^

**Table 1:** Selected bond lengths (Å) in **3**, **5**, **6** and **7c**

Compound	M–Al	M–M	M–P	M–D	Al–D
**3**	2.3037(9)	—	2.3149(8)	—	—
**5**	2.4663(6)	3.0655(3)	—	—	—
	2.5203(6)				
**6**	2.4137(10)	2.9197(4)	—	—	—
	2.5089(10)	2.9720(4)			
	2.4320(10)	2.8883(5)			
	2.4222(11)				
**7c** (X-ray-form 1)	2.4360(5)	2.8760(3)	2.3397(5)	—	—
	2.4504(5)				
**7c** (X-ray-form 2)	2.4332 (9)	2.9329(5)	2.3744(8)	—	—
	2.4496(9)				
	2.4384(9)	2.8879(4)	2.3358(8)		
	2.4419(9)				
**7c** (Neutron-form 2)	2.43(3)	2.90(3)	2.31(2)	1.77(2)	1.81(3)
	2.43(3)				
	2.43(3)	2.86(3)	2.39(2)	1.80(2)	1.77(3)
	2.42(3)				

### Solid-state ^27^Al NMR data

^27^Al NMR spectroscopy was used as a means to gain insight into the Al environment upon coordination and dehydrogenation. In our experience, acquisition of satisfactory solution ^27^Al NMR data on a broad range of aluminium complexes with or without metal coordination is challenging because of the line-broadening effects inherent with the quadrupolar *I* = 5/2 ^27^Al nucleus. Solid state (SS) NMR spectroscopy offers an alternative approach to study broad signals, by accessing higher-power pulses enabling a greater excitation bandwidth, and by employing magic angle spinning (MAS) to reduce the effects of chemical shift anisotropy and quadrupolar interactions. In combination with computational methods, this provides a powerful approach.^62^–^65^

In order to validate the approach, a series of reference samples were investigated by ^27^Al MAS NMR spectroscopy. Considering the aluminium fragments in isolation, two extremes for the extent of dehydrogenation are clearly represented by the aluminium dihydrides (**1a–f**) and the corresponding monomeric aluminium(i) complex **8**, originally reported by Roesky and co-workers.^66^ Further points of comparison, the aluminium dichloride complexes, **9a–b** were considered.^67^ Isotropic chemical shift values obtained from SS ^27^Al NMR spectroscopy were confirmed by comparison to the available solution state data and showed good agreement, validating the approach (Table 2).

**Table 2:** Isotropic chemical shift, *δ*_Al_ and *C*_Q_ values for **1a–b** and **8–9**

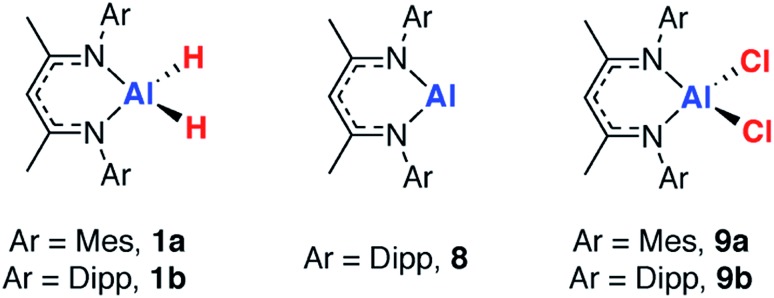			
Compound	Solution NMR *δ*_Al_ (ppm)	SS NMR isotropic *δ*_Al_ (ppm)^*a*^	SS NMR C_Q_ (MHz)^*b*^
**1a**	—	100	14.2
**1b**	130^70^	130	15.0
**8**	590 ± 40^68^	120	**23.0**
**9a**	—	95	4.8
**9b**	100^70^	98	3.9

^*a*^Experimentally determined values.

^*b*^Obtained from DFT simulations.

**Fig. 3:**
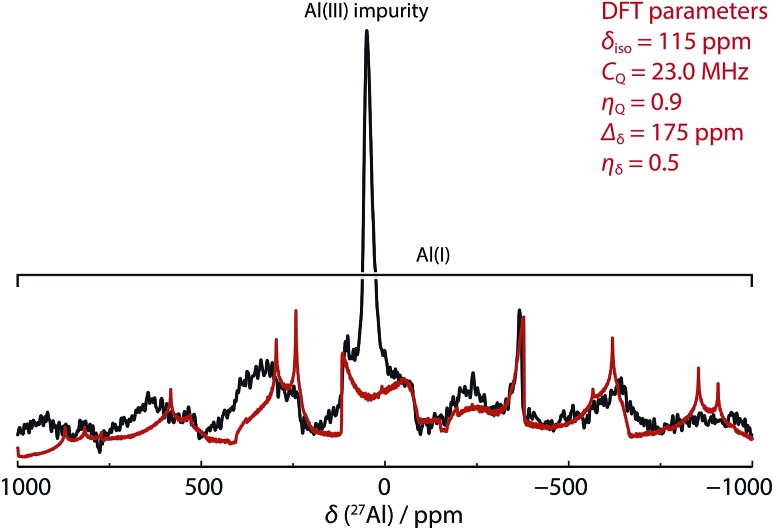
SS ^27^Al MAS NMR spectrum of **8** (black line, 52 464 scans with 1 s relaxation delay) overlaid with simulated lineshape of just the central transition from DFT parameters (red line).

In our hands, ^27^Al MAS NMR spectroscopy of **8** showed a remarkably broad spectrum with isotropic shift *δ*_Al_ = 120 ppm and a series of features seen out to +1000 and –1000 ppm. ^27^Al NMR data for a series of aluminium(i) compounds bearing cyclopentadienyl ligands have been calculated to range from *δ*_Al_ = +850 to –170 ppm.^69^ While in solution it has been suggested that **8** possesses a ^27^Al NMR chemical shift of *δ*_Al_ = 590 ± 40 ppm (fwhm = 30 000 Hz),^68^ it appears that this only represents part of the spectral features apparent in the solid-state. To support the new assignment DFT calculations were performed, which agreed very well with experiment, and gave NMR parameters of *δ*_Al_ = 115 ppm with *C*_Q_ = 23.0 MHz and CSA = 175 ppm (Fig. 3 – red line). At 9.4 T and 30 kHz MAS, this results in a very wide central transition peak, which is strong evidence for the highly asymmetric Al(i) environment. The same spectral features were obtained from three independently synthesised samples of **8**. In all samples, a peak consistent with a major AlO_4_ impurity were observed. Upon exposure of samples of **8** to air a visible change in colour from red to colourless was observed and ^27^Al MAS NMR confirmed the loss of the resonances associated with **8**. For comparison, *δ*_Al_ and *C*_Q_ values for **1a–b**, and **9a–b** are listed alongside those of **8** in Table 2. This is an exceptionally rare example of a successful ^27^Al MAS NMR spectroscopy experiment on an aluminium centre in the +1 oxidation state.

Comparison of the data reveals that absolute values of the isotropic chemical shift are not a good indicator of formal oxidation state. Schnöckel and co-workers have already commented that the ligand environment and coordination number at aluminium has a large impact on chemical shift values.^69^ Further, Arnold and co-workers have compared the electronic structure of **1**, **8** and **9** using a combination of polarised aluminium K-edge absorption near edge structure (XANES) spectroscopy and first-principles calculations.^70^ They concluded that, despite the difference in formal oxidation state of aluminium, the charge distributions about aluminium are similar throughout the series. The *C*_Q_ values are an indicator of asymmetry in the electric field gradient across the molecule and show a rational trend. Complex **8** has the highest *C*_Q_ value (23.9 MHz DFT calculations), indicative of the two-coordinate environment at aluminium. As the crystal field environment about Al becomes more symmetric with introduction of hydride, alkyl and chloride ligands values of *C*_Q_ decrease. The *C*_Q_ values are less sensitive than *δ*_Al_ to changes of the steric profile of the β-diketiminate ligand (Table 2).

^27^Al MAS NMR spectra were recorded on a series of transition metal alane complexes with different H : Al ratios. These included Ru and Pd complexes reported herein along with some previously isolated Rh complexes from our group.^71^ As expected from the reference samples, there is no clear correlation of the *δ*_Al_(*iso*) or *C*_Q_ values with the extent of dehydrogenation (see ESI, Table S6.1†), suggesting that an alternative approach is required to interrogate the dehydrogenation step.

### DFT and QTAIM calculations

In contrast to the ^27^Al NMR experiments, DFT calculations provided a good indicator for the extent of on-metal dehydrogenation. Both NBO and QTAIM calculations are consistent with dehydrogenation being a process in which dihydrogen production is accompanied by metal–metal bond formation and transfer of electron-density to the Al centres.

**Fig. 4:**
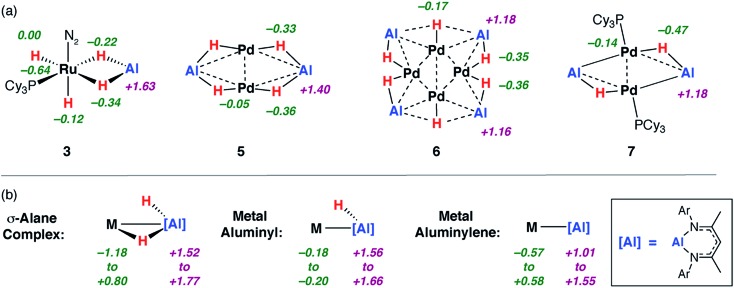
Calculated NPA charges for (a) **3**, **5–7c** alongside (b) a series of metal σ-alane, aluminyl and aluminylene complexes.

Analysis of the NPA charges from NBO calculations on geometry optimised structures were performed on both the aforementioned reference compounds and a comprehensive series of aluminium containing heterometallic complexes reported by our group and others (Fig. 4), including σ-alane complexes,^34^,^72^ metal aluminyls bearing an X-type aluminium ligand,^71^ and metal aluminylenes involving an l-type aluminium ligand.^15^ There is a trend in the charge on aluminium as the structures proceed from σ-alane to aluminyl to aluminylene ligands. As the ratio of H : Al decreases across the series **3** > **5** > **6** > **7c** the charges on the aluminium centres decrease in the same order +1.6 > +1.4 > +1.2 = +1.2. These charges parallel the range established for the reference complex categories in Fig. 4 and the calculated NPA charges on the genuine Al(iii) and Al(i) complexes **1b** and **8** of +1.5 and +0.8 respectively.

**Fig. 5:**
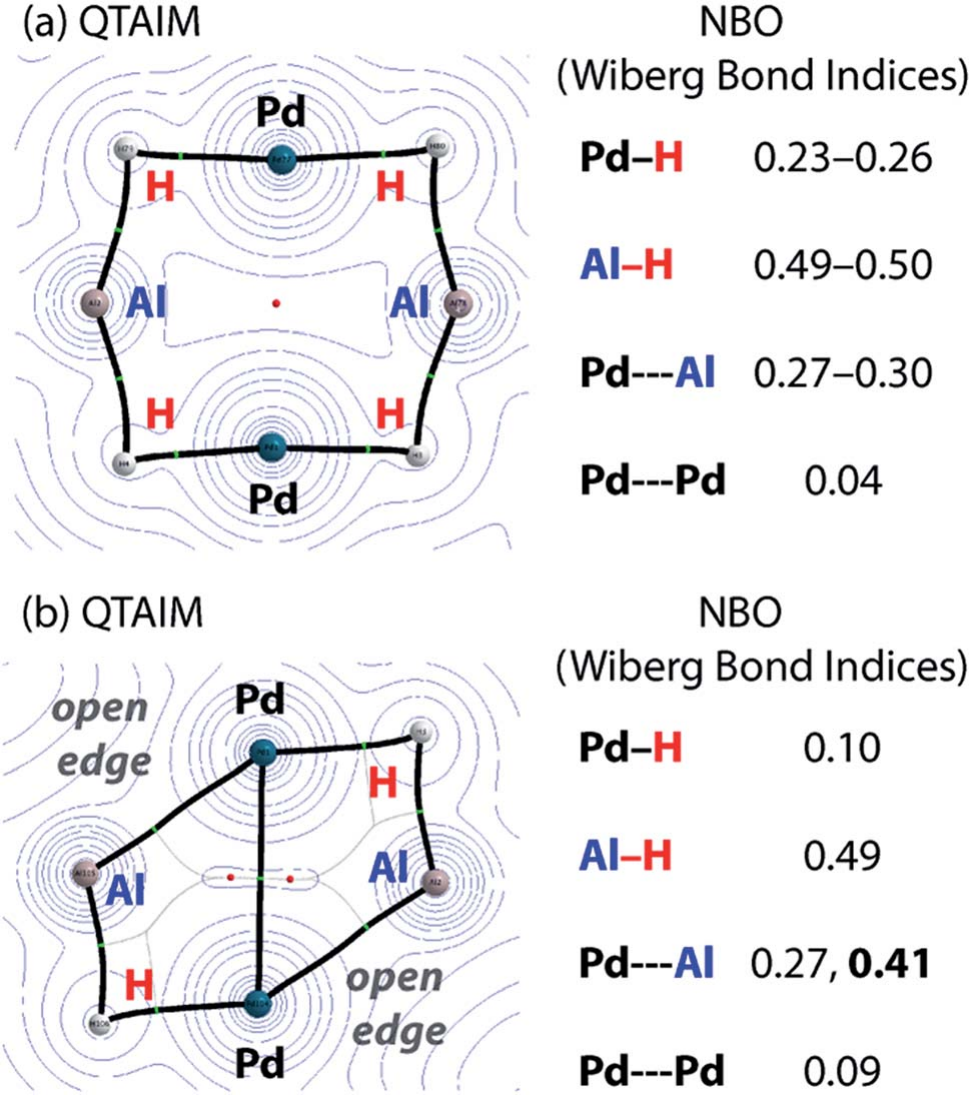
Comparison of QTAIM molecular graphs for (a) **5** and (b) **7c**.

Charge transfer to Al appears to be a result of the formation of covalent metal–metal bonds. The nature of the metal–metal interactions can be considered more deeply by comparing results from DFT and QTAIM calculations. While the Pd–Pd and Pd–Al distances in **5** and **7c** vary little regardless of structure, calculations provide more insight. Comparison of the QTAIM molecular graphs (Fig. 5) shows that where hydride ligands are present the bonding is dominated by 3c,2e^–^ interactions involving Pd–H–Al bridges. Critical paths are found between Al and H atoms and Pd and H atoms but not between Pd and Al. NBO calculations provide a similar picture, characterising a high degree of covalency and a bridging interaction that is dominated by metal–hydride bonding. Pd–Al bonds form as hydride ligands are removed. Hence, in **7c** QTAIM data return bond paths between Pd and Al for the edges without hydride ligands but not those with. NBO calculations support a much larger degree of Pd–Al covalent bonding along these open edges and a bonding situation reminiscent of that found in metal aluminyl complexes.^71^ While the nature of the Pd–Pd bonding in these complexes is less clear, based on NBO calculations and the low covalent character in both **5** and **7c**, it is likely dominated by closed shell interactions.

The data imply that on-metal dehydrogenation is accompanied by metal–metal bond formation. Clusters with lower hydride content form metal–metal bonds where bridging 3c,2e^–^ M–H–M interactions are not possible. Due to the large covalent character of the Pd–Al metal bonds dehydrogenation occurs with charge accumulation on aluminium. While this finding could be interpreted in terms of a decrease of the formal oxidation state of aluminium due to dehydrogenation, care must be taken with the assignment of oxidation states to the Al and transition metal centres in these molecules. Values of formal oxidation state are often of little meaning in molecules with a high covalent character to the bonding. Nevertheless, the calculations show a clear change in the electrostatic component to the bonding as hydride ligands are lost and metal–metal interactions are formed.

## Conclusions

In summary, we report a series of reactions of structurally related aluminium dihydrides with Ru and Pd transition metal fragments. While the resulting ruthenium–aluminium heterobimetallics are 3-dimensional coordination compounds involving ligation of the aluminium dihydride to Ru, the isolated palladium–aluminium complexes all incorporate a 2-dimensional array of metal atoms supported by auxiliary ligands. These latter intermetallic complexes contain Pd–Al and Pd–Pd interactions and differ in terms of the H : Al ratio. Those with lower hydride content form with liberation of dihydrogen. Despite observation of palladium–aluminium intermetallics with 2 : 1, 1.5 : 1 and 1 : 1 ratios of H : Al, the complete dehydrogenation of the molecular aluminium dihydrides is yet to be observed. Spectroscopic methods and calculations were used to gain insight into the on-palladium dehydrogenation process. ^27^Al MAS NMR spectroscopy parameters did not correlate with the level of dehydrogenation but did allow the unambiguous characterisation of an Al(i) complex by ^27^Al MAS NMR. DFT and QTAIM calculations showed that the extent of alane dehydrogenation was accompanied by a decrease in the charge on the aluminium centres: along with Pd–Al bond formation in molecular regions in which hydride ligands had been removed.

Despite increased interest in aluminium hydrides as hydrogen storage materials little is known about the intermediates in the transition metal mediated dehydrogenation process. This study provides some of the first structural snapshots of on-metal dehydrogenation of alanes by using sterically demanding ligands to access kinetic products of hydrogen loss. The data we report begins to inform not only the mechanism of interconversion of aluminium(iii) hydride precursors and aluminium(0) materials in hydrogen storage applications, but also recently reported catalytic methods that involve dehydrocoupling of molecular aluminium(iii) hydrides with organic substrates.

## Conflicts of interest

The authors declare no conflicts of interest.

## Supplementary Material

Supplementary information

Supplementary information

Crystal structure data
