# Supplementary material for: The partial dehydrogenation of aluminium dihydrides
Source: Chem Sci. 2019 Aug 13;10(35):8083–93. doi: 10.1039/c9sc02750e (PMC6855256; doi:10.1039/c9sc02750e)
Supplement: Supplementary file 2 [file SC-010-C9SC02750E-s002.pdf]

Electronic Supplementary Material (ESI) for Chemical Science.
This journal is © The Royal Society of Chemistry 2019

Electronic Supplementary Information for:

The Partial Dehydrogenation of Aluminium Dihydrides

Thomas N. Hooper,[a] Samantha Lau,[a] Wenyi Chen,[a] Ryan K. Brown,[a] Martí Garçon,[a] Karen Luong,[a] Nathan S. Barrow,[b] Andrew S. Tatton,[c] George A. Sackman,[d,f] Christopher Richardson,[e] Andrew J. P. White,[a] Richard I. Cooper,[f] Alison J. Edwards,[d] Ian J. Casely[b] and Mark R. Crimmin[a,]*

[a]*Department of Chemistry, Molecular Sciences Research Hub, Imperial College London, 80 Wood Lane, Shepherds Bush, London, W12 0BZ, UK.*

[b] *Johnson Matthey Technology Centre, Blounts Court, Sonning Common, Reading, RG4 9NH, UK.*

[c] *Department of Materials, University of Oxford, OX1 3PH, UK.†*

[d]*Australian Centre for Neutron Scattering, Australian Nuclear Science and Technology Organisation.*

[e]*University of Wollongong, Wollongong, New Illawarra Rd., Lucas Heights, 2234 NSW Australia.*

[f]*Chemical Crystallography, Department of Chemistry, University of Oxford, 12 Mansfield Road, Oxford, OX1 3TA, UK.*

**Corresponding author. E-mail: m.crimmin@imperial.ac.uk*

† Current address: GSK Medical Research Centre, Gunnels Wood Road, Stevenage, SG1 2NY, UK.

Table of contents

1	General Experimental	S-3
2	Experimental Results	S-4
3	X-ray Crystallographic Data	S-10
4	Neutron Diffraction Study for **7c-d$_2$**	S-17
5	Multinuclear NMR Spectroscopy Data	S-20
6	Solid State NMR Spectroscopy Data	S-35
7	Computational Details	S-40

1. General Experimental, Preparation of Materials and Instruments

1.1 Materials

Mes-*BDI*AlH_2 (**1a**), Dipp-*BDI*AlH_2 (**1b**), 2,6-Xylyl-*BDI*AlH_2 (**1c**), Ph-*BDI*AlH_2 (**1e**) and Dep-*BDI*AlH_2 (**1f**) were synthesised by the literature procedures (Ar-*BDI* = $\{(ArNCMe)_2CH\}^-$, Ar = 2,4,6-$Me_3C_6H_2$ (Mes), 2,6-$^iPr_2C_6H_3$ (Dipp), 2,6-$Me_2C_6H_3$ (2,6-Xylyl) or 2,6-$Et_2C_6H_3$ (Dep)).[1] [CpPd(η^3-C_3H_4Ph)] was synthesised as deep red crystals from $PdCl_2$ in a 2 step procedure (via [Pd(η^3-C_3H_4Ph)(μ-Cl)]$_2$) based on literature reactions.[2] [Pd(PCy_3)$_2$] was synthesised as an analytically pure pale brown crystalline solid from [CpPd(η^3-C_3H_4Ph)] in a single step reaction.[3] [CpPd(IMes)(η^1-C_3H_4Ph)] (IMes = 1,3-bis(2,4,6-trimethylphenyl)imidazol-2-ylidene) was synthesised as crude mixture of isomers from the reaction of [CpPd(η^3-C_3H_4Ph)] and isolated IMes in toluene and precipitation from hexane at -35 °C. [Ru(H)$_2$(N_2)$_2$(PCy_3)$_2$] was synthesised from the literature procedure.[4] All other reagents were purchased from common suppliers (Sigma-Aldrich, Merck, Alfa Aesar, TCI etc.) and used without further purification.

Solvents for air sensitive procedures (toluene, diethyl ether, *n*-hexane, *n*-heptane, cyclohexane) were dried using a solvent purification system (SPS) and stored over activated 3 Å molecular sieves under an inert atmosphere of N_2 or argon before use. C_6H_6 (Sigma-Aldrich anhydrous grade), C_6D_6 and toluene-D_8 were degassed by the freeze-pump-thaw method (x 3) and stored under inert atmosphere over activated 3 Å molecular sieves.

1.2 Instruments

^{1}H NMR, ^{31}P NMR and ^{13}C NMR spectra were recorded and analysed using Bruker 400 MHz Spectrometer at 298 K. The reported values for ^{1}H, ^{31}P NMR and ^{13}C NMR data are as follows: chemical shifts (δ ppm), multiplicity (where s = singlet, d = doublet, t = triplet, m = multiplet), integration (not ^{13}C) and coupling constant, J (Hz). AT-IR spectra were recorded on an Agilent Technologies Cary 630 FTIR spectrometer. Elemental analysis was carried out by Stephen Boyer, London Metropolitan University.

[1] (a) S. Yow, S. J. Gates, A. J. P. White, M. R. Crimmin, *Angew. Chem. Int. Ed.,* 2012, **51**, 12559. (b) C. Cui, H.W. Roesky, H. Hao, H-G. Schmidt, M. Noltemeyer, *Angew. Chem., Int. Ed.* 2000, **39**, 1815. (c) Z. Yang, M. Zhong, X. Ma, K. Nijesh, S. De, P. Parameswaran, H.W. Roesky, *J. Am. Chem. Soc.* 2016, **138**, 2548.

[2] (a) P. R. Auburn, P. B. Mackenzie, B. Bosnich, *J. Am. Chem. Soc.*, 1985, **107**, 2033. (b) S. D. Robinson , B. L. Shaw, *J. Chem. Soc.*, 1963, 4806.

[3] T. Yoshida, S. Otsuka, Inorg. Synth., 1990, **28**, 114.

[4] (a) M.L. Christ, S. Sabo-Etienne, G. Chung, B. Chaudret, *Inorg. Chem*., 1994, **33**, 5316. (b) S. Lau, B.J. Ward, X Zhou, A.J.P. White, I.J. Casely, S. Macgregor, and M.R. Crimmin, *Organometallics*, 2017, **36**, 3654.

2. Experimental Results

2.1 Synthesis of Aluminium Dihydrides

2.1.1 Synthesis of 3,5-Xylyl-*BDI*AlH_2 (1d)

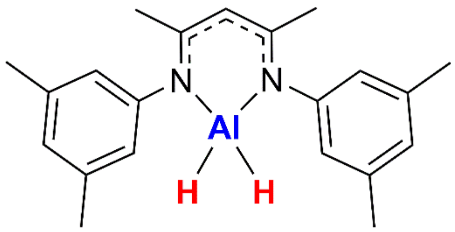

Following the literature procedure used to synthesise **1a** and **1c**,[5] in a glovebox, β-diketiminate pro-ligand (1 equiv.) and $LiAlH_4$ (1.2 equiv.) were weighed into two separate Schlenk flasks. These two flasks were then sealed and removed from the glovebox, then attached to a Schlenk line. Under argon, diethyl ether was added to both of them to make up ≈ 0.5 M solutions. The solution of pro-ligand was transferred to the solution of $LiAlH_4$ portion-wise via a cannula at -78 °C. The mixture was then allowed to warm to room temperature and H_2 gas evolution was observed along with the formation of a colourless precipitate. After stirring overnight, the precipitate was allowed to settle for 2 hours. After filtering the mixture via a cannula, a clear pale yellow filtrate was obtained, which was then concentrated to half the volume and stored at -21 °C overnight. The product crystallised and filtration and drying *in vacuo* gave **1d** as a colourless crystalline solid. Isolated as a single crop of colourless crystals (0.41 g, 1.23 mmol, 15% yield based upon 8.5 mmol of pro-ligand). **^{1}H NMR (400 MHz, 298 K, C_6D_6) δ**: 6.83 (s, 4H), 6.61 (s, 2H), 4.82 (s, 1H), 2.01 (s, 12H), 1.67 (s, 6H) ppm. **^{13}C{^{1}H} NMR (101 MHz, 298 K, C_6D_6) δ**: 168.5, 145.1, 139.1, 123.3, 97.3, 22.3, 20.8 ppm. **Elemental analysis**: calc. for $C_{21}H_{26}AlN_2$ – C 75.42%, H 8.14%, N 8.38%; found – C 75.51%, H 8.25%, N 8.50%. **AT-IR (ν/cm^{-1})**: 1819, 1782.

2.1.2 Synthesis of Ph-*BDI*AlH_2 (1e)

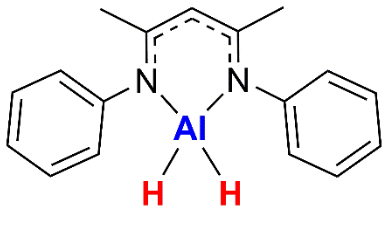

1e has been reported previously but we have used a procedure adapted slightly from literature.[6] In a glovebox, β-diketiminate pro-ligand (2.00 g, 8.0 mmol) and $AlH_3.NMe_3$ (0.71 g, 8.0 mmol) were weighed into two separate Schlenk flasks. These two flasks were then sealed and removed from the glovebox, then attached to a Schlenk line. Under argon, pentane was added to both of them to make up ≈ 0.5 M solutions. The solution of $AlH_3.NMe_3$ was cooled down to -78 °C, and the solution of pro-ligand was transferred in portion-wise via a cannula. The mixture was

[5] S. Yow, S. J. Gates, A. J. P. White, M. R. Crimmin, *Angew. Chem. Int. Ed.,* 2012, **51**, 12559.
[6] N. Kuhn, S. Fuchs, M. Steimann, *Z. Anorg. Allg. Chem.*, 2000, **626**, 1387.

then allowed to warm to room temperature and H_2 gas evolution was observed along with the formation of a colourless precipitate. After stirring overnight, the mixture was concentrated to dryness. Addition of diethyl ether (60 mL) resulted in a yellow solution with a colourless precipitate. After filtering the mixture via a cannula, the clear yellow filtrate was concentrated to half the volume and stored at -21°C overnight. The product crystallised and filtration and drying *in vacuo* gave the product **1e** as yellow crystals (0.55 g, 1.98 mmol, 25%). Spectroscopic data for **1e** matched that previously reported.[6]

2.2 Synthesis of Heterometallic Products

2.2.1 Synthesis of [RuH$_2$(PCy$_3$)$_2$(Mes-*BDI*AlH$_2$)] (2)

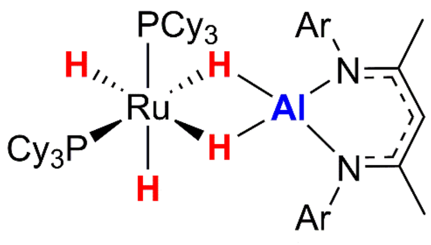

Ar = Mes

In a dinitrogen glovebox Mes-*BDI*AlH$_2$ (5.0 mg, 0.01 mmol) and [Ru(H)$_2$(N$_2$)$_2$(PCy$_3$)$_2$] (10 mg, 0.01 mmol) were weighed into a scintillation vial and dissolved in C_6D_6 (600 μL) and then transferred into a Young's NMR tube. Effervescence was observed along with a colour change from faint yellow to orange. Isolation of **2** as a solid was not possible due to instability and formation of a related product **2a** through a dimerisation pathway at 25 °C. While it was possible to identify **2a** as a result of dimerisation of **2** (NMR data below, section 2.2.2), it was not possible to unambiguously identify the structure.

***In situ* ^{1}H NMR (Tol-D$_8$, 500 MHz, 213 K) δ**: -9.32 (br s, 1H, Ru–*Ht*), -9.91 (br s, 1H, Ru–*Ht*), -12.45 (br s, 1H, Ru–*Hμ*), -13.86 (br s, 1H, Ru–*Hμ*) ppm. ***In situ* ^{1}H NMR (Tol-D$_8$, 500 MHz, 298 K) δ:** 6.84 (s, 4H, Ar*H*), 4.98 (s, 1H, C*H*), 2.32 (s, 12H, ArC*H$_3$*), 2.20 (s, 6H, ArC*H$_3$*), 2.11–2.02 (m, 18H, Cy), 1.89–1.81 (m, 12H, Cy), 1.77–1.70 (m, 6H, Cy), 1.51–1.45 (m, 12H, Cy), 1.37 (s, 6H, CC*H$_3$*), 1.33–1.16 (m, 18H, Cy), -8.78 – -14.61 (br s, 4H, Ru–*H*) ppm. **T_1(min) (Tol-D$_8$, 313 K, 400 MHz)** = 247 ms (Ru–H). ***In situ* ^{13}C{^{1}H} NMR (C_6D_6, 126 MHz, 298 K) δ:** 170.5 (*C*CH$_3$), 142.8 (*Cp*), 135.3 (*Ci*), 133.1 (*Co*), 130.0 (*Cm*), 100.3 (*C*H), 40.9 (Cy), 30.7 (Cy), 28.9 (Cy), 27.7 (Cy), 24.1 (C*C*H$_3$), 21.1 (Ar*C*H$_3$), 20.1 (Ar*C*H$_3$) ppm. ***In situ* ^{31}P{^{1}H} NMR (Tol-D$_8$, 162 MHz, 213 K) δ:** 70.7 (s), 66.2 (s) ppm. ***In situ* ^{31}P{^{1}H} NMR (Tol-D$_8$, 162 MHz, 298 K) δ:** 69.9 (s) ppm. **Elemental analysis**: Due to sensitivity of this complex repeated attempts to acquire CHN analysis failed to provide satisfactory results.

2.2.2 NMR Spectroscopy Data for 2a, Dimerisation Product of 2

^{1}H NMR (Tol-D$_8$, 500 MHz, 298 K) δ: 6.83 (s, 8H, Ar*H*), 5.28 (s, 2H, C*H*), 2.22–2.41 (m, 36H, ArC*H$_3$*), 1.62–1.92 (m, 38H, Cy), 1.50 (s, 12H, CC*H$_3$*), 1.24–1.40 (m, 28H, Cy), -14.20 (br s, 6H, Ru–*H*) ppm. **^{13}C{^{1}H} NMR (Tol-D$_8$, 126 MHz, 298 K) δ:** 168.9 (*C*CH$_3$), 143.8 (*Cp*), 134.3 (*Ci*), 133.7 (*Co*), 129.6 (*Cm*), 102.2 (*C*H), 39.2 (Cy), 29.7 (Cy), 28.6 (Cy), 27.3 (Cy), 23.7 (C*C*H$_3$), 22.2 (Ar*C*H$_3$), 21.0 (Ar*C*H$_3$) ppm. **^{31}P{^{1}H} NMR (Tol-D$_8$, 162 MHz, 298 K) δ:** 74.0 (s) ppm. **T$_1$(min) (Tol-D$_8$, 273 K, 400 MHz)** = 221 ms (Ru–H). **AT-IR (ν/cm^{-1})**: 1610–1654 (Ru–H–Al).

2.2.3 Synthesis of [RuH$_2$(PCy$_3$)(N$_2$)(Dipp-*BDI*AlH$_2$)] (3)

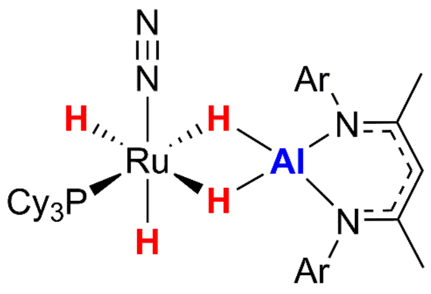

Ar = Dipp

In dinitrogen glovebox Dipp-*BDI*AlH$_2$ (32 mg, 0.07 mmol) and [Ru(H)$_2$(N$_2$)$_2$(PCy$_3$)$_2$] (50 mg, 0.07 mmol) were weighed into a scintillation vial and dissolved in toluene (2 mL) then left to stir at 25 °C overnight. Solvent was removed *in vacuo* to give an oily residue which was washed with pentane and dried until a yellow powder was obtained. Solid was re-dissolved in a mixture of toluene (1 mL) and hexane (1 mL) then filtered through micro-glass fibre and left in the glovebox freezer (–35 °C) overnight to give orange solid as product **3** (24 mg, 40%).

^{1}H NMR (C$_6$D$_6$, 400 MHz, 298 K) δ: 7.14 (m, 6H, Ar*H*), 5.02 (s, 1H, C*H*), 3.18 (sept, $^3J_{HH}$ = 6.7 Hz, 4H, C*H*(CH$_3$)$_2$), 1.94–2.04 (m, 6H, Cy), 1.69–1.80 (m, 8H, Cy), 1.58 (d, $^3J_{HH}$ = 6.8 Hz, 12H, CH(C*H$_3$*)$_2$), 1.54 (s, 6H, CC*H$_3$*), 1.18–1.35 (m, 19H, Cy), 1.09 (d, $^3J_{HH}$ = 6.8 Hz, 12H, CH(C*H$_3$*)$_2$), -9.05 (br s, 2H, Ru–*H*), -9.73 (d, $^2J_{HP}$ = 41.6 Hz, 1H, Ru–*H*), -16.12 (d, $^2J_{HP}$ = 15.8 Hz, 1H, Ru–*H*) ppm. **^{13}C{^{1}H} NMR (C$_6$D$_6$, 125 MHz, 298 K) δ:** 171.5 (*C*CH$_3$), 143.7 (*Co*), 140.0 (N*Ci*), 124.7 (ArH), 99.1 (*C*H), 37.8 (d, $^1J_{CP}$ = 17.2 Hz, Cy*i*), 30.4 (Cy), 29.3 (*C*H(CH$_3$)$_2$), 28.5 (Cy), 27.2 (Cy), 25.0 (CH(*C*H$_3$)$_2$), 24.7 (CH(*C*H$_3$)$_2$), 24.3 (C*C*H$_3$) ppm. **^{31}P^1{H} NMR (C$_6$D$_6$, 162 MHz, 298 K) δ:** 73.6 (s) ppm. **T$_1$(min) (Tol-D$_8$, 233 K, 400 MHz)** = 198 ms (δ$_H$ = -7.83 ppm), 224 ms (δ$_H$ = -9.66 ppm), 220 ms (δ$_H$ = -10.49 ppm) and 246 ms (δ$_H$ = -16.07 ppm). **AT-IR (ν/cm^{-1})**: 2135 (N≡N), 1908 and 1872 (Ru–H). **Elemental Analysis**: Calc. for $C_{65}H_{111}AlN_2P_2Ru$ – C 65.78%, H 9.16%, N 6.53%; found – C 66.46%, H 8.75%, N 5.69%.

2.2.4 Synthesis of [H_2Pd(Dipp-*BDI*Al)]$_2$ (5)

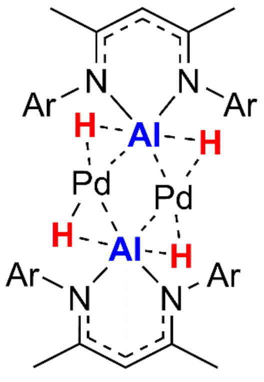

Ar = Dipp

[CpPd(η^3-C_3H_4Ph)] (10.0 mg, 0.0347 mmol) and Dipp-*BDI*AlH_2 (32.5 mg, 0.0729 mmol, 2.1 equiv.) were dissolved in benzene (0.5 mL) to form a red/brown solution which was left to stand at 25 °C in the glove box for 72 h. Red crystals of **5** formed and the mother liquor was decanted, the solid washed with benzene (3 x 0.25 mL) and dried *in vacuo* (10.1 mg, 53%).

Sparing solubility of the isolated crystalline material of **5** in common NMR spectroscopy solvents (benzene, toluene) prohibited full analysis by solution phase NMR spectroscopy of purified **5**.

^{1}H NMR (C_6D_6, 400 MHz, 298 K) δ: 7.04 (d, $^3J_{HH}$ = 7.7 Hz, 4H, Ar*H*), 4.81 (s, 1H, C*H*), 3.30 (sept, $^3J_{HH}$ = 6.8 Hz, 4H, C*H*(CH_3)$_2$), 1.45 (s, 6H, CC*H*$_3$), 1.27 (d, $^3J_{HH}$ = 6.8 Hz, 12H, CH(C*H*$_3$)$_2$), 1.10 (d, $^3J_{HH}$ = 6.8 Hz, 12H, CH(C*H*$_3$)$_2$) ppm. Peak for additional Ar-H (2H) obscured by residual solvent resonance. **^{1}H{^{27}Al} NMR (C_6D_6, 400 MHz, 298 K) δ**: -2.17 (br (FWHM = ~50 Hz) Al··*H*··Pd) ppm. **Elemental analysis**: calc. for $C_{58}H_{86}Al_2N_4Pd_2$ – C 62.96%, H 7.84%, N 5.07%; found – C 62.92%, H 7.94%, N 4.95%. **AT-IR (ν/cm^{-1})**: 3060, 2959, 2926, 2865, 1525, 1457, 1439, 1388, 1315, 1251, 1176, 1100, 1016, 935, 901, 867, 797, 759, 736.

2.2.5 Synthesis of [H_6Pd_4(Mes-*BDI*Al)$_4$] (6)

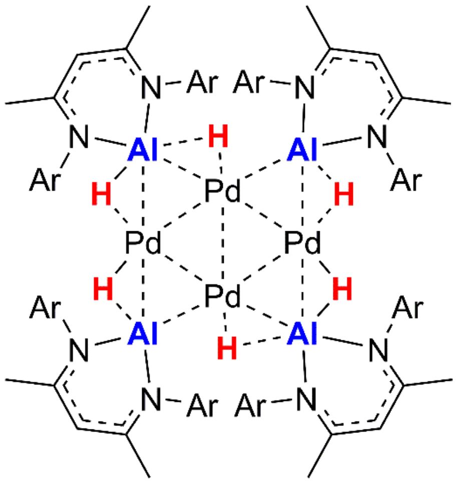

Ar = Mes

[CpPd(η^1-C_3H_4Ph)(IMes)] (35.0 mg, 0.0591 mmol) and Mes-*BDI*AlH_2 (**1**, 42.8 mg, 0.118 mmol) were dissolved in benzene (1 mL) to form a red solution which was transferred to an ampoule and sealed. The ampoule was heated to 50 °C for 64 h without stirring. Red crystals of **6** formed slowly. After cooling the mother liquor was decanted [Pd(IMes)$_2$] could be detected by NMR spectroscopy in the mother liquor), the solid washed with benzene (3 x 1 mL) and dried *in vacuo* (13 mg, 47% based on Pd).

Due to the insolubility of **6** in all common solvents satisfactory NMR spectroscopy data could not be collected. **Elemental analysis**: calc. for $C_{92}H_{122}Al_4N_8Pd_4$ – C 58.96%, H 6.57%, N 5.98%; found – C 59.15%, H 6.69%, N 5.86%. **AT-**

IR (ν/cm^{-1}): 2995, 2915, 2853, 1623, 1609, 1529, 1476, 1450, 1389, 1247, 1200, 1147, 1016, 861, 748, 727.

2.2.6 Synthesis of [H{Pd(PCy$_3$)}(2,6-Xylyl-*BDI*Al)]$_2$ (7c)

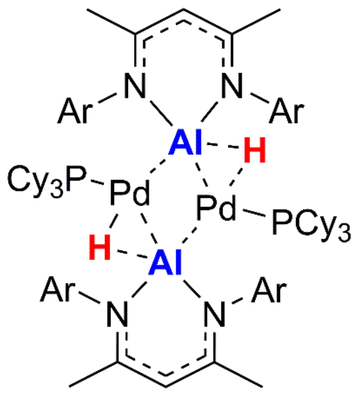

Ar = 2,6-Xylyl

[Pd(PCy$_3$)$_2$] (10.0 mg, 0.015 mmol, 1.0 equiv.) and 2,6-Xylyl-*BDI*AlH$_2$ (5.0 mg, 0.015 mmol, 1.0 equiv.) were dissolved in benzene (0.6 mL) to form a red/brown solution which was left to stand at 25 °C in the glove box for 72 h. Red crystals of **7c** formed and the mother liquor was decanted, the solid washed with hexane (3 x 0.25 mL) and dried *in vacuo* (6.1 mg, 56%).

Insolubility of the isolated crystalline material of **7c** in common NMR spectroscopy solvents (benzene, toluene) prohibited full analysis by solution phase NMR spectroscopy of purified **7c**. Single crystal X-ray and neutron diffraction were used to characterise **7c** in the solid-state.

2.2.7 Reaction of [Pd(PCy$_3$)$_2$] with 1e: Identified Decomposition pathway

The reaction of [Pd(PCy$_3$)$_2$] with **1e** produced a mixture of species. The hypothetical complex **7e** (equivalent to the isolated **7c**) could not be experimentally isolated. However, a related decomposition product was isolated in very low yield as a few single crystals. It was not possible to obtain bulk characterisation data for this compound but the X-ray crystal structure of this product (**S1**) showed it to have a similar Pd_2Al_2 motif (the number and location of metal bound hydride ligands could not be unambiguously identified in this structure due to poor X-ray data quality, figure S2.2). As in **7c**, each Pd is ligated by one PCy$_3$ but a reaction has occurred in one of the Al-bound β-diketaminate ligands making this structure different to **7c**. The structure shows C3 has become a tetrahedral sp^3-centre through reduction of the β-diketaminate by hydride transfer (figure S2.1). This decomposition pathway was not observed for the other palladium complexes of aluminium hydrides **1a-c** which we ascribe to the bulky ortho substituents on the aryl groups sterically blocking hydride transfer processes.

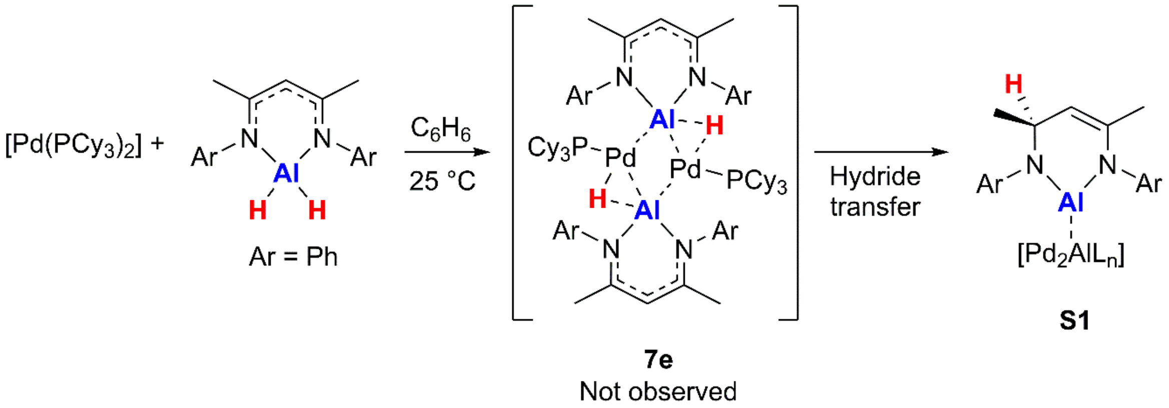

Figure S2.1: Proposed formation of **S1** through decomposition pathway of **7e**.

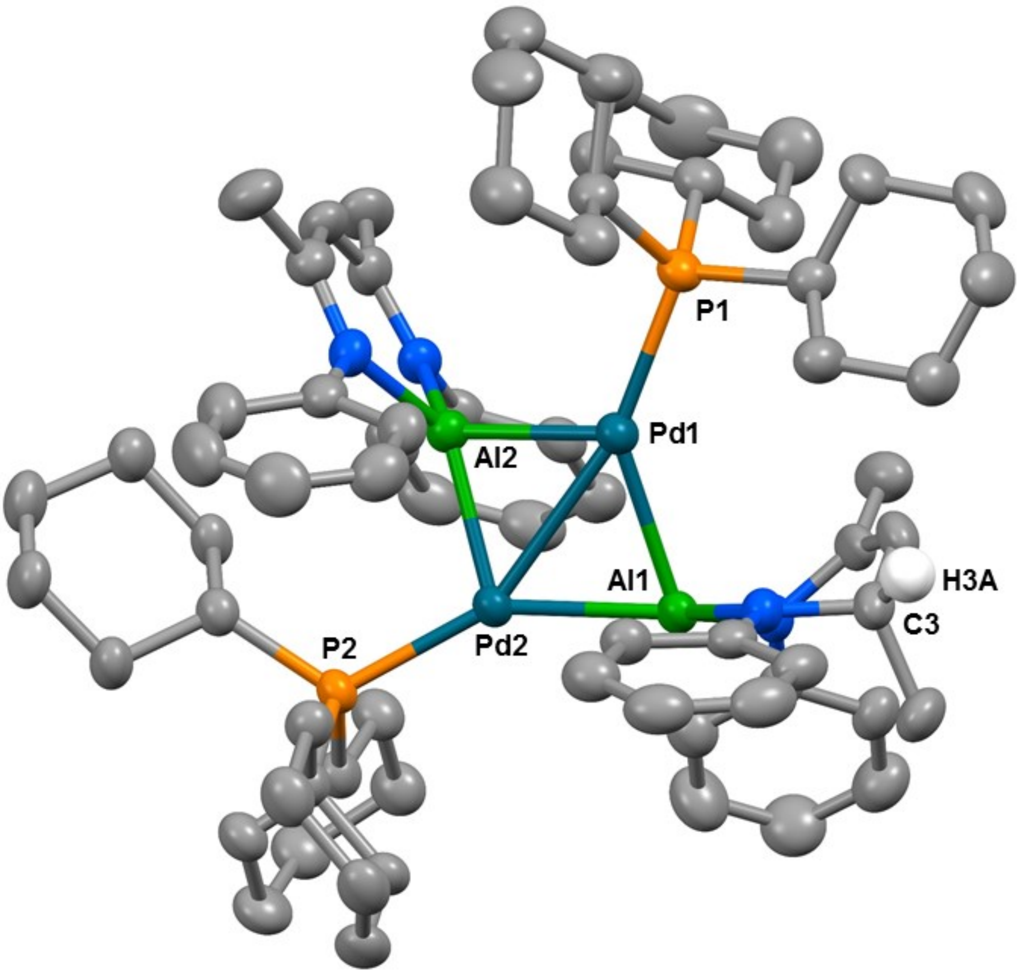

Figure S2.2: The molecule of **S1** (50% probability ellipsoids) modelled from single-crystal X-ray diffraction. Hydrogen atoms and hexane solvent molecules omitted for clarity. Metal bound hydrides were not located in X-ray structure.

3. X-ray Crystallographic Data

3.1 Tabulated X-ray Data

Compound	**3**	**5**
CCDC No.	1908297	1916497
Formula	$C_{47}H_{78}AlN_4PRu$	$C_{58}H_{86}Al_2N_4Pd_2$
M	858.15	1106.06
Crystal System	Triclinic	Monoclinic
Space group	*P*-1 (2)	*C2/c* (15)
T [K]	173(2)	173(2)
a [Å]	10.7298(5)	24.5447(4)
b [Å]	11.8650(5)	15.2883(3)
c [Å]	19.6541(7)	14.8424(2)
α [°]	83.163(3)	90
β [°]	86.568(3)	95.2938(14)
γ [°]	70.525(4)	90
V [$Å^3$]	2341.67(17)	5545.81(15)
Z	2	4
Density [g cm^{-3}]	1.217	1.325
Radiation Used	Mo-Kα	Cu-Kα
μ (mm^{-1})	0.422	5.828
θ range [°]	$2.628 \leq \theta \leq 28.282$	$3.410 \leq \theta \leq 73.780$
Reflns collected	13469	9229
R_{int}	0.0170	0.0229
Completeness (to θ = 25.242°)	98.6%	98.8%
No. of data/restr/param	9230/0/ 513	5369/0/316
R_1 [$I > 2\sigma(I)$]	0.0445	0.0271
wR_2 [all data]	0.1116	0.0700
GoF	1.057	1.035
Largest diff. pk and hole [e$Å^{-3}$]	1.215, -1.103	0.390, -0.546

Table S3.1: Crystal Data, Data Collection and Refinement Parameters for the structures of **3**, **5**, **6**, **7c**.

Compound	**6**	**7c (form 1)**	**7c (form 2)**
CCDC No.	1916498	1908298	1919750
Formula	$C_{92}H_{122}Al_4N_8Pd_4$	$C_{78}H_{118}Al_2N_4P_2Pd_2$	$C_{78}H_{116}D_2Al_2N_4P_2Pd_2$
M	1873.49	1440.46	1442.46
Crystal System	Triclinic	Triclinic	Triclinic
Space group	*P*-1 (2)	*P*-1 (2)	*P*-1 (2)
T [K]	173(2)	173(2)	173(2)
a [Å]	12.1911(5)	12.5660(4)	12.6182(5)
b [Å]	13.8827(7)	13.8938(6)	13.8635(5)
c [Å]	15.1624(6)	14.1192(7)	22.8654(10)
α [°]	106.195(4)	114.468(5)	95.322(3)
β [°]	102.711(3)	101.107(3)	95.111(3)
γ [°]	103.796(4)	101.892(3)	112.036(4)
V [$Å^3$]	2277.47(18)	2085.84(17)	3658.3(3)
Z	1	1	2
Density [g cm^{-3}]	1.366	1.271	1.308
Radiation Used	Mo-Kα	Mo-Kα	Mo-Kα
μ (mm^{-1})	0.864	0.537	0.604
θ range [deg]	$2.477 \leq \theta \leq 28.194$	$2.472 \leq \theta \leq 28.343$	$2.344 \leq \theta \leq 28.218$
Reflns collected	13385	11999	21381
R_{int}	0.0201	0.0114	0.0234
Completeness (to θ = 25.242°)	98.7%	98.6%	98.6%
No. of data/restr/param	9030/0/515	8225/54/463	14430/84/ 868
R_1 [$I > 2\sigma(I)$]	0.0410	0.0235	0.0384
wR_2 [all data]	0.0917	0.0585	0.0822
GoF	1.067	1.045	1.058
Largest diff. pk and hole [e$Å^{-3}$]	0.991, -1.155	0.487, -0.350	0.588, -0.499

Table S3.1 cont: Crystal Data, Data Collection and Refinement for the structures of **3**, **5**, **6**, **7c**.

Table S1 provides a summary of the crystallographic data for the structures of **3**, **5**, **6**, **7c**. Data were collected using Agilent Xcalibur 3 E (**3**, **6**, **7c**) and Xcalibur PX Ultra A (**5**) diffractometers, and the structures were refined using the SHELXTL and SHELX-2014 program systems.[7]

[7] SHELXTL v5.1, Bruker AXS, Madison, WI, 1998. SHELX-2014, G.M. Sheldrick, *Acta Cryst.*, 2015, **C71**, 3-8.

3.2 X-ray crystal structures

3.2.1The X-ray crystal structure of 3

The two bridging Ru–H–Al and two terminal Ru–H hydrogen atoms in the structure of **3** were all located from Δ*F* maps and refined freely.

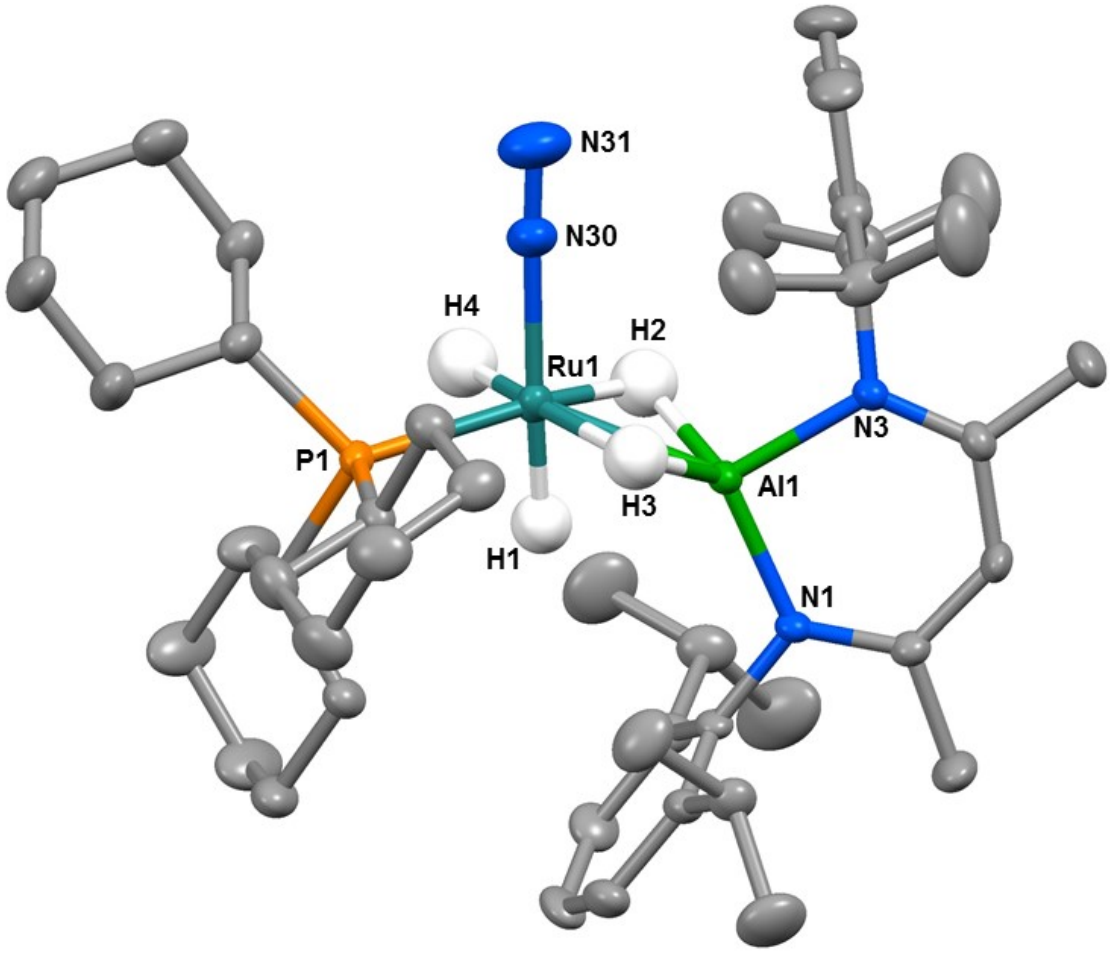

Figure S3.1: The molecule of **3** (50% probability ellipsoids). Selected hydrogen atoms omitted for clarity.

3.2.2 The X-ray crystal structure of 5

The molecule of **5** shows crystallographic C_i symmetry. The bridging Al–H–Pd hydrogen atoms in the structure of **5** were located from a ΔF map and refined freely.

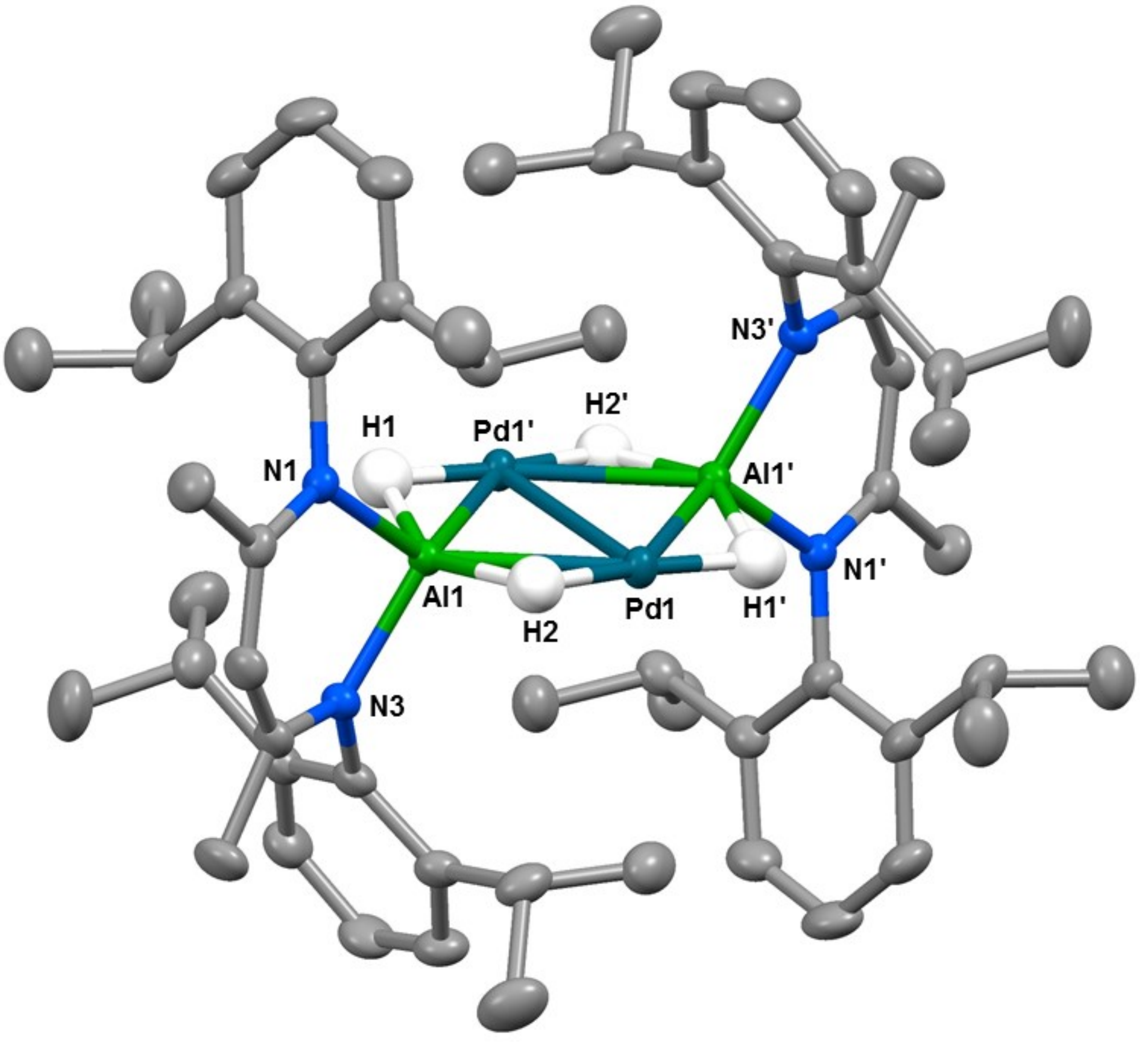

Figure S3.2: The molecule of **5** (50% probability ellipsoids). Selected hydrogen atoms omitted for clarity.

3.2.3 The X-ray crystal structure of 6

The molecule of **6** shows crystallographic C_i symmetry. The bridging Al–H–Pd and hydrogen atoms in the structure of **6** were located from a ΔF map and refined freely.

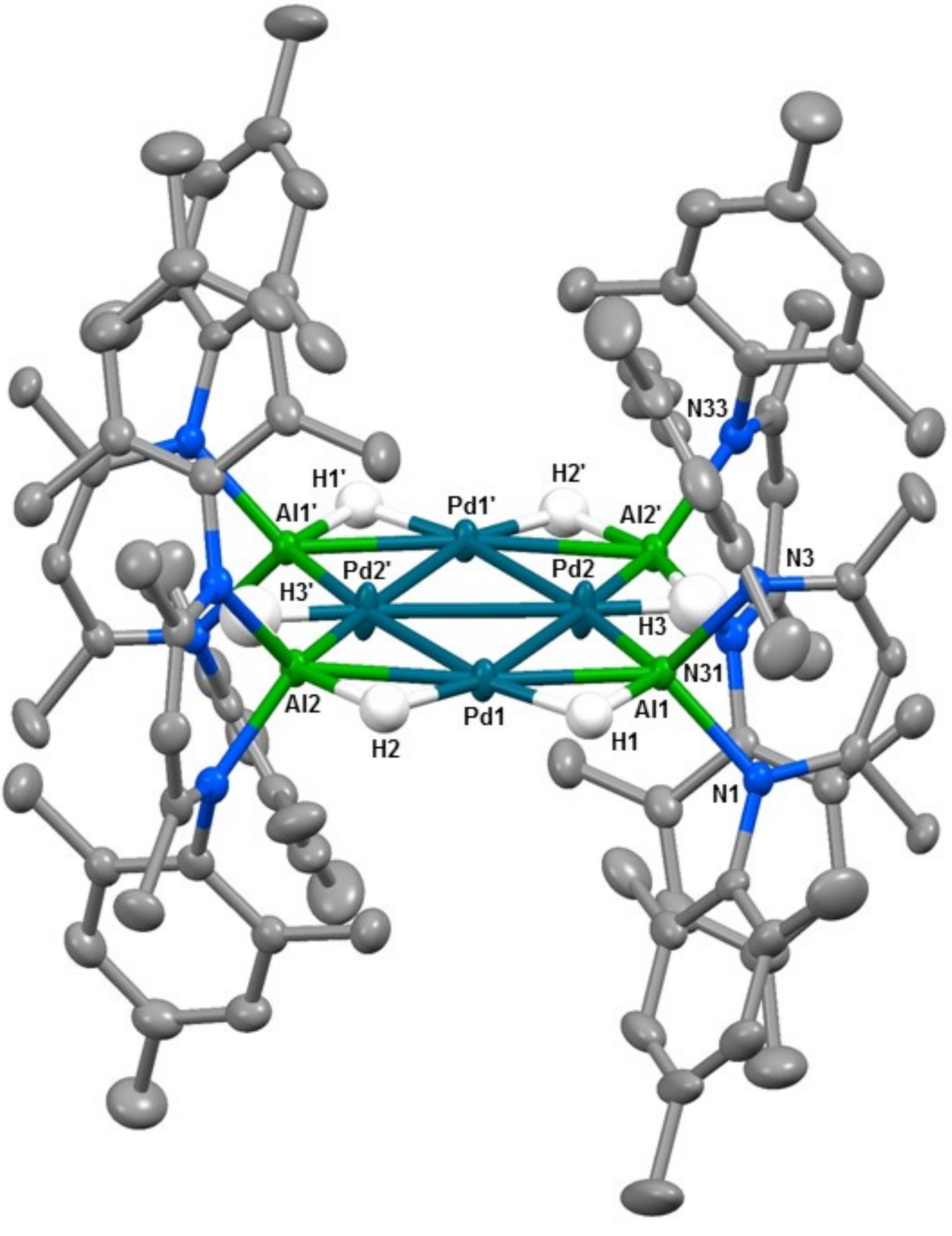

Figure S3.3: The molecule of **6** (50% probability ellipsoids). Selected hydrogen atoms omitted for clarity.

3.2.4 The X-ray crystal structure of 7c (form 1)

The molecule of **7c (form 1)** was found to sit across a centre of symmetry at the middle of the Al_2Pd_2 ring. The unique bridging Al–H–Pd hydrogen atom was located from a ΔF map and refined freely. The included benzene solvent molecule was found to be disordered. Two orientations were identified of *ca*. 79 and 21% occupancy, their geometries were optimised, the displacement parameters of adjacent atoms were restrained to be similar, and only the non-hydrogen atoms of the major occupancy orientation were refined anisotropically (those of the minor occupancy orientation were refined isotropically).

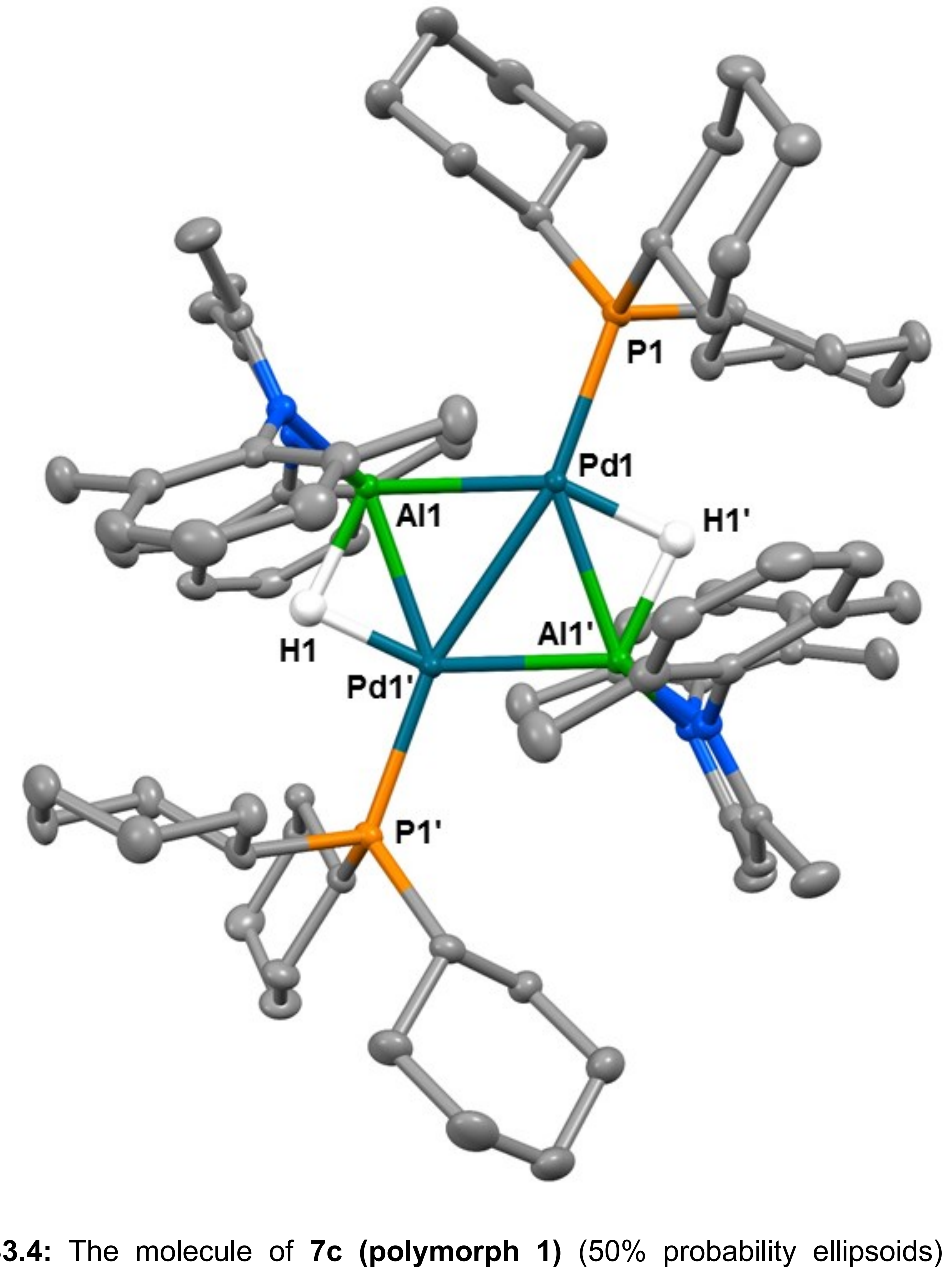

Figure S3.4: The molecule of **7c (polymorph 1)** (50% probability ellipsoids). Selected hydrogen atoms and benzene solvent molecule omitted for clarity, only the major conformer is depicted.

3.2.5 The X-ray crystal structure of 7c (form 2)

The structure of **7c (form 2)** contains 2 half molecules each sitting across a centre of symmetry at the middle of the Al_2Pd_2 ring. The unique bridging Al–H–Pd hydrogen atoms were located from a Δ*F* map and refined freely. One cyclohexyl ring was found to be disordered over 2 positions with the occupancies freely refined to *ca.* 70 and 30%. The C-C bonds in this ring were restrained to 1.54 Å using the DFIX command and the ellipsoids restrained by the ISOR command.

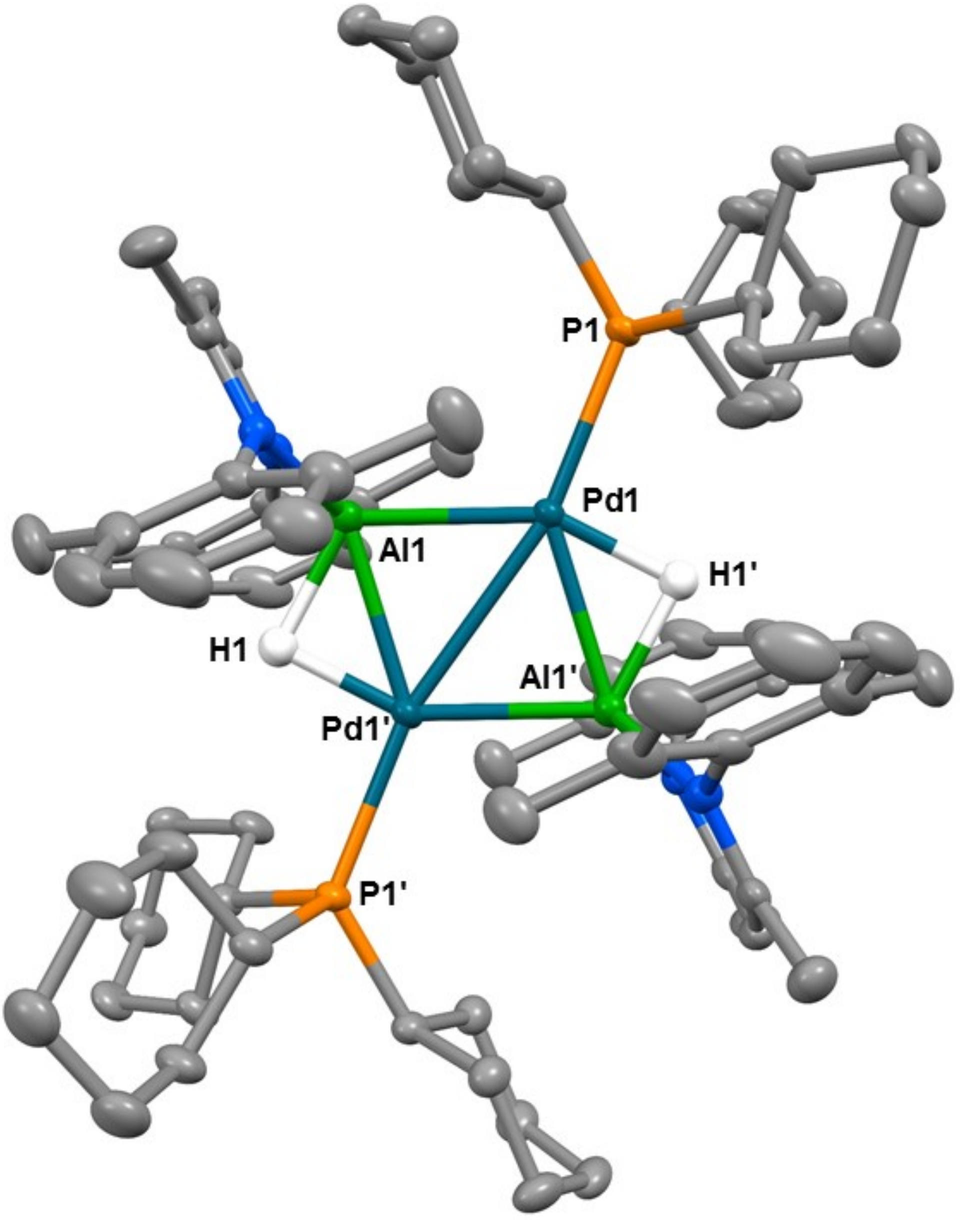

Figure S3.5: One molecule of **7c (form 2)** (50% probability ellipsoids). Selected hydrogen atoms and second molecule of asymmetric unit omitted for clarity.

4. Neutron Diffraction Study for 7c-d_2

The Laue single-crystal neutron diffraction study reported here as each undertaken using the KOALA instrument standing at the end guide position of TG3, a thermal neutron beam produced from the OPAL reactor at the Australian Nuclear Science and Technology Organization. Detection was by means of neutron sensitized 'Niimura special' image plates mounted to the fixed radius cylindrical detector drum. The sample was handled immersed in argon to ensure compound stability while the crystal was transferred to the cold nitrogen stream of an Oxford Cryosystems COBRA™ cryostream. Details of the data collection and reduction procedures are provided in the relevant CIFs. The crystal is of low symmetry possible data from two separate orientations of the unit cell with respect to the phi axis of the instrument were recorded to ensure full coverage of the unique fraction of reciprocal space.

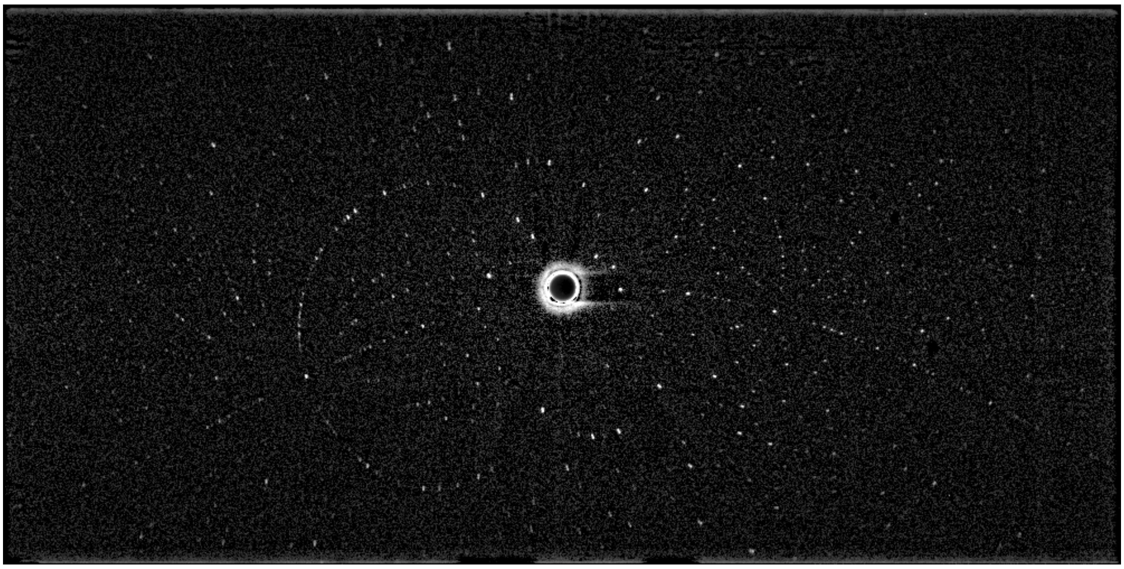

Figure S4.1: A representative Laue image showing the diffraction peaks for which data were extracted for **7c-d_2**

Data were extracted from 35 images each recorded for 5000s and a further 12 images recorded for 8000s by means of the LaueG software.[8] Intensities were recorded for diffraction spots with wavelengths $0.85 < \lambda < 1.70$ Å to a minimum *d* spacing of 1.02 Å. 60469 reflections were extracted of which 6009 were unique and 2736satisfied the $I \geq 3\sigma I$ criterion of observability.

[8] (a) R.O. Piltz, *J. Appl. Cryst.*, 2018, **51**, 635. (b) R.O. Piltz, *J. Appl. Cryst.*, 2018, **51**, 963.

The neutron diffraction data obtained for **7c-d2** has been modelled using the CRYSTALS[9] software package. Atoms of **7c-d2** are modelled with positional parameters included for all atoms. Anisotropic displacement parameters are refined for all non-hydrogen atoms including the deuteriums while the hydrogens are modelled with individual isotropic displacement parameters. Some limited evidence of disorder is apparent for one cyclohexyl group but this remains modelled by anisotropic displacement parameters as no successful alternative model was resolved. Data were supplemented by appropriate distance, angle and displacement parameter restraints to yield a final model refined against F with R = 0.090 R_w = 0.122 S = 1.23, minimum and maximum residual nuclear densities were -1.29 and 1.74 fermi Å^3

Full details of the experiment, data reduction and refinement are contained in the supplementary CIF file for this paper.

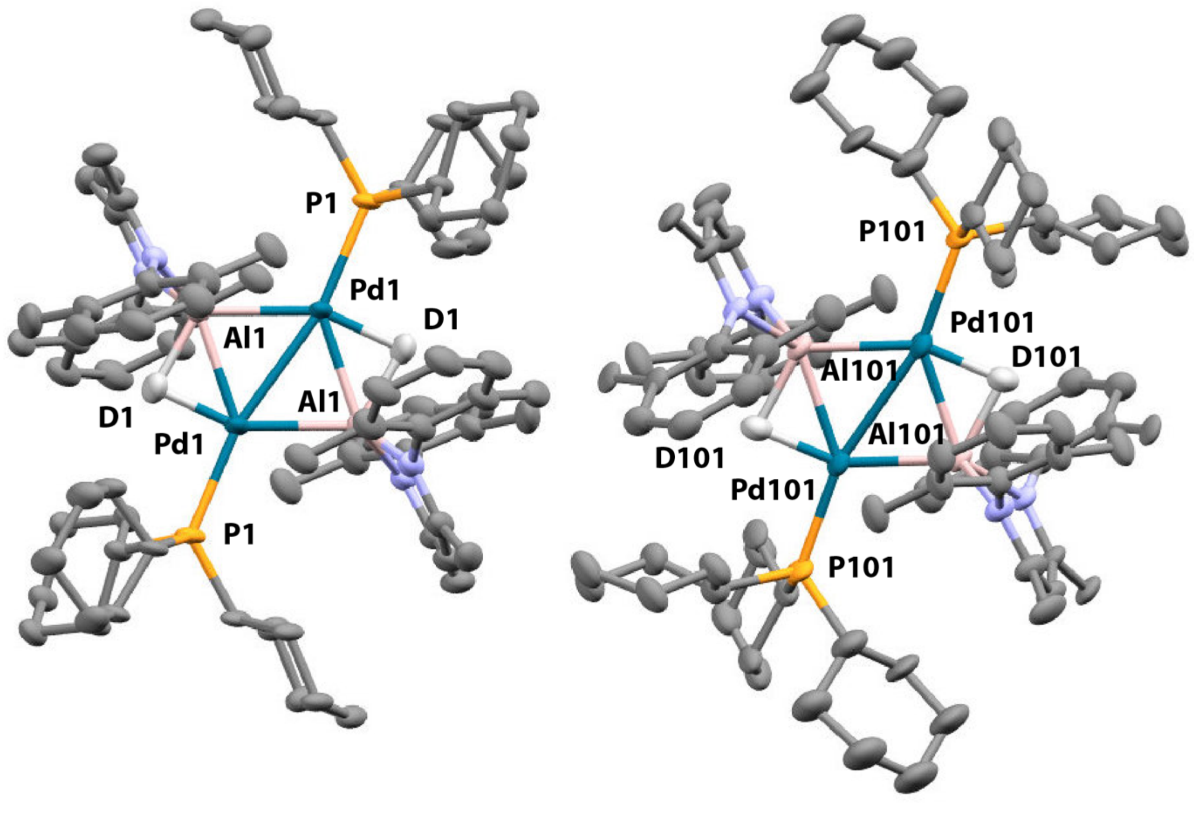

Figure S4.2: The molecular structures of **7c-d₂** determined by neutron diffraction 50% probability ellipsoids**.** Molecule 1 (left) and molecule 2 (right).

[9] P.W. Betteridge, J.R. Carruthers, R.I. Cooper, K. Prout D.J. Watkin, *J. Appl. Cryst.*, 2003, **36**, 1487.

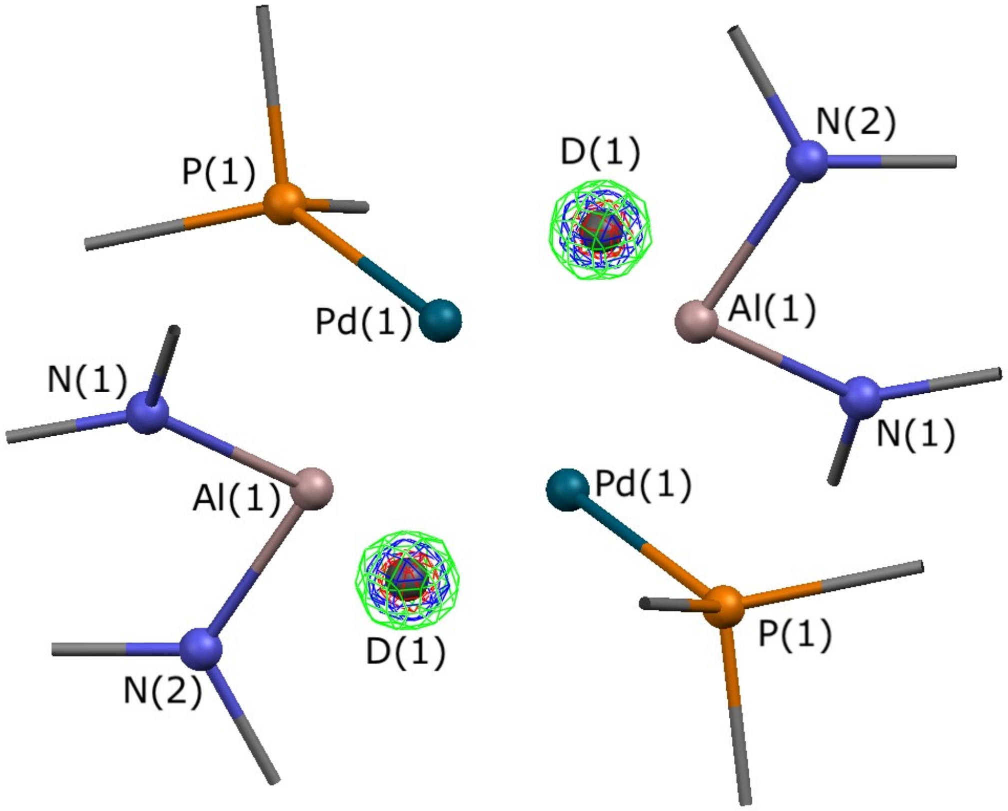

Figure S4.3: Nuclear difference density for the deuterides shown for **7c-d$_2$**, with contours drawn at +3.2, +4.2 and +5.2 fm/Å^3. Selected atoms shown for clarity.

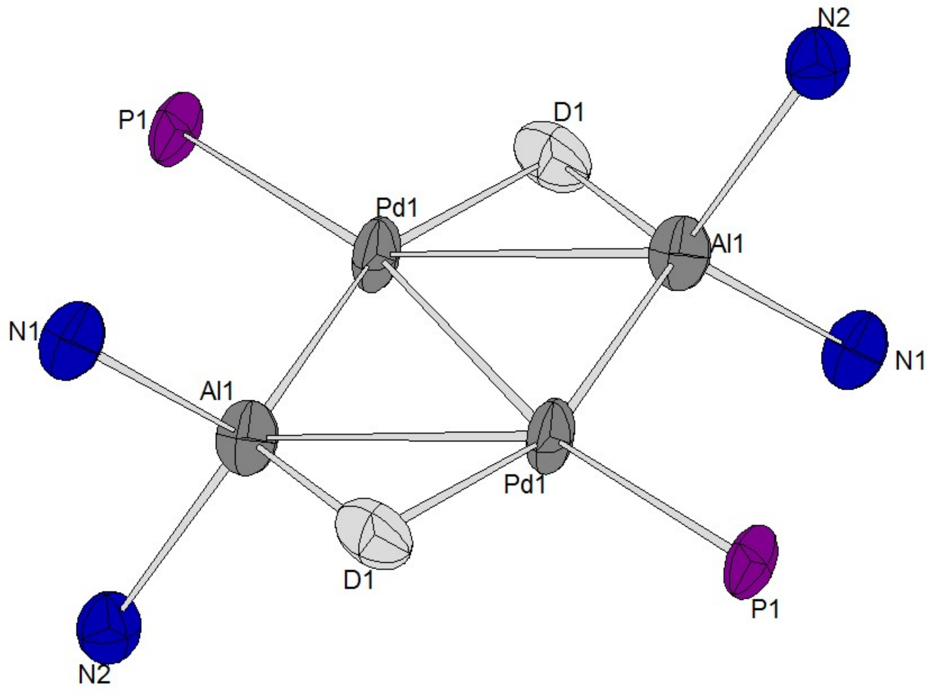

Figure S4.4: Displacement ellipsoid plot (50% probability) showing the metal coordination environments of **7c-d$_2$**.

5. Multinuclear NMR Spectroscopy Data

5.1 Variable Temperature NMR Spectroscopy on Ru bound Al Dihydrides

5.1.1 Characterisation of 2 by Variable Temperature NMR Spectroscopy

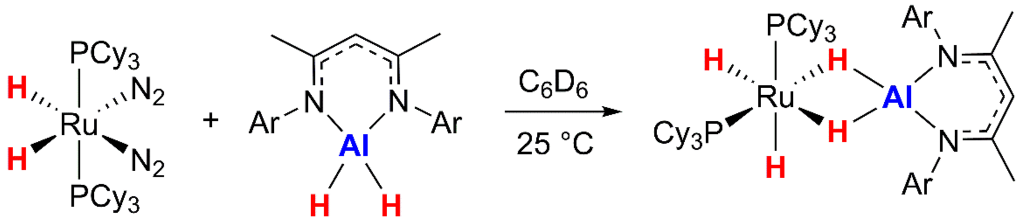

Figure S5.1: In situ synthesis of **2**.

Complex **2** was prepared in situ by the synthesis shown above (figure S5.1) and the ^{1}H NMR spectrum at 293 K showed an indicative broad peak at δ = –11.77 ppm (fwhm = 1021 Hz) for all 4 hydrides (figure S5.2). Upon cooling the reaction to 273 K the broad peak decoalesced into 2 broad peaks at δ = –9.69 (fwhm = 77 Hz) and –13.49 (fwhm = 101 Hz) ppm for the terminal and bridging hydrides respectively. A second fluxional process was further resolved at 213 K with 4 broad peaks observed at δ = –9.32 (H_t, fwhm = 84 Hz), –9.91 (H_t, fwhm = 77 Hz), –12.45 (H_μ, fwhm = 217 Hz) and –13.86 (H_μ, fwhm = 198 Hz) ppm in the ^{1}H NMR spectrum for the 4 magnetically inequivalent hydrides. Long T_1(min) = 247 ms were measured for the hydride resonances at 313 K at 400 MHz (all 4 hydrides were recorded as one resonance at this temperature) indicative of classical hydride behaviour. At 293 K only one resonance was observed in ^{31}P{^{1}H} NMR at δ = 69.9 ppm (figure S5.3) which upon cooling the reaction to 213 K decoalesced into 2 broad peaks observed at δ = 70.7 and 66.2 ppm.

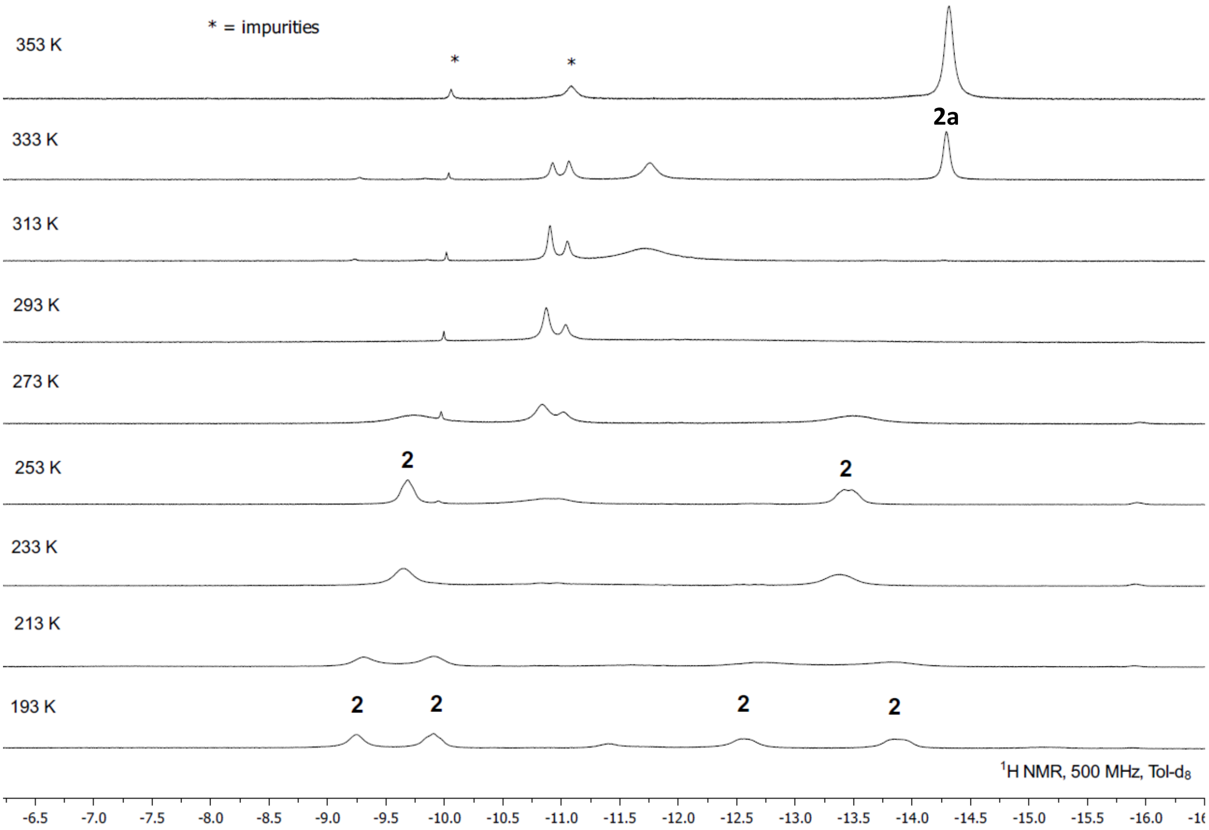

Figure S5.2: VT ^{1}H NMR spectra of a sample of **2** freshly generated from the reaction of **1a** with $[Ru(H)_2(N_2)_2(PCy_3)_2]$ in Tol-D_8. Only Ru–H region shown for clarity.

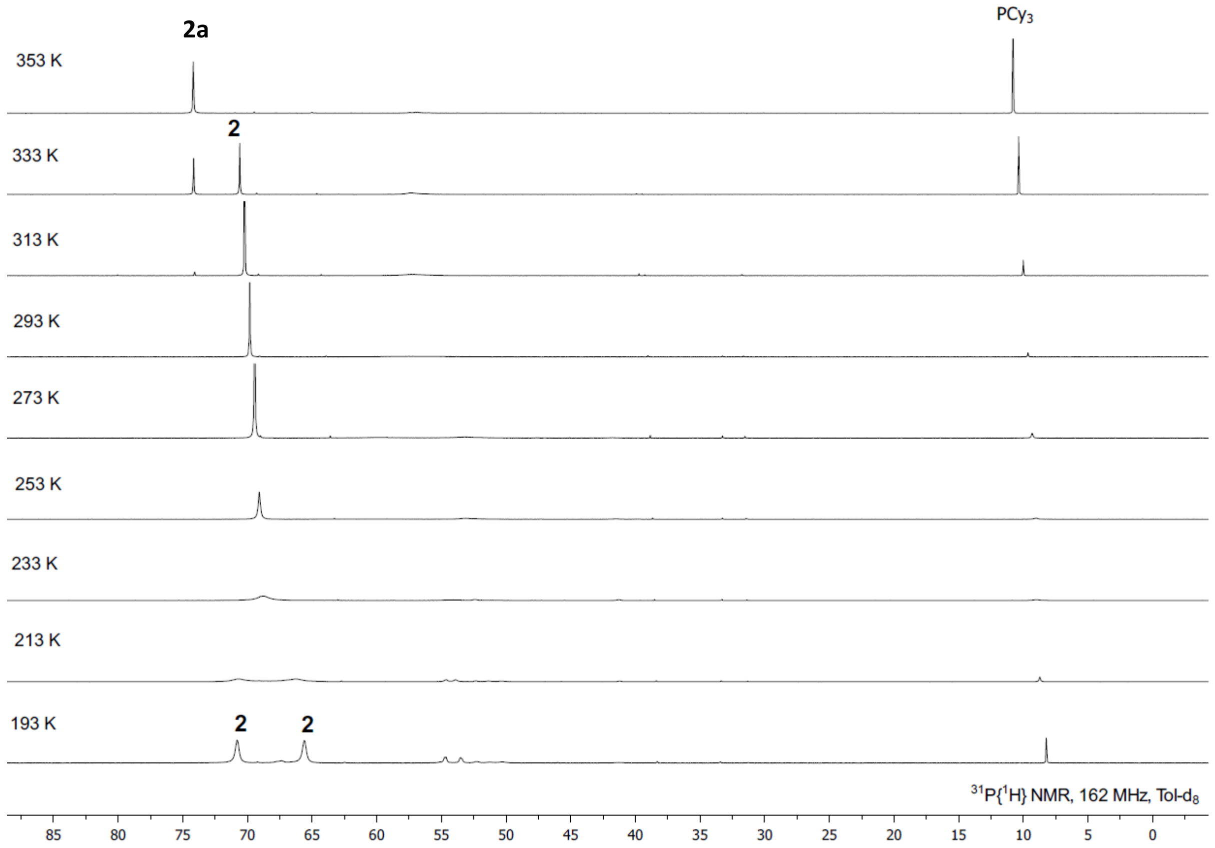

Figure S5.3: VT $^{31}P\{^1H\}$ NMR spectra of a sample of **2** freshly generated from the reaction of **1a** with $[Ru(H)_2(N_2)_2(PCy_3)_2]$ in Tol-D$_8$.

VT NMR data was fitted using line shape analysis with the DNMR programme integrated into Topspin v3.1. The ^1H and ^{31}P resonances of **2** were fitted over the 193 to 253 K range with an initial line broadening factor of 20 Hz for ^{31}P NMR and 2 Hz for ^1H. Fits for *k* were optimized to the experimental data with reasonable accuracy and the modelled data are presented below, a minor unassigned species observable was not included in the model. The activation parameters for the exchange process are as follows:

^{31}P $\Delta H^{\ddagger}$ = 10.8 ± 1.1 kcal mol^{-1}, $\Delta S^{\ddagger}$ = +6.6 ± 5.2 cal K^{-1} mol^{-1}, $\Delta G^{\ddagger}_{298\ K}$ = 8.8 ± 2.7 kcal mol^{-1}.

^{1}H $\Delta H^{\ddagger}$ = 10.7 ± 0.8 kcal mol^{-1}, $\Delta S^{\ddagger}$ = +6.2 ± 7.2 cal K^{-1} mol^{-1}, $\Delta G^{\ddagger}_{298\ K}$ = 8.9 ± 2.9 kcal mol^{-1}.

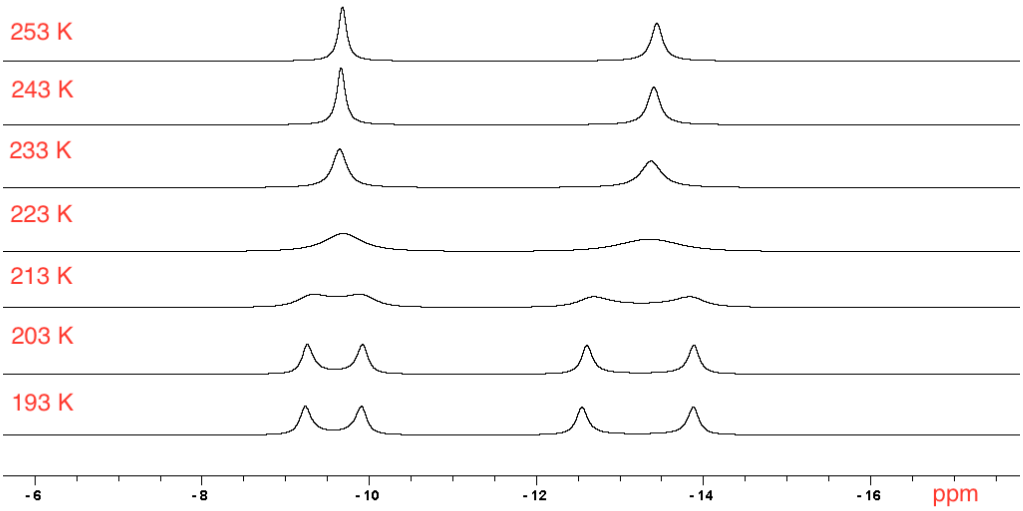

Figure S5.4: Modelled VT ^{1}H NMR spectra of **2**.

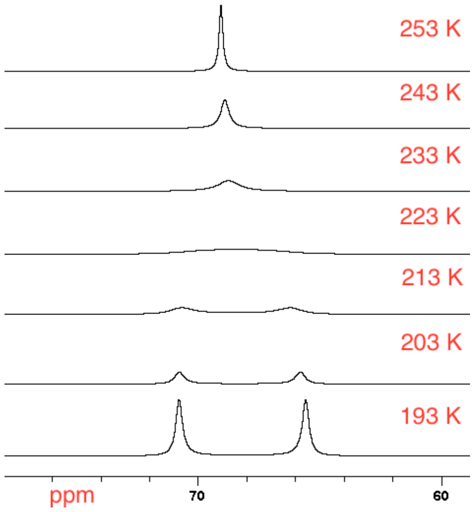

Figure S5.5: Modelled VT ^{31}P{^{1}H} NMR spectra of **2**.

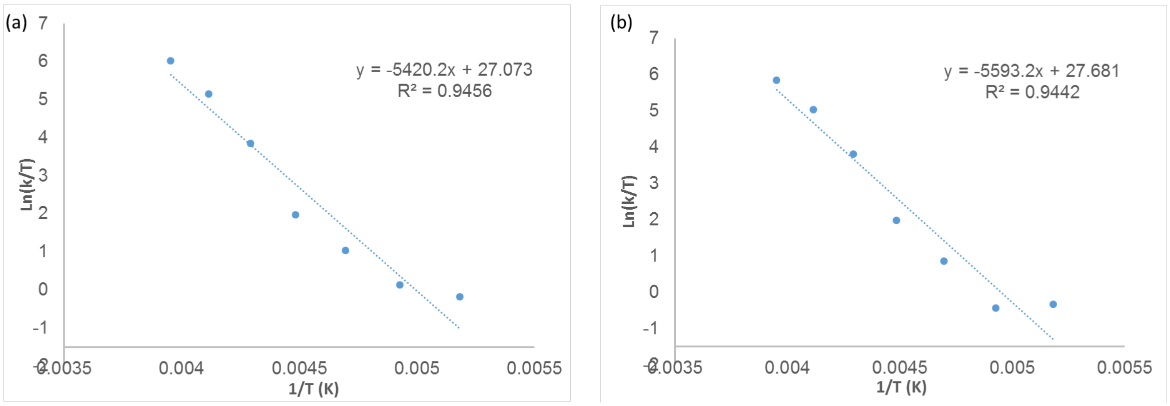

Figure S5.6: Eyring analysis: (a) Modelled VT ^{31}P{^{1}H} NMR data, (b) Modelled VT ^{1}H NMR data**.**

The low temperature NMR spectroscopy data agreed with the assignment of the structure of **2** whereby the PCy_3 ligands were in a cis-arrangement around the ruthenium, one in the axial and one in the equatorial position to give magnetically inequivalent phosphines. The positive entropy of activation (with the caveat of the error associated with the Eyring analysis) potentially suggests that the mechanism of exchange was through a dissociative process whereby the heterobimetallic complex could break apart to give just the aluminium and ruthenium fragments to allow for the rearrangement of the ligands around the ruthenium centre via C_3 rotation. No coupling between the hydrides and phosphines were observed due to the proximity of the hydrides to the quadrupolar aluminium nuclei and the facile exchange process of the hydrides within the NMR spectroscopy acquisition time.

5.1.2 Characterisation of 3 by Variable Temperature NMR Spectroscopy

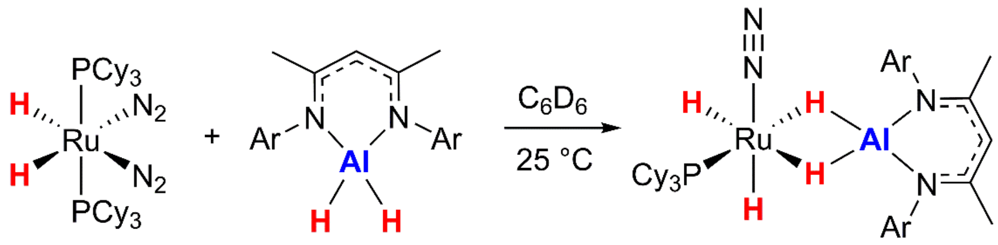

Figure S5.7: Synthesis of **3**.

Complex **3** (figure S5.7) was prepared by the synthesis described in section 2.2.3. A sample of isolated complex **3** in toluene-d_8 demonstrated 3 resonances for the hydride environments at 25 °C in the ^{1}H NMR spectrum (figure S5.8), δ = –9.05 (br s, 2H), –9.73 (d, J_{HP} = 41.6 Hz, 1H), –16.11 (d, J_{HP} = 15.8 Hz) ppm. At 233 K, the resonance at δ = –9.05 ppm was resolved into two peaks at δ = –7.83 and –10.49 ppm to overall give 4 resonances for the 4 magnetically inequivalent hydrides in **3**. T_1 measurements were taken across the 333 – 193 K range at 400 MHz and the 4 hydride signals exhibited a T_1(min) between 200 – 250 ms which indicated more classical hydride behaviour for all 4 hydrides. Following the reaction by ^{31}P{^{1}H} NMR spectroscopy the signal for free PCy_3 was observed as well as one new resonance at δ = 73.6 ppm and this new signal exhibited no decoalescence during low temperature NMR spectroscopy experiments (figure S5.9).

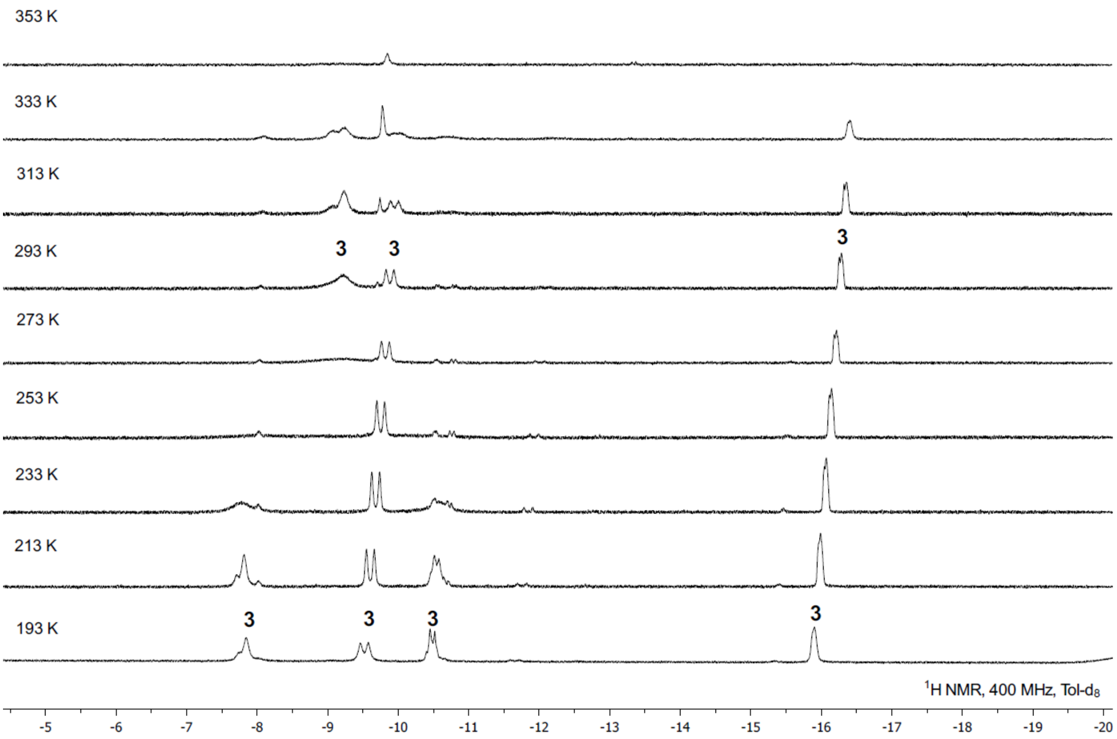

Figure S5.8: VT ^{1}H NMR spectra of a sample containing **3** in Tol-D$_8$. Only Ru–H region shown for clarity.

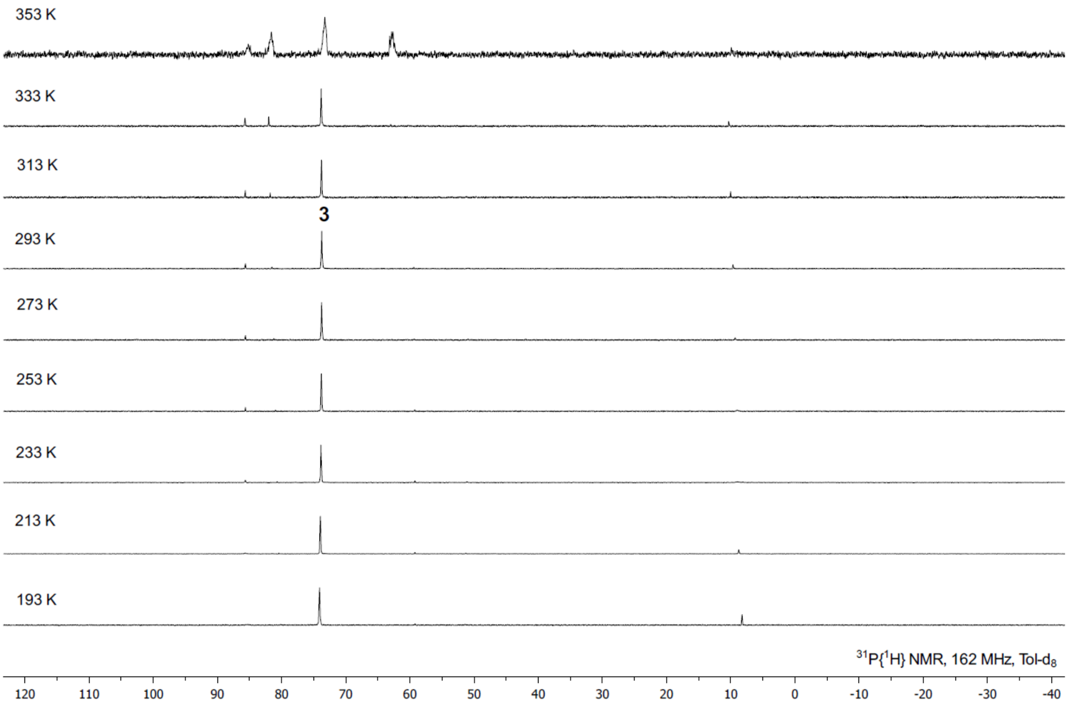

Figure S5.9: VT ^{31}P{^{1}H} NMR spectra of a sample containing **3** in Tol-D$_8$.

5.2 Characterisation of 4

5.2.1 Characterisation of 4 by Variable Temperature NMR Spectroscopy

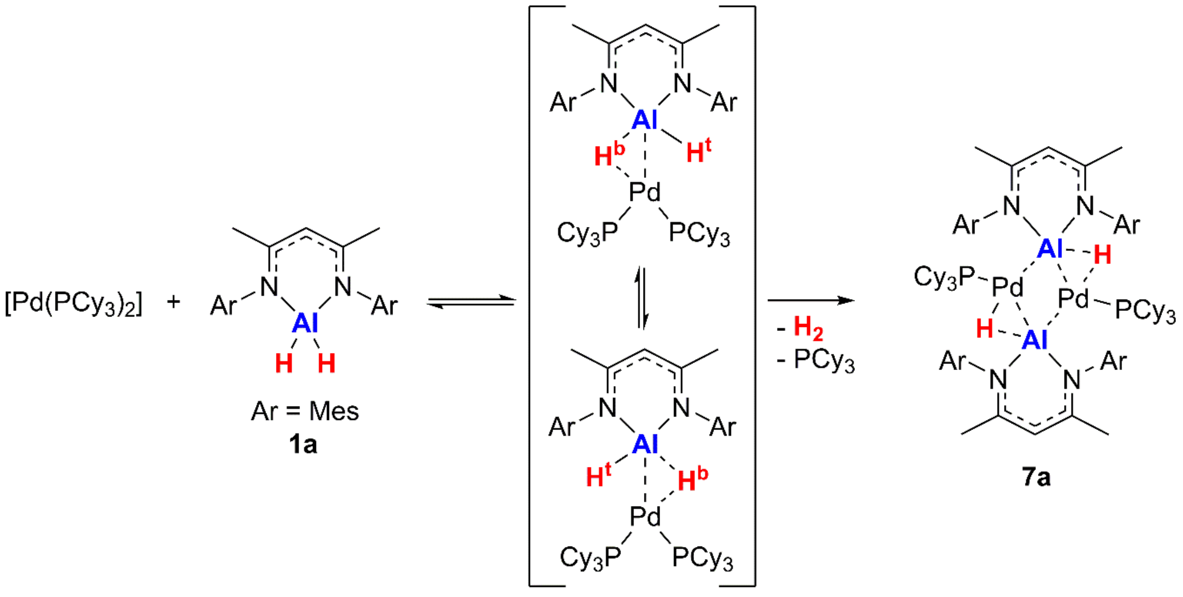

Figure S5.10. Fluxional processes observed upon mixing of **1a** with [Pd(PCy$_3$)$_2$].

In situ observation of transient Pd–H–Al σ-complex: In a glovebox, [Pd(PCy$_3$)$_2$] (18.4 mg, 0.0275 mmol, 1 equiv.) and Mes-*BDI*AlH$_2$ (**1a**, 10 mg, 0.0275 mmol, 1 equiv.) were weighed into separate 4 mL scintillation vials, dissolved in toluene-D$_8$ (0.3 mL each) and cooled to –35 °C. Both solutions were added to a pre-cooled Young's tap NMR tube upon which a colour change to dark orange was observed. The Young's tap NMR tube was quickly removed from the glovebox and stored at –198 °C. The reaction was monitored using variable temperature NMR spectroscopy between –80 and 100 °C

In the low temperature regime (193 K) two hydride resonances can be observed at δ = –0.62 ppm (H$_b$) and 4.64 ppm (H$_t$). Upon warming both resonances disappear likely due to a shift to the high temperature regime in which the equilibrium shifts back toward the starting materials. ^{31}P-^{1}H coupling results in the observed doublet for H$_b$ where $^{2}J_{P\text{–}H}$ = 85 Hz. ^{1}H{^{31}P}NMR confirms coupling due to the presence of a singlet in ^{1}H spectrum (figure S5.12). Hydride resonances are further verified through repeat reaction using Mes-*BDI*AlD$_2$ and resonances are no longer present (figure S5.14).

In the ^{31}P{^{1}H} VT data, in the low temperature regime (193 K) a broadened peak assigned to the proton coupled phosphine environment of **4** can be observed at δ = 37.0 ppm alongside

[Pd(PCy3)3] at δ = 23.5 ppm and free PCy3 at δ = 8.4 ppm. Comparison of the ^{31}P and $^{31}P\{^1H\}$ NMR spectra confirms the $^2J_{P-H}$ coupling and supports the assignment (Figure 5.13). The additional resonances have not been assigned. Upon warming **4** is consumed and the spectra is reminiscent of previously reported spectra for the room temperature reaction of [Pd(PCy3)2] with **1a** which forms **7a** as an insoluble red precipitate.

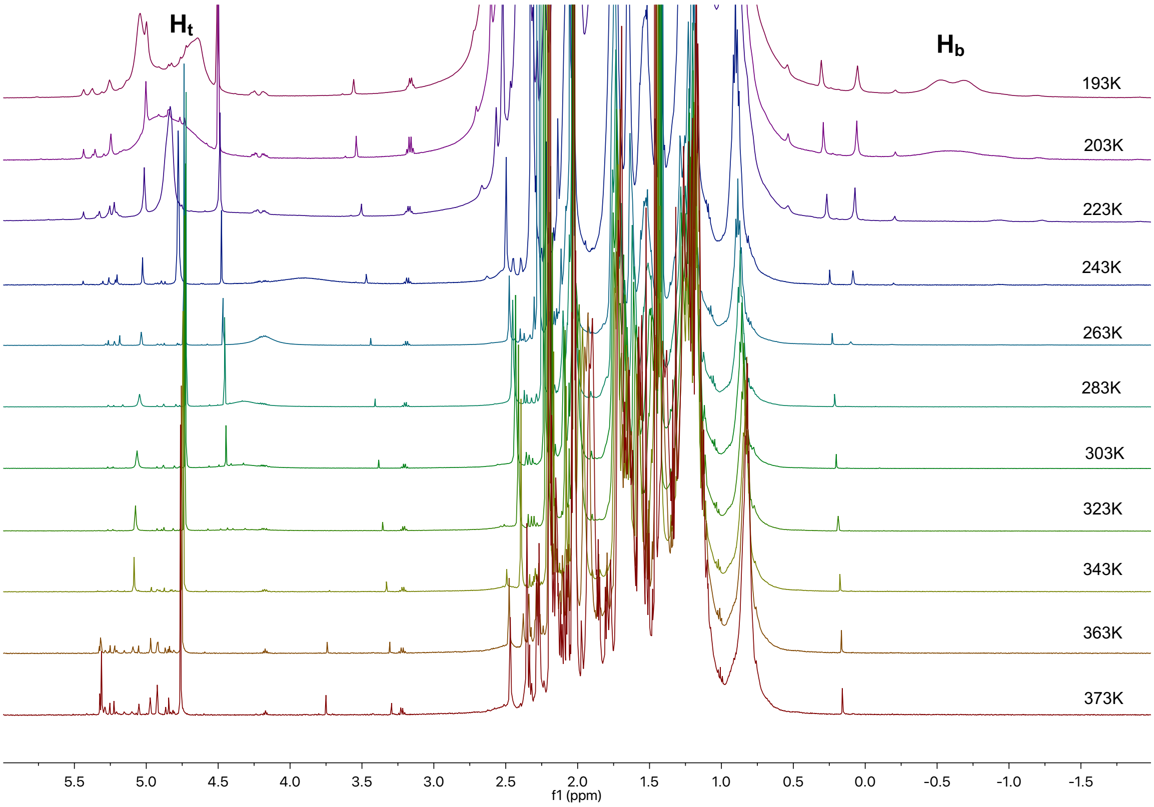

Figure S5.11. Variable temperature ^1H NMR of crude reaction mixture from –80 to +100 °C (193 K to 373 K) in toluene-d^8. Data presented from +6.0 to -2.0 ppm.

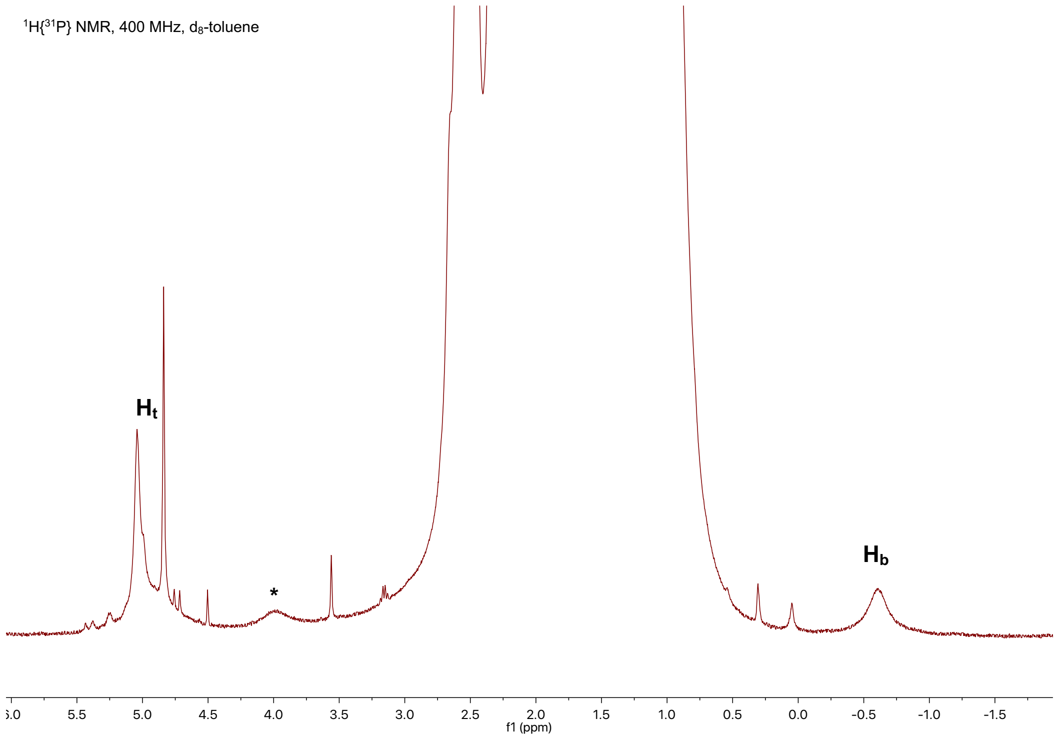

Figure S5.12. $^1H\{^{31}P\}$NMR spectrum at 193 K of crude reaction mixture. * Denotes unidentified impurity.

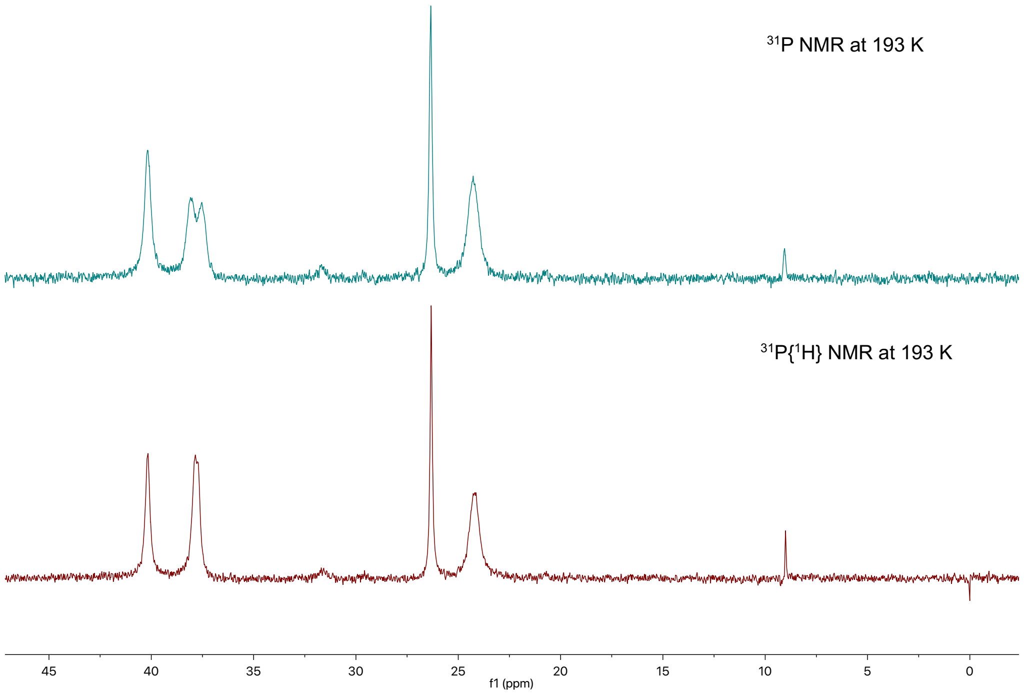

Figure S5.13: Stack plot comparing proton-coupled and proton-decoupled ^{31}P NMR spectra at 193K.

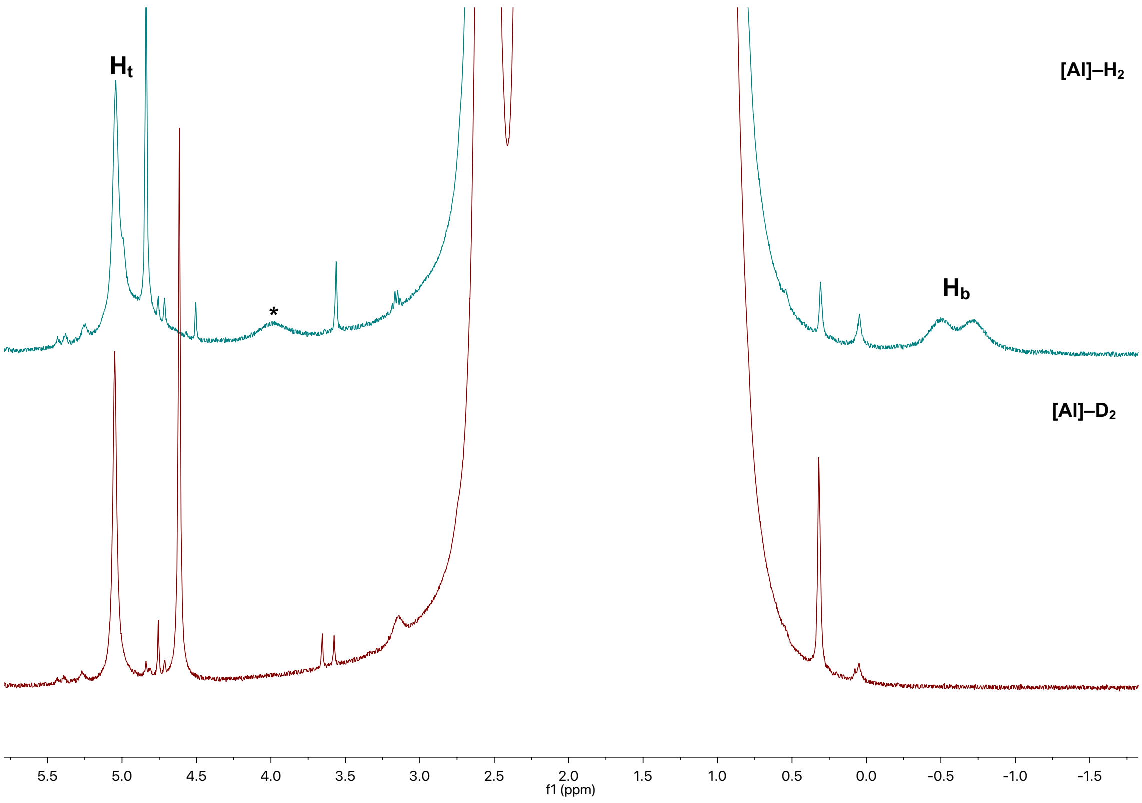

Figure S5.14: Comparison of ^{1}H NMR spectra for non-deuterated and deuterated analogues. * Denotes unidentified impurity.

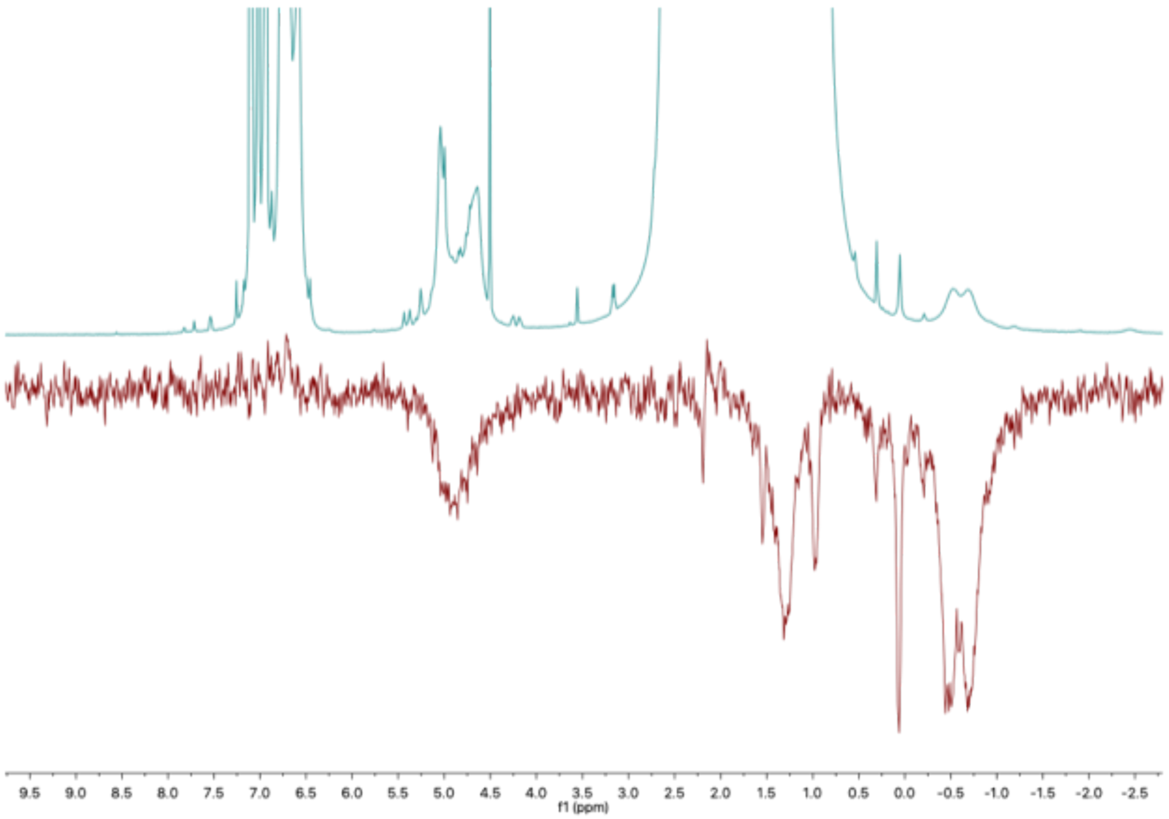

Figure S5.15: (blue: ^{1}H NMR at 193 K; red: ROESY) ROESY experiment on crude mixture at -80 °C (193 K) in toluene-D_8: Selective excitation *via* a soft pulse of the resonance at –0.61 ppm results in signal enhancement and confirms chemical exchange with both bridging and terminal hydrides at -0.61 and 4.80 ppm respectively. The outstandingly attenuated envelope of signals between 2.3 and 0 ppm are due to the imperfect selectivity of the soft pulse and does not represent a real process.

5.2.2 DFT calculations on the formation of 4

In order to gain further support to the formation of the σ-complex **4**, the thermodynamics of these complexations were explored by DFT calculations (for methods see section 7), and their formation is energetically favourable. The proposed three-coordinate complex **4** with two phosphine ligands is more stable than its two-coordinate analogue (figure S5.16), although dissociation of one PCy_3 is energetically feasible.

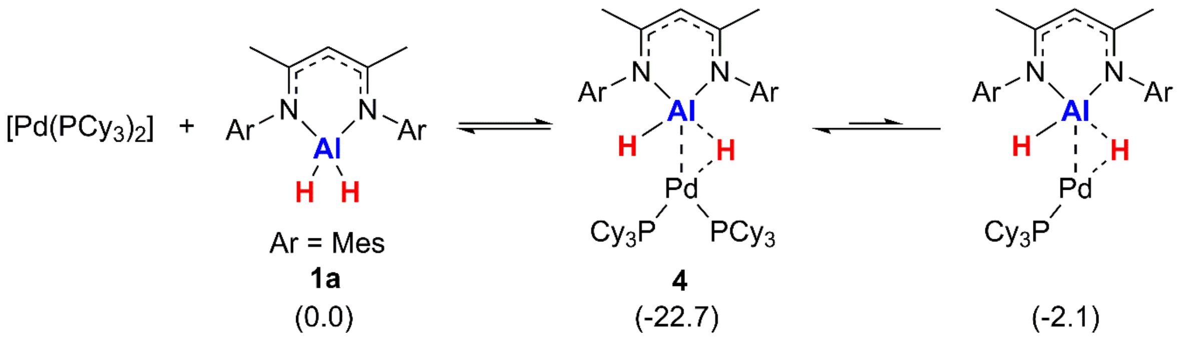

Figure S5.16: Coordination of **1a** to form **4** and possible formation of two-coordinate monophosphine complexes. Relative free energy in kcal mol^{-1} is included in parenthesis (M06L-D3).

The bonding of complex **4** was also inspected by NBO and QTAIM calculations in order to gain some more insight into the reversible coordination of the Al–H bond to Pd. As previously mentioned, the formulation of complex **4** as the mono-phosphine complex cannot be ruled out, although bonding analysis was performed on both complexes and the main bonding characteristics were very similar for both complexes. For this reason, only the results for the bisphosphine complex **4** will be presented herein.

	Opt
Pd	-0.03
Al	1.41
$H_{bridging}$	-0.36
$H_{terminal}$	-0.51
P_{cis}	0.91
P_{trans}	0.96

Table S5.1: Selected NPA charges for complex **4**. P_{cis} refers to the phosphorus atom closer to the bridging hydride (approximately *cis*) and P_{trans} to the phosphorus further away from the bridging hydride (approximately *trans*).

The NPA charges are very similar to complex **5** (*vide infra*), which supports our formulation of complexes **4** and **5** as σ-complexes of Pd(0) and Al(III).

	WBI
Pd-Al	0.28
Pd-H$_{bridging}$	0.20
Al-H$_{bridging}$	0.51
Al-H$_{terminal}$	0.65
Pd-P$_{cis}$	0.26
Pd-P$_{trans}$	0.42

Table S5.2: Selected Wiberg Bond Indices (WBI) for complex **4**.

In the WBI analysis it becomes apparent that the Al–H bond that coordinates to Pd is weakened and this is nicely illustrated in the Second Order Perturbation Theory Analysis. A significant donation from the bonding σ-(Al-H) orbital to an empty s orbital in Pd is present. Also, a non-negligible back-donation interaction from a d orbital in Pd to the anti-bonding σ*-(Al-H) can be found. Based on the Dewar-Chatt-Duncanson model, this complex is thus a textbook example of a σ-complex.

QTAIM is also analogous to that of complex **5** (*vide infra*).

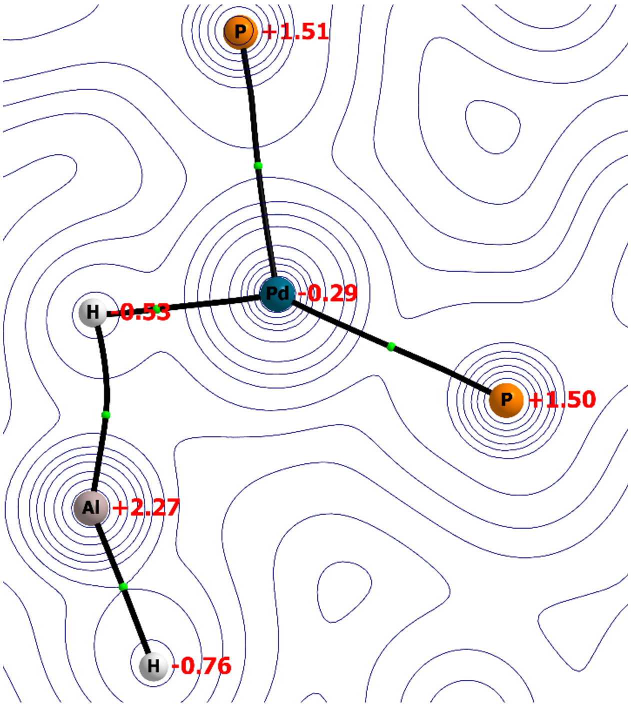

Figure S5.17: QTAIM contour plot of ρ for **4**. Charges are depicted in red.

5.3 Reaction of [Pd(PCy$_3$)$_2$] with Dep-*BDI*AlH$_2$ (1f)

5.3.1 Variable Temperature NMR Spectra of the Reaction of [Pd(PCy$_3$)$_2$] with Dep-BDIAlH2 (1f)

In situ observation of transient Pd–H–Al σ-complex: In a glovebox, [Pd(PCy$_3$)$_2$] (18.4 mg, 0.0275 mmol, 1 equiv.) and Dep-*BDI*AlH$_2$ (**1f**, 10.9 mg, 0.0275 mmol, 1 equiv.) were weighed into separate 4 mL scintillation vials, dissolved in toluene-D$_8$ (0.3 mL each). The solutions were mixed and transferred to a Young's tap NMR tube and the reaction was monitored using variable temperature NMR spectroscopy between –80 and 25 °C.

Cooling the solution to 193 K gave similar spectra to those observed for **4** (see above) with a broad upfield doublet observed at δ = –0.61 ppm ($^2J_{H–P}$ ≈ 85 Hz) in the ^{1}H NMR spectrum which resolves to a broad singlet in the ^{1}H{^{31}P} NMR spectrum (figure S5.17). The ^{31}P{^{1}H} NMR spectrum is also similar to that of **4** with a number of broad resonances (figure S5.18). Upon warming to 298 K, the ^{31}P{^{1}H} NMR spectrum resolves to a broad singlet at δ = 38.3 ppm close to the literature value for [Pd(PCy$_3$)$_2$] at δ = 39.1 ppm.

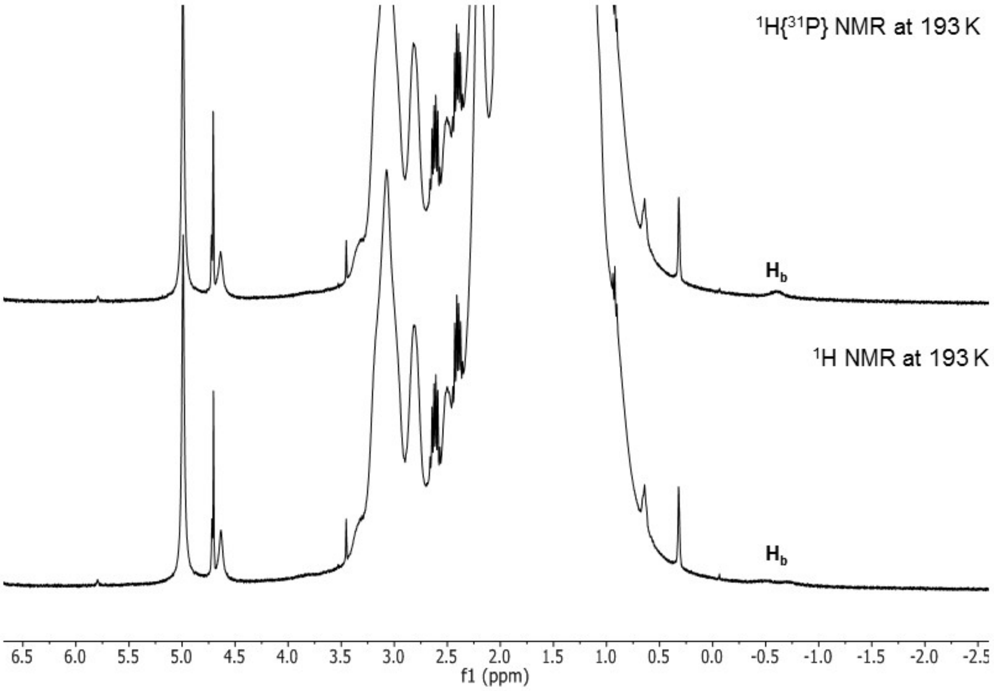

Figure S5.18. ^{1}H and ^{1}H{^{31}P} NMR spectra at 193 K of crude reaction mixture of **1f** with [Pd(PCy$_3$)$_2$].

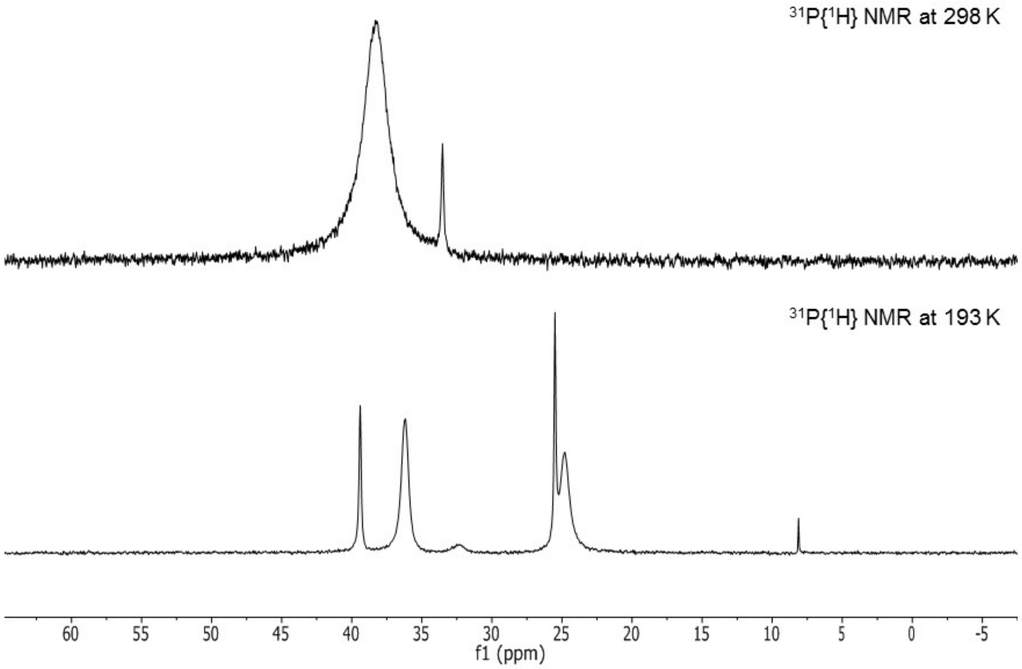

Figure S5.19. $^{31}P\{^1H\}$ NMR spectra at 193 and 298 K of crude reaction mixture of **1f** with $[Pd(PCy_3)_2]$.

6. Solid State NMR Spectroscopy Data

SS MAS ^{27}Al NMR spectra were acquired at a static magnetic field strength of 9.4 T in a 2.5 mm MAS probe tuned to 104.27 MHz and referenced to $[Al(NO_3)_3]$ using yttrium aluminium garnet at 0.0 ppm as a secondary reference. Samples were packed under argon into zirconia MAS rotors with Vespel caps and spun at 30,000 Hz using dry nitrogen. A 30°-60° Hahn-echo pulse sequence was used with one rotor period delay and no ^{1}H decoupling.

First-principles calculations were performed using CASTEP version 8.0.[10] Calculations employed the Perdew-Burke-Ernzehorf (PBE) functional with the Tkatchenkoand Scheffer (TS) dispersion correction, and on-the-fly generated ultrasoft pseudopotentials. Geometry optimisation calculations used a plane-wave cut-off energy of 600 eV with a Monkhurst-Pack grid with a minimum k-point sampling space of 0.05 $Å^{-1}$. All atoms were allowed to relax and the unit cell parameters were fixed. NMR shielding values were calculated using the GIPAW method for the optimised structures,[11] with a plane-wave cut-off energy of 700 eV, and a Monkhurst pack grid with a minimum k-point spacing of 0.05 $Å^{-1}$. Conversion of the calculated chemical shielding values (σ_{iso}) outputted from a GIPAW NMR calculation to a scale comparable to experimental isotropic chemical shift values was achieved using $\delta_{iso} = -(\sigma_{iso} - \sigma_{ref})/m$, where δ_{iso} are calculated isotropic chemical shift values and σ_{ref} = -552.21 ppm and m = 0.94.[12]

[10] S.J. Clark, M.D. Segall, C.J. Pickard, P.J. Hasnip, M.I.J. Probert, K. Refson, M.C. Payne, *Z. Kristallogr.* 2005, **220**, 567.
[11] (a) C.J. Pickard, F. Mauri, *Phys. Rev. B,* 2001, **63**, 245101. (b) J.R. Yates, C.J. Pickard, F. Mauri, *Phys. Rev. B,* 2007, **76**, 024401.
[12] D.M. Dawson, J.M. Griffin, V.R. Seymour, P.S. Wheatley, M. Amri, T. Kurkiewicz, N. Guillou, S. Wimperis, R.I. Walton, S.E. Ashbrook, *J. Phys. Chem. C*, 2017, **121**, 1781.

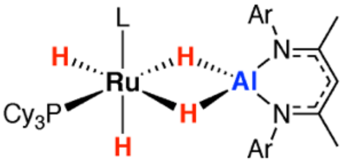

3, Ar = Dipp, L = N_2

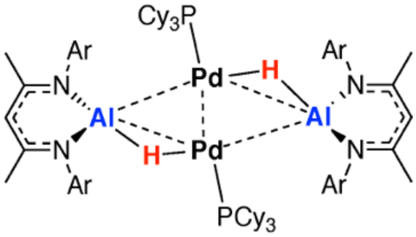

7‘ Ar = Mes

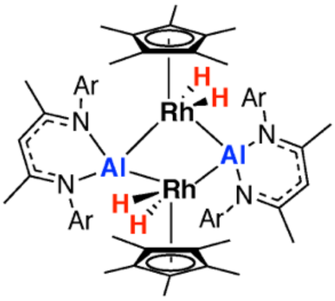

10, Ar = Mes

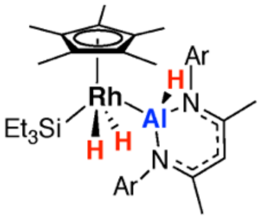

11, Ar = Mes

Figure S6.1: Heterometallic compounds selected for SS MAS ^{27}Al NMR spectroscopy.

Compound	***δ_{Al} (ppm)***	***C_Q (MHz)***
**3**	-	18.4
**7a**[a]	230	17
**10**	120	5.8
**11**	150	9.2

[a] – **7a** is the analogue of **7c** in which Ar = Mes.

Table S6.1: Isotropic chemical shift, δ_{Al} and C_Q values for heterometallic complexes

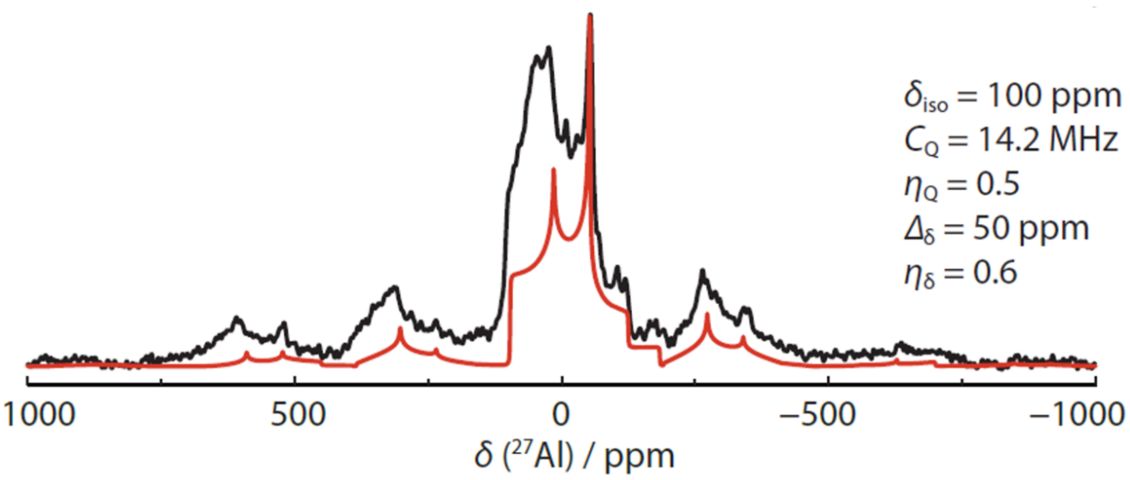

Figure S6.2: ^{27}Al solid-state MAS NMR spectrum of **1a** (black trace), overlaid with pNMRsim calculations from DFT parameters (red trace).

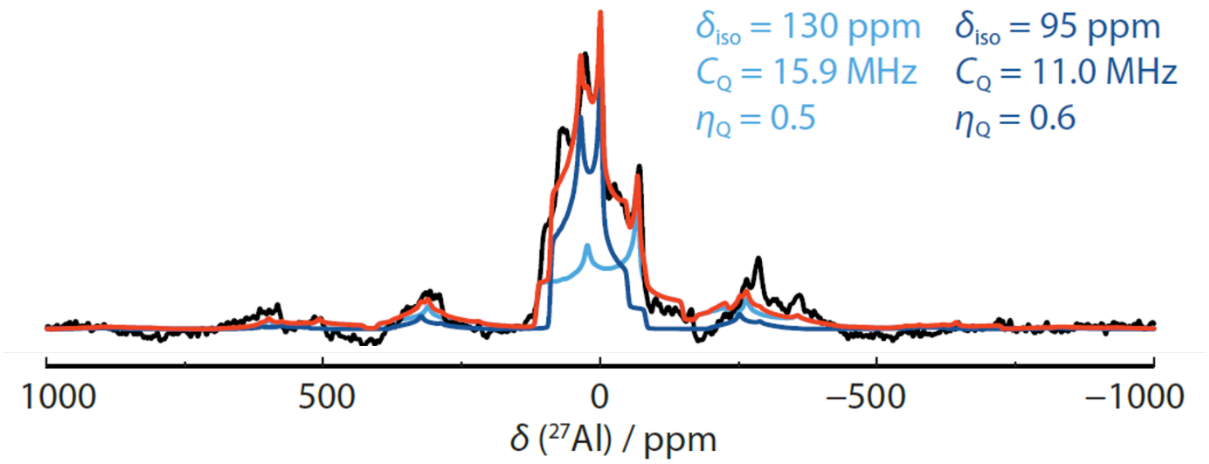

Figure S6.3: ^{27}Al solid-state MAS NMR spectrum of **1b** (black trace), overlaid with pNMRsim calculations from DFT parameters (red trace) and quadrupolar line shape fit (light blue trace). (Dark blue trace = impurity).

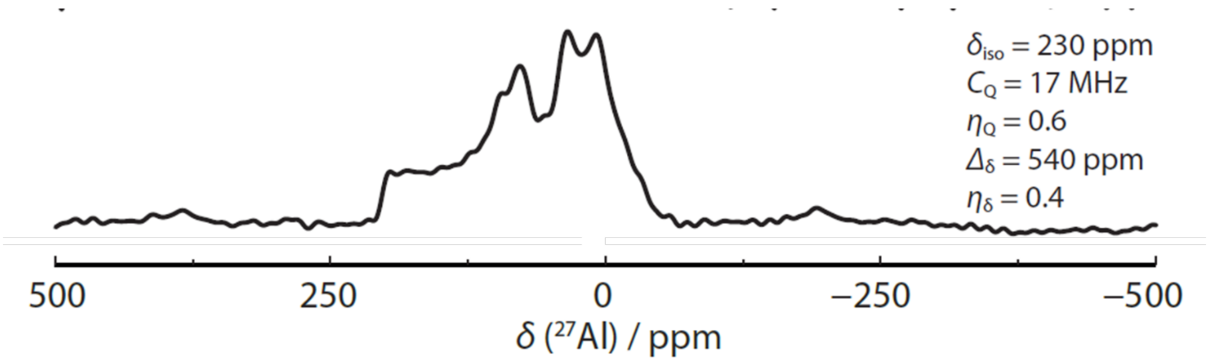

Figure S6.4: ^{27}Al solid-state MAS NMR spectrum of **7a**.

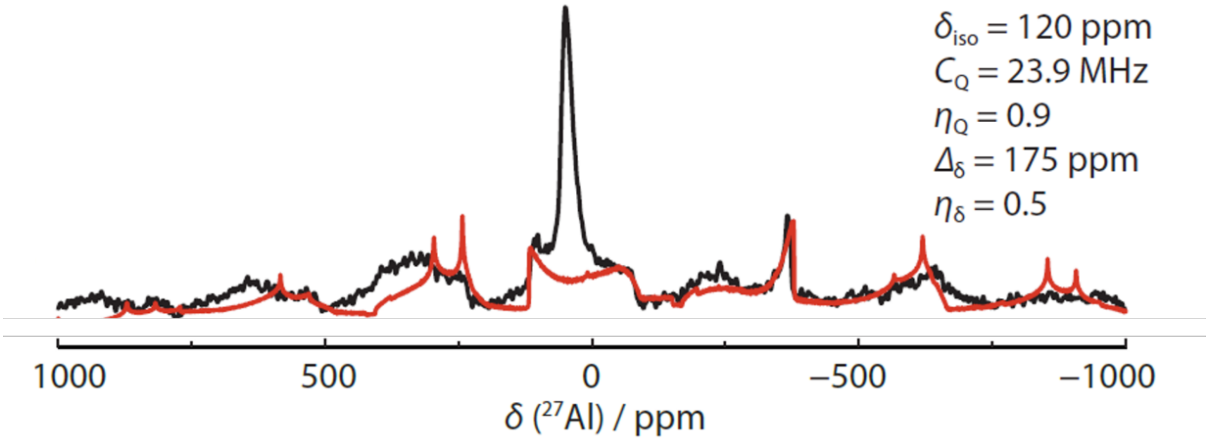

Figure S6.5: ^{27}Al solid-state MAS NMR spectrum of **8** (black trace), overlaid with pNMRsim calculations from DFT parameters (red trace).

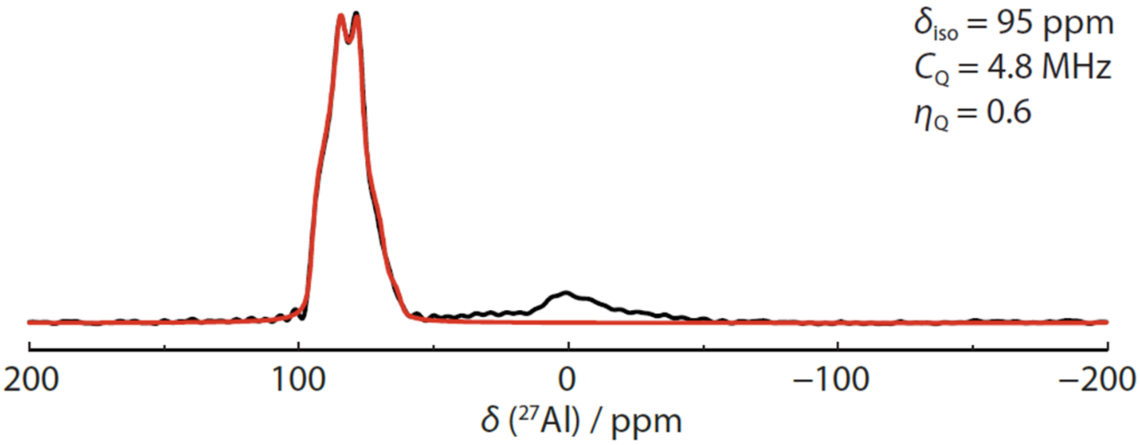

Figure S6.6: ^{27}Al solid-state MAS NMR spectrum of **9a** (black trace), overlaid with pNMRsim calculations from DFT parameters (red trace).

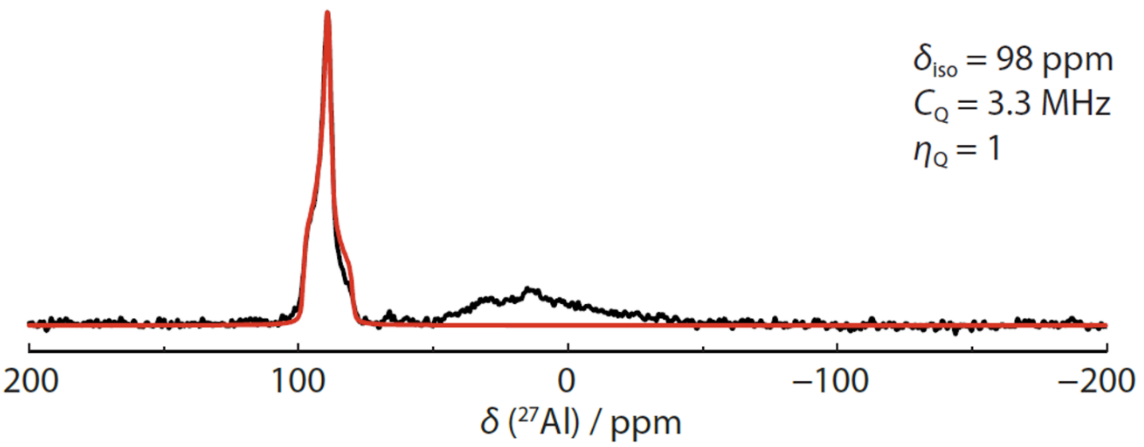

Figure S6.7: ^{27}Al solid-state MAS NMR spectrum of **9b** (black trace), overlaid with pNMRsim calculations from DFT parameters (red trace).

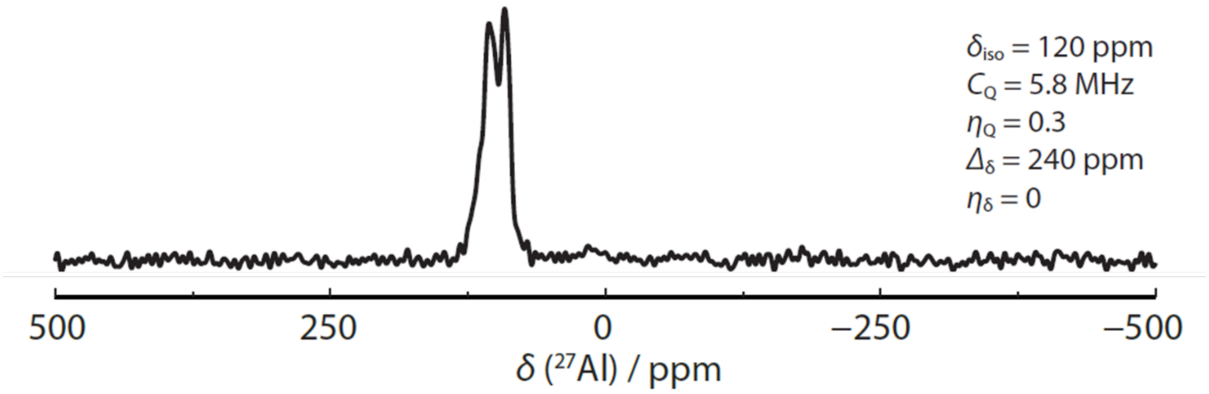

Figure S6.8: ^{27}Al solid-state MAS NMR spectrum of **10**.

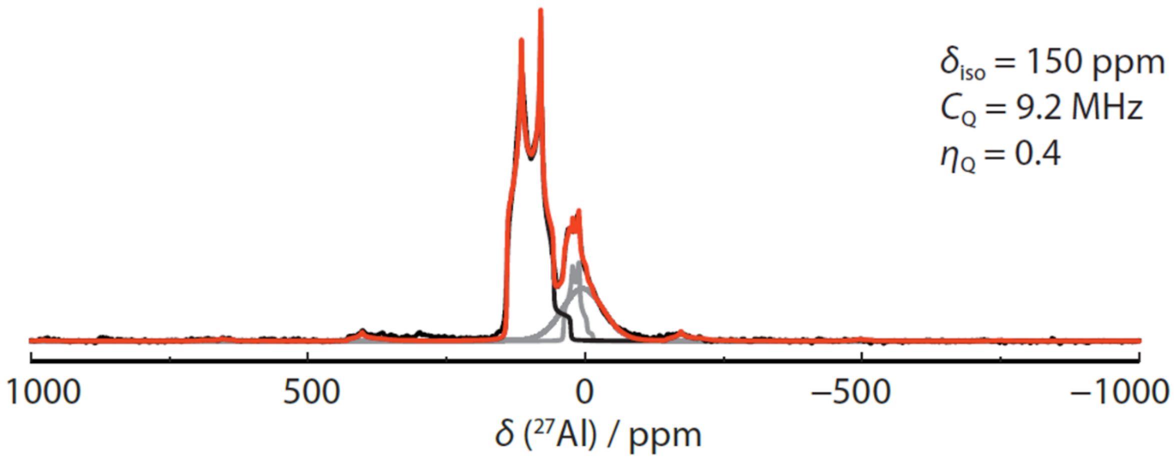

Figure S6.9: ^{27}Al solid-state MAS NMR spectrum of **11** (black trace), overlaid with pNMRsim calculations from DFT parameters (red trace).

7. Computational details

7.1 Methods

The geometries of products were optimised with the M06L Minnesota DFT functional using the Gaussian09 and Gaussian16 program packages.[13] Stationary points were characterised depending on their imaginary frequencies (0 for minima). NBO analysis was performed using the NBO 6.0 version program.[14] QTAIM analysis was conducted with the AIMAll package.[15] Non-covalent interactions were analysed with the NCIPLOT 3.0 program.[16]

Dispersion effects were included *via* single point energy corrections and were modelled using Grimme's D3 correction for M06L (EmpiricalDispersion=GD3).[17] The default numerical integration grid was also improved using a pruned grid with 99 radial shells and 590 angular points per shell (int=ultrafine). Solvent effects were not included since the reactions are carried out in rather nonpolar solvents. The level of theory employed in this study (M06L/BS1) was previously benchmarked by our group and it was shown to correlate accurately with experimental results.[18]

The basis set employed (BS1) was built as follows. The SDD effective core potential was used for all metals (SDDAll). The split-valence 6-31G* basis set was used for C and H atoms. The basis set for metal hydrides was expanded by adding one extra set of diffuse functions and three sets of p- and one set of d- polarisation functions, *i.e.* formally [6-31++G(d,3pd)]. The triple-ξ 6-311+G* basis set was used for heteroatoms.

---

[13] Frisch, M. J.; Trucks, G. W.; Schlegel, H. B.; Scuseria, G. E.; Robb, M. A.; Cheeseman, J. R.; Scalmani, G.; Barone, V.; Mennucci, B.; Petersson, G. A.; Nakatsuji, H.; Caricato, M.; Li, X.; Hratchian, H. P.; Izmaylov, A. F.; Bloino, J.; Zheng, G.; Sonnenberg, J. L.; Hada, M.; Ehara, M.; Toyota, K.; Fukuda, R.; Hasegawa, J.; Ishida, M.; Nakajima, T.; Honda, Y.; Kitao, O.; Nakai, H.; Vreven, T.; Montgomery, J. A., Jr.; Peralta, J. E.; Ogliaro, F.; Bearpark, M.; Heyd, J. J.; Brothers, E.; Kudin, K. N.; Staroverov, V. N.; Kobayashi, R.; Normand, J.; Raghavachari, K.; Rendell, A.; Burant, J. C.; Iyengar, S. S.; Tomasi, J.; Cossi, M.; Rega, N.; Millam, J. M.; Klene, M.; Knox, J. E.; Cross, J. B.; Bakken, V.; Adamo, C.; Jaramillo, J.; Gomperts, R.; Stratmann, R. E.; Yazyev, O.; Austin, A. J.; Cammi, R.; Pomelli, C.; Ochterski, J. W.; Martin, R. L.; Morokuma, K.; Zakrzewski, V. G.; Voth, G. A.; Salvador, P.; Dannenberg, J. J.; Dapprich, S.; Daniels, A. D.; Farkas, Ö.; Foresman, J. B.; Ortiz, J. V.; Cioslowski, J.; Fox, D. J. *Gaussian 09, Revision D.01*; Gaussian, Inc., Wallingford, CT, 2009.

[14] *NBO 6.0*. Glendening, E. D.; Badenhoop, J. K.; Reed, A. E.; Carpenter, J. E.; Bohmann, J. A.; Morales, C. M.; Landis, C. R.; Weinhold, F. Theoretical Chemistry Institute, University of Wisconsin, Madison (2013).

[15] AIMAll (Version 13.10.19), T. A. Keith, TK Gristmill Software, Overland Park KS, USA, 2013 (aim.tkgristmill.com).

[16] Johnson, E. R.; Keinan, S.; Mori-Sánchez, P.; Contreras-García, J.; Cohen, A. J.; Yang, W. *J. Am. Chem. Soc.* **2010**, *132*, 6498–6506.

[17] Grimme, S.; Antony, J.; Ehrlich, S.; Krieg, H. *J Chem. Phys.* **2010**, *132*, 154104.

[18] T. N. Hooper, M. Garçon, A. J. P. White and M. R. Crimmin, *Chem. Sci.*, **2018**, *9*, 5435–5440.

7.2 Bonding analysis

NBO analysis was performed on the optimised structures of a series of intermetallic complexes.

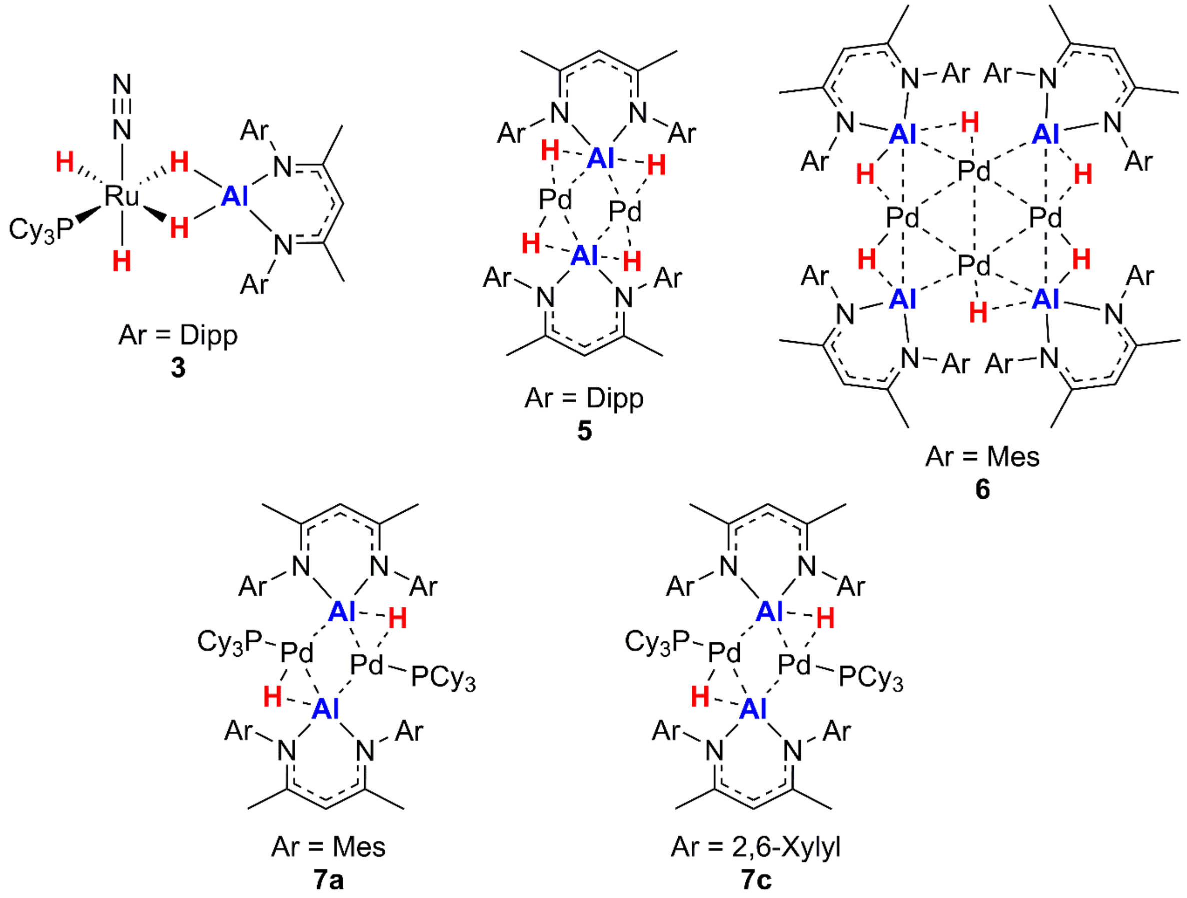

Figure S7.1: Intermetallic complexes analysed by NBO calculations.

For the octametallic complex **6**, an optimised structure could not be obtained, due to unsurmountable convergence problems. This complex presented severe difficulties to converge the SCF equations. These problems could only be circumvented by using a quadratically convergent SCF procedure (scf=qc, or scf=xqc in G16). This approach increased dramatically the computational cost, making an optimisation inviable and hence only a single point calculation could be obtained. For this reason, the NBO analysis for **6** was performed using the geometry obtained from X-ray diffraction. In order to validate this approximation, the NBO analysis for the rest of complexes was performed on both the experimental and optimised geometries. The differences were small and qualitatively irrelevant.

7.3 NPA charges (NBO)

A summary of the relevant NPA charges for the different complexes is presented below.

	Single point	*Opt*
Ru	-0.76	-0.64
Al	1.70	1.63
P	1.17	1.28
H_t	-0.06	-0.12
H_b	-0.20	-0.22
H_b	-0.34	-0.34
H_t	-0.01	-0.00

Table S7.1: Selected NPA charges for complex **3**. H_t refers to the terminal hydrides, H_b to the bridging hydrides.

	Single point	*Opt*
Pd	-0.09	-0.05
Al	1.41	1.40
H	-0.37	-0.36
H	-0.31	-0.33

Table S7.2: Selected NPA charges for complex **5**.

	Single point
$Pd_{(2H)}$	-0.11
H	-0.36
H	-0.35
$Pd_{(1H)}$	-0.04
H	-0.17
Al	1.16
Al	1.18

Table S7.3: Selected NPA charges for complex **6**. $Pd_{(2H)}$ refers to the Pd atoms with two hydrides whereas $Pd_{(1H)}$ refers to the Pd atoms with one hydride.

	Single point	*Opt*
Pd	-0.16	-0.14
Al	1.17	1.18
H	-0.46	-0.47
P	0.92	0.98

Table S7.4: Selected NPA charges for complex **7a**.

	Single point	*Opt*
Pd	-0.16	-0.14
Al	1.16	1.18
H	-0.46	-0.47
P	0.93	0.98

Table S7.5: Selected NPA charges for complex **7c**.

	Opt
Pd	-0.14
Al	1.16
H	-0.45
P	0.98

Table S7.6: Selected NPA charges for complex **7e**. Note this complex could not be experimentally isolated and a decomposition product was obtained instead (*vide infra*).

In order to rule out significant effects when using different ligands on the aluminium moieties, the same analysis was performed for the parent aluminium dihydrides and the corresponding Al(I) complexes.

Ar =	**1**	**8**
Mes (**a**)	1.49	0.75
Dipp (**b**)	1.50	0.80
2,6-xyl (**c**)	1.48	0.76
Ph (**e**)	1.45	0.71

Table S7.7: NPA charges on the Al centre for complexes **1a, 1b, 1c, 1e, 8, 8b, 8c, 8e.**

As expected, the charges for the intermetallic complexes are inside the range comprised by the parent Al(III) and Al(I) complexes. This is consistent with the proposed picture of these complexes as snapshots of the dehydrogenation process from Al(III) to Al(I). The effect of reducing the sterics of the β-diketiminate ligands was found to be very small, although it appears that the charges are marginally reduced when the ligand becomes smaller.

7.4 Metal–Metal interactions

	Pd-Al	Pd-H	Pd-Pd	Al-H
5	0.27, 0.30	0.23-0.26	0.04	0.49-0.50
**7a**	0.27, 0.41	0.11	0.10	0.49
7c	0.27, 0.41[a]	0.10	0.09	0.49

[a] The highest WBI (*i.e.* 0.41) corresponds to the Pd-Al bond without bridging hydride.

Table S7.8: Selected Wiberg Bond Indices (WBI) for complexes **5, 7a, 7c**.

7.5 Non-Covalent Interactions (NCI) analysis

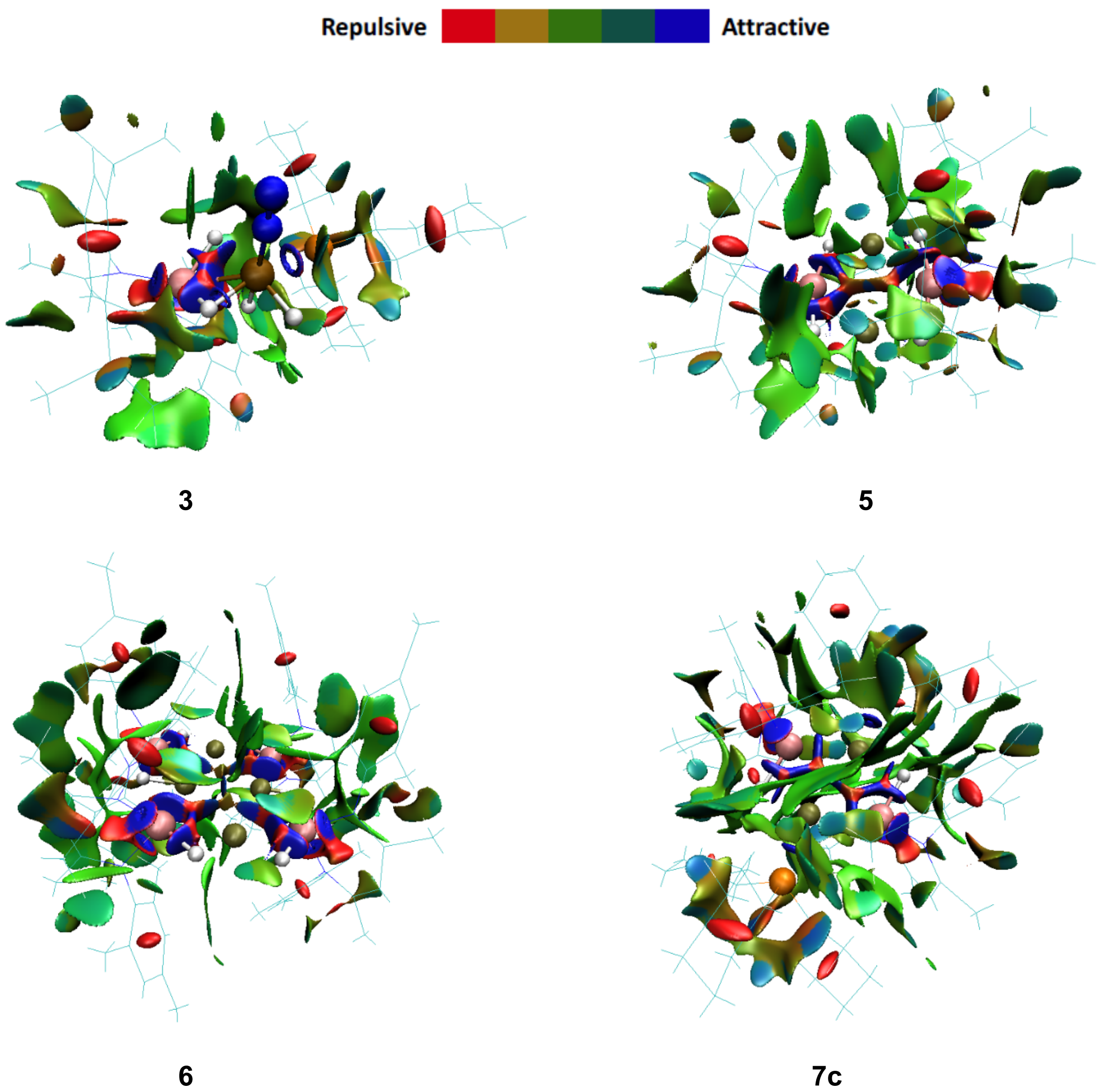

Figure S7.2: Non-covalent interaction (NCI) plots of complexes **3, 5, 6, 7c**.

7.6 QTAIM

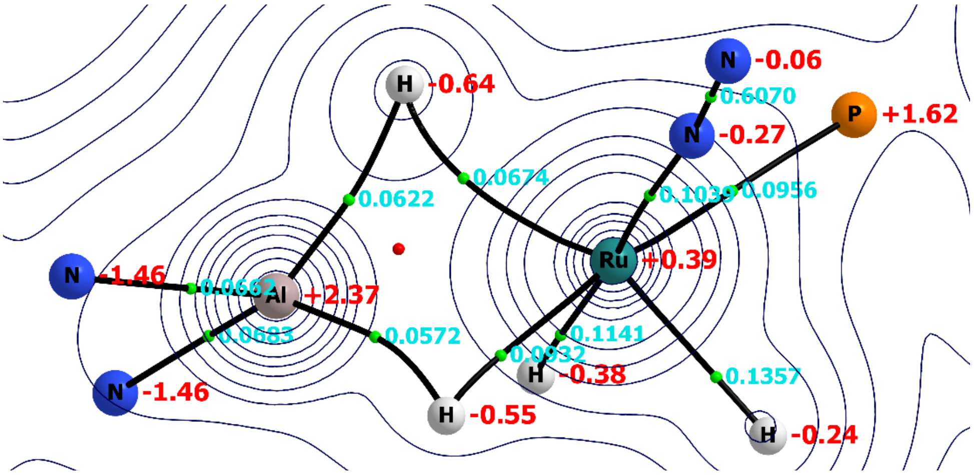

Figure S7.3: QTAIM contour plot of ρ for **3**. Charges are depicted in red and the electron density (ρ) in blue.

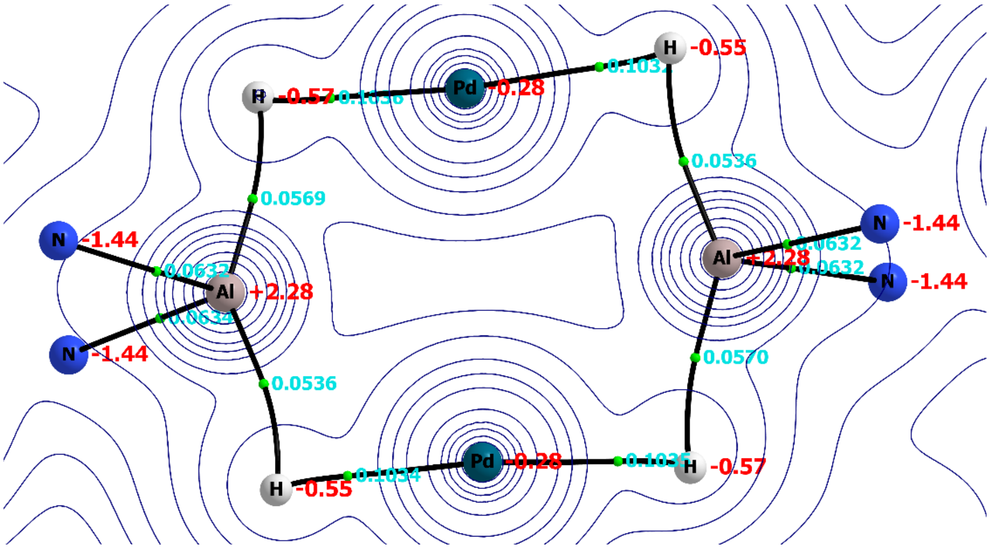

Figure S7.4: QTAIM contour plot of ρ for **5**. Charges are depicted in red and the electron density (ρ) in blue.

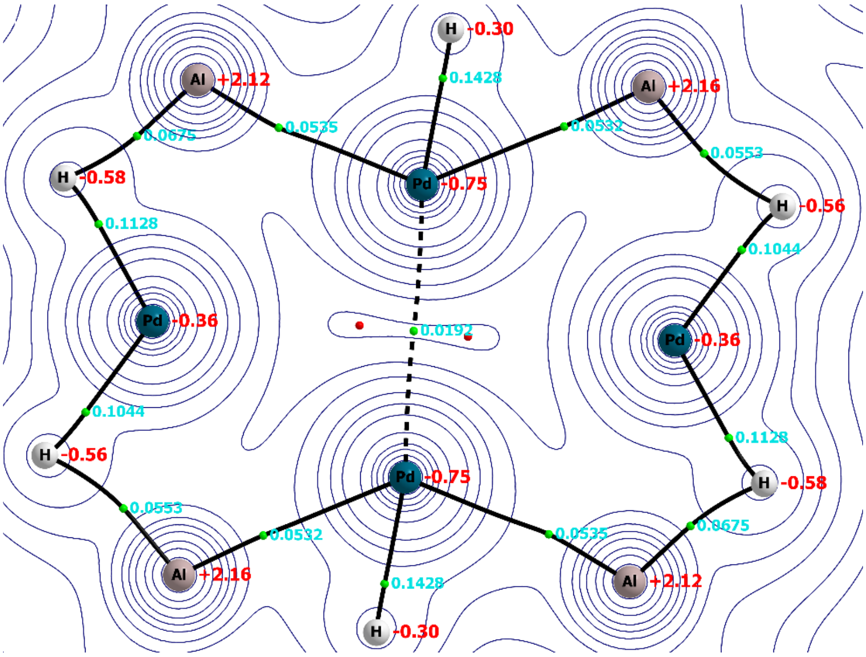

Figure S7.5: QTAIM contour plot of ρ for **6**. Charges are depicted in red and the electron density (ρ) in blue.

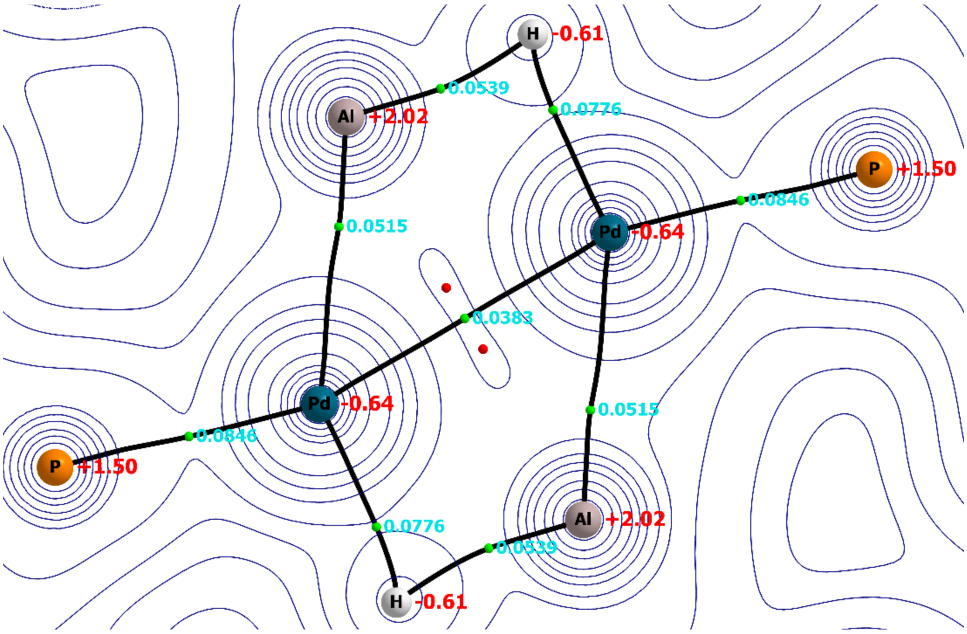

Figure S7.6: QTAIM contour plot of ρ for **7a**. Charges are depicted in red and the electron density (ρ) in blue.

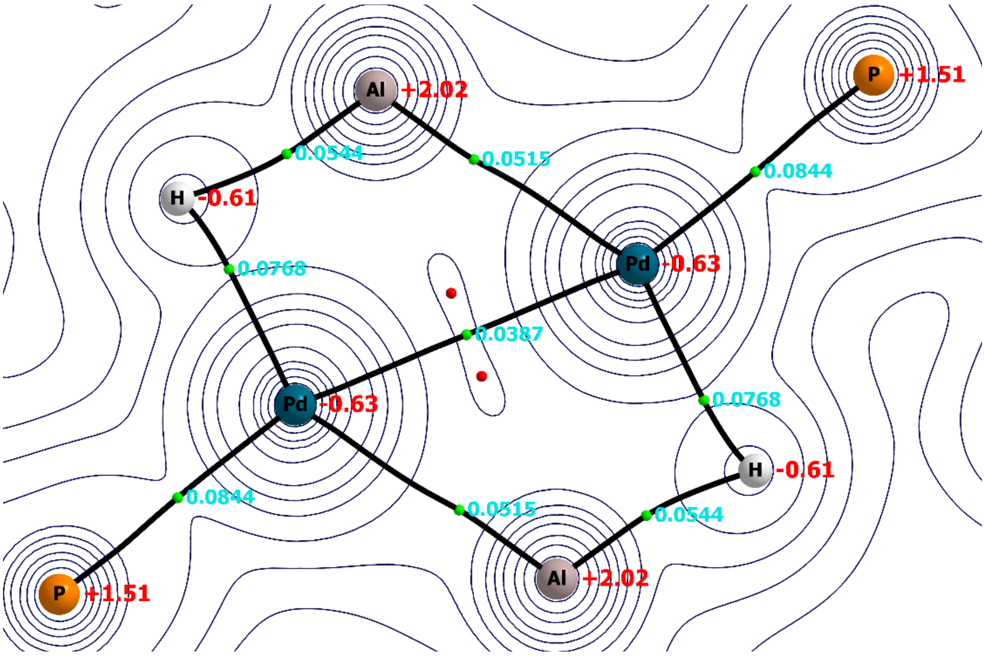

Figure S7.7: QTAIM contour plot of ρ for **7c**. Charges are depicted in red and the electron density (ρ) in blue.

7.7 XYZ coordinates

5.log

SCF (wB97x) = -2740.96521231
E(SCF)+ZPE(0 K)= -2739.653245
H(298 K)= -2739.575587
G(298 K)= -2739.764913
Lowest Frequency = 12.5476cm^{-1}

Pd	-0.626743	-1.158739	0.839997
Al	1.720273	-0.248640	1.019968
H	-2.171272	-0.868500	0.205976
H	0.796257	-1.618446	1.646880
N	3.343215	-1.237937	0.648130
N	2.364343	0.509645	2.680266
C	4.241915	-1.544723	1.584772
C	4.198753	-1.032814	2.892561
H	4.970493	-1.388238	3.568501
C	3.373203	-0.008958	3.382007
C	5.376290	-2.464885	1.241293
H	5.003670	-3.467950	0.999671
H	6.086774	-2.549581	2.067212
H	5.910008	-2.120011	0.347603
C	3.671803	0.532468	4.749081
H	3.837212	1.615889	4.723779
H	4.550513	0.053116	5.187631
H	2.817030	0.374942	5.418851
C	3.518303	-1.738974	-0.685584
C	4.131687	-0.911833	-1.649771
C	4.328112	-1.428094	-2.932543
H	4.818877	-0.807834	-3.683327
C	3.892628	-2.702518	-3.272841
H	4.045879	-3.081720	-4.282645
C	3.240491	-3.480879	-2.326491
H	2.869594	-4.469262	-2.601474
C	3.049190	-3.026347	-1.018686
C	4.598394	0.496666	-1.333290
H	4.221433	0.761357	-0.335720
C	4.021136	1.508209	-2.320702
H	4.342701	1.303799	-3.350882
H	4.345328	2.525828	-2.069540
H	2.920173	1.491133	-2.302170
C	6.123050	0.589043	-1.287830
H	6.552220	-0.089291	-0.539504
H	6.442273	1.608731	-1.034255
H	6.567493	0.333601	-2.259395
C	2.336073	-3.920906	-0.022898
H	2.334822	-3.418252	0.954637
C	3.028706	-5.275367	0.134911
H	2.996431	-5.847913	-0.801522
H	2.526198	-5.877133	0.902032
H	4.083811	-5.182652	0.420613
C	0.882141	-4.138826	-0.437601
H	0.352440	-3.186208	-0.593877
H	0.337870	-4.702203	0.332413
H	0.823094	-4.706590	-1.375287

C	1.738011	1.719660	3.129906
C	0.581121	1.667456	3.931193
C	-0.004737	2.874635	4.324732
H	-0.906110	2.848011	4.938534
C	0.540164	4.096080	3.954025
H	0.074148	5.025334	4.280198
C	1.674995	4.128210	3.152645
H	2.093588	5.088248	2.847741
C	2.283759	2.952466	2.710001
C	-0.035954	0.361050	4.390048
H	0.553049	-0.464112	3.963916
C	-1.471049	0.218638	3.887559
H	-2.127828	0.976838	4.335225
H	-1.875125	-0.769080	4.147133
H	-1.533881	0.330946	2.794077
C	-0.014101	0.233585	5.914147
H	0.995744	0.326859	6.332204
H	-0.418270	-0.737018	6.227130
H	-0.631011	1.012856	6.381659
C	3.513031	3.039444	1.823542
H	3.767339	2.022081	1.493094
C	4.715504	3.594340	2.587803
H	4.518983	4.613262	2.947985
H	5.601117	3.636395	1.940939
H	4.970378	2.980004	3.460059
C	3.251162	3.873083	0.570864
H	2.434533	3.446761	-0.027795
H	4.150832	3.909288	-0.058015
H	2.982739	4.908674	0.820529
Pd	0.641434	1.130258	-0.865830
Al	-1.709368	0.228373	-1.035902
H	2.041353	1.161731	0.088429
H	-0.658116	1.318533	-1.945755
N	-3.238052	1.392554	-0.792841
N	-2.448101	-0.697297	-2.568080
C	-4.119599	1.656082	-1.758381
C	-4.149470	0.960998	-2.977354
H	-4.899799	1.283750	-3.692657
C	-3.429401	-0.195655	-3.319798
C	-5.151114	2.723625	-1.540031
H	-4.668576	3.700049	-1.405810
H	-5.843525	2.791517	-2.382791
H	-5.726420	2.544483	-0.624238
C	-3.814051	-0.897100	-4.589097
H	-4.020351	-1.958873	-4.408512
H	-4.693197	-0.438867	-5.048654
H	-2.990168	-0.870010	-5.312975
C	-3.429868	1.952867	0.513724
C	-4.213012	1.232975	1.442351
C	-4.399545	1.780782	2.712793
H	-5.013331	1.243085	3.436590
C	-3.804469	2.983328	3.074638
H	-3.954133	3.388777	4.074764
C	-3.008153	3.658487	2.160104
H	-2.526158	4.593500	2.448895
C	-2.809731	3.166980	0.866194
C	-4.874868	-0.088577	1.096125
H	-4.510555	-0.402869	0.107439
C	-4.503637	-1.188713	2.088116

H	-4.832423	-0.945871	3.107802
H	-4.984206	-2.134812	1.804991
H	-3.417728	-1.353110	2.112434
C	-6.394578	0.055364	1.007179
H	-6.696241	0.793997	0.254474
H	-6.860664	-0.901393	0.739117
H	-6.818459	0.372890	1.969512
C	-1.953920	3.958097	-0.103216
H	-1.913883	3.406128	-1.053540
C	-2.543951	5.343081	-0.373924
H	-2.555388	5.950732	0.540928
H	-1.943056	5.881020	-1.117500
H	-3.575395	5.298231	-0.744668
C	-0.523841	4.105240	0.412187
H	-0.069289	3.128823	0.642562
H	0.107841	4.600657	-0.337374
H	-0.490624	4.708460	1.329541
C	-1.829536	-1.933758	-2.953885
C	-0.775704	-1.929546	-3.890747
C	-0.201507	-3.153732	-4.244621
H	0.622935	-3.162899	-4.958958
C	-0.662907	-4.348654	-3.709854
H	-0.212282	-5.293856	-4.010742
C	-1.687102	-4.331349	-2.771531
H	-2.033172	-5.268415	-2.334010
C	-2.273065	-3.132996	-2.357933
C	-0.236270	-0.654888	-4.510894
H	-0.855926	0.186084	-4.168516
C	1.197115	-0.396097	-4.050712
H	1.874603	-1.177429	-4.418929
H	1.559030	0.569999	-4.427944
H	1.278203	-0.379102	-2.952608
C	-0.282015	-0.692464	-6.039200
H	-1.286587	-0.897838	-6.429389
H	0.050131	0.265634	-6.457257
H	0.384216	-1.469452	-6.436817
C	-3.370104	-3.162373	-1.310366
H	-3.588245	-2.125254	-1.020382
C	-4.660859	-3.766057	-1.862165
H	-4.512601	-4.809090	-2.173372
H	-5.450558	-3.756185	-1.099123
H	-5.032663	-3.211047	-2.732856
C	-2.917914	-3.900670	-0.052622
H	-2.027039	-3.422882	0.384594
H	-3.708804	-3.894647	0.707757
H	-2.667902	-4.949713	-0.261059

1b-DippAlH2.log

SCF (wB97x) = -1242.43254983
E(SCF)+ZPE(0 K)= -1241.778080
H(298 K)= -1241.741162
G(298 K)= -1241.844398
Lowest Frequency = 20.2417cm^{-1}

H	-2.978149	2.695685	2.163096
C	-2.701573	3.288391	1.282279
H	-3.608221	3.801736	0.935287
H	-1.991294	4.059018	1.607307

C	-2.096345	2.424411	0.176441
H	-1.179879	1.964400	0.573691
C	-1.695581	3.287089	-1.018848
H	-1.194086	2.691601	-1.791538
H	-1.011457	4.086412	-0.704648
C	1.275617	-0.430008	1.712875
C	2.482834	-0.378247	2.603584
H	3.112383	-1.264569	2.450878
H	2.199473	-0.340464	3.658512
H	3.113707	0.488041	2.372652
H	1.611395	2.186400	1.851022
H	1.151399	3.786740	1.257602
H	2.866024	3.376552	1.445264
C	1.916751	2.478230	-0.300495
C	2.071511	3.650108	-1.262276
H	2.123011	3.314181	-2.304841
H	2.972469	4.241259	-1.051776
C	4.216522	1.707952	-1.048250
H	4.438773	2.735018	-1.331197
C	5.178862	0.724119	-1.246380
H	6.145783	0.985802	-1.672850
C	4.895575	-0.593065	-0.917148
H	5.640999	-1.367577	-1.099205
C	3.663355	-0.953988	-0.364387
H	5.422706	-3.118666	0.228704
C	4.468783	-3.067143	0.769338
H	4.190836	-4.094857	1.032327
C	2.712082	0.060550	-0.141669
C	2.967314	1.401390	-0.505459
H	0.937562	2.018383	-0.505024
H	2.428680	-2.487524	0.464275
C	3.379419	-2.416911	-0.082660
C	3.210385	-3.187538	-1.392982
H	2.403103	-2.769931	-2.004583
H	2.972643	-4.240676	-1.197534
H	4.135302	-3.156467	-1.984672
Al	0.009011	-0.639576	-0.882765
N	1.423587	-0.261762	0.397288
H	4.655035	-2.518390	1.701042
H	1.217352	4.330792	-1.170829
C	1.886930	2.978774	1.144912
C	0.027327	-0.634082	2.324642
H	0.039682	-0.775971	3.401046
C	-1.238000	-0.520187	1.728672
C	-2.439567	-0.511555	2.626719
H	-2.161755	-0.664349	3.672456
H	-3.152925	-1.291928	2.331829
H	-2.983130	0.438163	2.545233
H	-2.268003	-2.616755	0.105764
C	-3.233687	-2.534111	-0.412297
C	-3.065839	-3.150620	-1.800863
H	-2.299860	-2.627344	-2.384423
H	-2.767506	-4.203639	-1.726209
H	-4.006917	-3.108201	-2.365854
C	-4.271163	-3.314093	0.393848
H	-4.417756	-2.892912	1.396394
H	-3.964718	-4.360730	0.510254
H	-5.249086	-3.313965	-0.105249
C	-3.588145	-1.064186	-0.517280

C	-2.706604	-0.048709	-0.100706
C	-3.040711	1.313550	-0.241148
C	-4.284099	1.636655	-0.787936
H	-4.556937	2.686751	-0.899470
C	-5.171065	0.647943	-1.195591
H	-6.135124	0.919943	-1.621996
C	-4.818533	-0.688613	-1.063112
H	-5.509993	-1.464910	-1.392489
N	-1.412518	-0.386591	0.413066
H	-2.571153	3.766604	-1.476557
H	0.078235	-2.154205	-1.307254
H	-0.057357	0.476877	-1.985903

8-DippAl.log

SCF (wB97x) = -1241.19129130
E(SCF)+ZPE(0 K)= -1240.552170
H(298 K)= -1240.515924
G(298 K)= -1240.618036
Lowest Frequency = 22.8329cm^{-1}

H	-3.107272	2.800435	2.086654
C	-2.783369	3.354787	1.197317
H	-3.666499	3.862149	0.787268
H	-2.080956	4.132395	1.523111
C	-2.133410	2.439707	0.161000
H	-1.237477	2.000514	0.624293
C	-1.675014	3.242432	-1.055579
H	-1.145359	2.606571	-1.778845
H	-0.999133	4.054150	-0.754702
C	1.273205	-0.482576	1.697954
C	2.485475	-0.491916	2.587327
H	3.142597	-1.338841	2.351582
H	2.204916	-0.561146	3.641893
H	3.090666	0.411221	2.441188
H	1.529089	2.097927	1.860954
H	1.111934	3.725623	1.309552
H	2.811047	3.294069	1.578859
C	1.963827	2.479019	-0.253722
C	2.185625	3.687142	-1.154146
H	2.297030	3.395448	-2.205018
H	3.078166	4.256902	-0.864064
C	4.270206	1.684043	-0.952857
H	4.518052	2.713079	-1.205797
C	5.216886	0.686854	-1.155092
H	6.198200	0.939847	-1.552826
C	4.899123	-0.633867	-0.871000
H	5.633147	-1.416517	-1.061692
C	3.648642	-0.982436	-0.355201
H	5.311149	-3.305615	-0.062786
C	4.415998	-3.218206	0.565926
H	4.086360	-4.238180	0.797235
C	2.713851	0.044182	-0.121536
C	3.002522	1.387657	-0.447501
H	0.986419	2.046286	-0.537873
H	2.409925	-2.494815	0.500140
C	3.304298	-2.442862	-0.136084
C	2.948077	-3.099134	-1.470797
H	2.116606	-2.577404	-1.963505

H	2.659533	-4.148575	-1.329988
H	3.804907	-3.073703	-2.157103
Al	0.005294	-0.327860	-1.074746
N	1.409283	-0.264788	0.386163
H	4.722638	-2.740713	1.505473
H	1.333489	4.373180	-1.083171
C	1.848565	2.917323	1.206569
C	0.027138	-0.682046	2.306734
H	0.035215	-0.867561	3.376860
C	-1.229809	-0.555404	1.704557
C	-2.439168	-0.624174	2.592852
H	-2.163054	-0.806136	3.634981
H	-3.121148	-1.420445	2.267254
H	-3.017836	0.306606	2.541748
H	-2.305510	-2.602036	0.290201
C	-3.191475	-2.539893	-0.357783
C	-2.802815	-3.109725	-1.722021
H	-1.977411	-2.542117	-2.172359
H	-2.492035	-4.158833	-1.637561
H	-3.651274	-3.064836	-2.417898
C	-4.293503	-3.379781	0.283602
H	-4.610769	-2.972395	1.251963
H	-3.949052	-4.408225	0.446160
H	-5.184479	-3.435853	-0.354760
C	-3.575446	-1.079257	-0.485109
C	-2.701077	-0.046558	-0.095407
C	-3.057898	1.308120	-0.248686
C	-4.305457	1.607524	-0.801393
H	-4.592341	2.652063	-0.928443
C	-5.177535	0.601638	-1.195524
H	-6.143761	0.853580	-1.629179
C	-4.809662	-0.728752	-1.036290
H	-5.493385	-1.517125	-1.351362
N	-1.387815	-0.353546	0.392631
H	-2.528210	3.696486	-1.576967

1a-MesAlH2.log

SCF (wB97x) = -1006.60804023
E(SCF)+ZPE(0 K)= -1006.126309
H(298 K)= -1006.096414
G(298 K)= -1006.185227
Lowest Frequency = 15.8994cm^{-1}

N	1.421984	0.497184	0.163916
C	1.257695	1.611641	0.878165
Al	-0.000029	-0.564356	-0.629308
H	0.000030	-2.015214	-0.024845
H	-0.000088	-0.410290	-2.194890
N	-1.421996	0.497164	0.163998
C	0.000012	2.150549	1.188362
H	0.000025	3.068983	1.767416
C	-1.257682	1.611627	0.878244
C	2.472539	2.326290	1.396465
H	3.154054	2.593830	0.579506
H	3.049839	1.680732	2.070467
H	2.201006	3.236264	1.937303
C	-2.472500	2.326287	1.396593
H	-3.153871	2.594136	0.579614

H	-2.200916	3.236097	1.937684
H	-3.049977	1.680641	2.070352
C	2.742344	-0.016103	-0.048368
C	3.453778	0.354208	-1.200041
C	4.716364	-0.204353	-1.407043
H	5.276397	0.082376	-2.298984
C	5.277713	-1.115727	-0.512380
C	4.542886	-1.461794	0.623581
H	4.964911	-2.170871	1.338249
C	3.277790	-0.930981	0.873992
C	-2.742357	-0.016111	-0.048310
C	-3.453798	0.354289	-1.199947
C	-4.716376	-0.204277	-1.406994
H	-5.276413	0.082506	-2.298915
C	-5.277714	-1.115732	-0.512410
C	-4.542857	-1.461927	0.623495
H	-4.964844	-2.171102	1.338088
C	-3.277767	-0.931115	0.873947
C	6.620909	-1.727909	-0.773891
H	7.198685	-1.852062	0.149629
H	6.526410	-2.725655	-1.223695
H	7.216346	-1.120169	-1.464282
C	-6.620961	-1.727808	-0.773914
H	-6.526568	-2.725753	-1.223292
H	-7.198938	-1.851458	0.149551
H	-7.216176	-1.120219	-1.464633
C	-2.500592	-1.314160	2.096273
H	-2.287830	-0.448030	2.738756
H	-3.044999	-2.048966	2.698554
H	-1.525507	-1.744416	1.830450
C	-2.870181	1.334471	-2.171225
H	-2.684221	2.312923	-1.706456
H	-1.900995	0.991033	-2.556087
H	-3.539241	1.493421	-3.023372
C	2.870157	1.334331	-2.171375
H	1.901024	0.990818	-2.556301
H	2.684091	2.312772	-1.706627
H	3.539262	1.493317	-3.023480
C	2.500657	-1.313871	2.096393
H	1.525556	-1.744144	1.830664
H	3.045081	-2.048608	2.698743
H	2.287940	-0.447656	2.738778

7a.log

SCF (wB97x) = -4362.33907720
E(SCF)+ZPE(0 K)= -4360.410462
H(298 K)= -4360.307941
G(298 K)= -4360.544167
Lowest Frequency = 20.4042cm^{-1}

Pd	1.439740	-0.181195	0.203767
Al	-0.372577	-0.984191	1.720056
H	1.388908	-1.145591	1.765567
P	3.778829	-0.146707	-0.082960
N	-0.676149	-0.295301	3.532551
C	-1.417075	-0.915804	4.450540
C	-1.909818	-2.221789	4.287763
H	-2.557663	-2.589727	5.077819

C	-1.579974	-3.143115	3.282477
N	-0.846723	-2.826735	2.213642
C	-1.740349	-0.207807	5.736164
H	-0.836037	-0.019398	6.328372
H	-2.434742	-0.790862	6.347980
H	-2.185614	0.776195	5.537702
C	-2.083213	-4.550986	3.439088
H	-2.593520	-4.882917	2.525448
H	-2.778008	-4.632098	4.279877
H	-1.263133	-5.260597	3.604247
C	0.030767	0.896128	3.901824
C	1.237156	0.762609	4.612158
C	1.934655	1.916004	4.975358
H	2.868391	1.809311	5.532196
C	1.486676	3.190946	4.629360
C	0.300968	3.288287	3.900846
H	-0.074719	4.273648	3.616289
C	-0.440628	2.166388	3.527921
C	1.776709	-0.595323	4.939482
H	1.093422	-1.189539	5.563345
H	2.737164	-0.526586	5.462782
H	1.927439	-1.171584	4.014670
C	2.253178	4.422690	5.008050
H	1.619690	5.151509	5.529683
H	2.658469	4.934658	4.123534
H	3.099677	4.188653	5.663791
C	-1.713620	2.331664	2.763984
H	-1.713608	1.723939	1.841554
H	-1.865436	3.378227	2.472701
H	-2.590364	2.014490	3.350661
C	-0.284908	-3.877811	1.420999
C	-0.809397	-4.175301	0.151967
C	-0.185573	-5.158221	-0.616592
H	-0.597988	-5.384283	-1.602389
C	0.928857	-5.862293	-0.159317
C	1.409826	-5.564876	1.115734
H	2.269488	-6.116558	1.500571
C	0.835421	-4.570988	1.912331
C	-2.021398	-3.468807	-0.358008
H	-2.885775	-3.613061	0.308462
H	-2.295995	-3.823165	-1.358994
H	-1.857561	-2.378374	-0.415678
C	1.591378	-6.895177	-1.020630
H	0.873743	-7.632202	-1.402853
H	2.369938	-7.439529	-0.474211
H	2.069118	-6.438968	-1.899446
C	1.421748	-4.215636	3.245072
H	1.817613	-3.188674	3.223569
H	2.239940	-4.891865	3.516489
H	0.686974	-4.236651	4.060996
C	4.595037	-1.726518	0.494436
H	5.654738	-1.667935	0.191771
C	4.577114	-1.960102	2.005619
H	3.529410	-2.023988	2.345169
H	5.026208	-1.117098	2.543767
C	5.338232	-3.235282	2.359193
H	6.401351	-3.096854	2.099048
H	5.307236	-3.402730	3.445434
C	4.787970	-4.449224	1.624153

H	5.385815	-5.343618	1.849349
H	3.773982	-4.648200	1.996144
C	4.707005	-4.218490	0.120558
H	4.220272	-5.074647	-0.369456
H	5.725195	-4.156355	-0.300109
C	3.954355	-2.932554	-0.201113
H	3.908368	-2.783141	-1.288961
H	2.904316	-3.010922	0.135498
C	4.522473	0.028298	-1.822758
H	3.715184	-0.361275	-2.467080
C	5.790155	-0.771364	-2.157629
H	6.624023	-0.450499	-1.513783
H	5.644927	-1.839618	-1.954697
C	6.201090	-0.591340	-3.616475
H	7.106874	-1.177038	-3.824858
H	5.409091	-1.002299	-4.266428
C	6.409053	0.876856	-3.957010
H	6.705203	0.995402	-5.008075
H	7.239236	1.277621	-3.351934
C	5.136127	1.657221	-3.665804
H	4.334717	1.280990	-4.318417
H	5.257934	2.723191	-3.908581
C	4.705715	1.500470	-2.210833
H	3.770719	2.053376	-2.022792
H	5.478359	1.960090	-1.573059
C	4.662817	1.188635	0.882211
H	4.461363	2.094347	0.285438
C	6.185386	1.041025	0.980472
H	6.435866	0.142885	1.567885
H	6.623295	0.883878	-0.014402
C	6.820444	2.263687	1.635785
H	7.907909	2.129738	1.718214
H	6.664505	3.139247	0.983208
C	6.204318	2.542893	2.999990
H	6.653198	3.438965	3.449829
H	6.436964	1.705557	3.678951
C	4.691552	2.693807	2.894268
H	4.460425	3.581801	2.280759
H	4.252861	2.879870	3.883258
C	4.046549	1.465214	2.260687
H	4.187953	0.604285	2.930579
H	2.957337	1.587641	2.173660
Pd	-1.439663	0.181190	-0.203748
Al	0.372687	0.984223	-1.720020
H	-1.388830	1.145573	-1.765546
P	-3.778748	0.146573	0.082978
N	0.676245	0.295510	-3.532581
C	1.417220	0.916080	-4.450492
C	1.909969	2.222046	-4.287603
H	2.557823	2.590045	-5.077623
C	1.580078	3.143301	-3.282263
N	0.846836	2.826812	-2.213460
C	1.740507	0.208186	-5.736174
H	0.836193	0.019726	-6.328360
H	2.434836	0.791329	-6.347978
H	2.185868	-0.775788	-5.537775
C	2.083241	4.551206	-3.438792
H	2.593539	4.883116	-2.525138
H	2.778027	4.632405	-4.279581

H	1.263118	5.260778	-3.603900
C	-0.030699	-0.895854	-3.901995
C	-1.236994	-0.762216	-4.612466
C	-1.934539	-1.915548	-4.975786
H	-2.868213	-1.808768	-5.532713
C	-1.486683	-3.190536	-4.629800
C	-0.301073	-3.287992	-3.901138
H	0.074492	-4.273392	-3.616551
C	0.440556	-2.166160	-3.528081
C	-1.776359	0.595773	-4.939868
H	-1.092974	1.189845	-5.563761
H	-2.736813	0.527137	-5.463187
H	-1.927021	1.172119	-4.015097
C	-2.253202	-4.422208	-5.008688
H	-1.619815	-5.150793	-5.530774
H	-2.658187	-4.934522	-4.124234
H	-3.099910	-4.188001	-5.664098
C	1.713415	-2.331529	-2.763938
H	1.713286	-1.723847	-1.841482
H	1.865147	-3.378109	-2.472673
H	2.590269	-2.014383	-3.350463
C	0.284887	3.877797	-1.420785
C	0.809387	4.175418	-0.151787
C	0.185404	5.158234	0.616779
H	0.597848	5.384442	1.602527
C	-0.929266	5.861978	0.159598
C	-1.410222	5.564470	-1.115440
H	-2.270037	6.115948	-1.500232
C	-0.835585	4.570762	-1.912090
C	2.021555	3.469187	0.358161
H	2.885860	3.613511	-0.308390
H	2.296161	3.823703	1.359088
H	1.857887	2.378736	0.415970
C	-1.592135	6.894515	1.021054
H	-0.874379	7.630387	1.405242
H	-2.369357	7.440221	0.474078
H	-2.071693	6.437764	1.898607
C	-1.421791	4.215379	-3.244880
H	-1.817948	3.188529	-3.223323
H	-2.239754	4.891790	-3.516528
H	-0.686866	4.236085	-4.060669
C	-4.595174	1.726193	-0.494613
H	-5.654840	1.667548	-0.191837
C	-4.577432	1.959433	-2.005845
H	-3.529769	2.023364	-2.345507
H	-5.026469	1.116238	-2.543749
C	-5.338759	3.234426	-2.359631
H	-6.401838	3.095906	-2.099371
H	-5.307870	3.401638	-3.445911
C	-4.788628	4.448615	-1.624894
H	-5.386651	5.342856	-1.850221
H	-3.774717	4.647694	-1.997030
C	-4.707445	4.218224	-0.121259
H	-4.220775	5.074566	0.368495
H	-5.725576	4.156040	0.299546
C	-3.954584	2.932460	0.200613
H	-3.908452	2.783287	1.288488
H	-2.904591	3.010883	-0.136134
C	-4.522359	-0.028193	1.822835

H	-3.715073	0.361557	2.467057
C	-5.790047	0.771505	2.157578
H	-6.623944	0.450442	1.513869
H	-5.644876	1.839715	1.954359
C	-6.200880	0.591838	3.616495
H	-7.106664	1.177567	3.824790
H	-5.408846	1.002974	4.266293
C	-6.408785	-0.876280	3.957401
H	-6.704872	-0.994573	5.008511
H	-7.238990	-1.277219	3.352470
C	-5.135851	-1.656680	3.666315
H	-4.334419	-1.280258	4.318791
H	-5.257616	-2.722593	3.909369
C	-4.705513	-1.500281	2.211281
H	-3.770503	-2.053196	2.023339
H	-5.478168	-1.960098	1.573668
C	-4.662640	-1.188906	-0.882087
H	-4.461061	-2.094584	-0.285306
C	-6.185220	-1.041409	-0.980319
H	-6.435763	-0.143319	-1.567786
H	-6.623132	-0.884219	0.014544
C	-6.820209	-2.264153	-1.635547
H	-7.907689	-2.130300	-1.717941
H	-6.664172	-3.139668	-0.982932
C	-6.204119	-2.543371	-2.999765
H	-6.652928	-3.439514	-3.449534
H	-6.436884	-1.706094	-3.678758
C	-4.691334	-2.694118	-2.894114
H	-4.460069	-3.582064	-2.280587
H	-4.252680	-2.880169	-3.883123
C	-4.046430	-1.465440	-2.260603
H	-4.187989	-0.604529	-2.930491
H	-2.957197	-1.587734	-2.173646

4-PdAlMesH2P2.log

SCF (wB97x) = -3228.81664856
E(SCF)+ZPE(0 K)= -3227.359978
H(298 K)= -3227.286621
G(298 K)= -3227.468551
Lowest Frequency = 15.1774cm^{-1}

Al	-2.193010	-0.095232	-0.069085
H	-2.940799	0.915833	0.887078
P	0.974683	1.671788	1.406167
N	-3.162581	0.082031	-1.769275
C	-3.593067	-0.966270	-2.463086
C	-3.614699	-2.274698	-1.945078
H	-3.957330	-3.048247	-2.626617
C	-3.409911	-2.677662	-0.613171
N	-2.956524	-1.863372	0.338185
C	-4.124951	-0.757630	-3.852816
H	-5.010883	-0.109548	-3.836889
H	-4.399596	-1.705408	-4.323891
H	-3.389481	-0.248899	-4.489096
C	-3.776941	-4.090508	-0.255118
H	-2.947550	-4.606051	0.244941
H	-4.064239	-4.665377	-1.139777
H	-4.615535	-4.107429	0.453384

C	-3.276260	1.401593	-2.301575
C	-4.405217	2.168891	-1.969527
C	-4.480330	3.484252	-2.429773
H	-5.357518	4.081610	-2.173378
C	-3.465091	4.055147	-3.198750
C	-2.356445	3.267336	-3.512268
H	-1.551083	3.694754	-4.113344
C	-2.235563	1.947375	-3.073854
C	-5.488264	1.576163	-1.122234
H	-5.897436	0.652742	-1.555210
H	-6.315663	2.279811	-0.980888
H	-5.095755	1.300606	-0.133444
C	-3.540071	5.485979	-3.639086
H	-3.093842	5.632296	-4.629833
H	-2.997054	6.145648	-2.946955
H	-4.574347	5.846157	-3.678859
C	-1.010891	1.146268	-3.396985
H	-0.358410	1.032598	-2.512186
H	-0.422222	1.622808	-4.189807
H	-1.254194	0.122112	-3.714230
C	-2.936449	-2.298204	1.698539
C	-1.804337	-2.943671	2.227273
C	-1.816958	-3.313072	3.573196
H	-0.933790	-3.804056	3.988500
C	-2.907405	-3.052594	4.404776
C	-4.006900	-2.391120	3.856302
H	-4.862804	-2.157525	4.492417
C	-4.042368	-2.006297	2.514919
C	-0.606481	-3.223015	1.373074
H	-0.839205	-3.894533	0.533347
H	0.195005	-3.687898	1.960451
H	-0.206095	-2.298631	0.920687
C	-2.882396	-3.452953	5.849211
H	-3.201149	-4.495019	5.989655
H	-3.550122	-2.828115	6.453505
H	-1.873226	-3.368989	6.271550
C	-5.219846	-1.277098	1.945084
H	-4.921650	-0.282108	1.586113
H	-6.011602	-1.151569	2.691394
H	-5.649587	-1.796617	1.077198
C	0.380020	1.352630	3.144399
H	0.854117	2.104294	3.798646
C	-1.130307	1.459248	3.354149
H	-1.639425	0.702541	2.737688
H	-1.514397	2.430248	3.014668
C	-1.479196	1.243842	4.823603
H	-1.022763	2.047365	5.426510
H	-2.565541	1.326596	4.967061
C	-0.988318	-0.111926	5.313590
H	-1.209304	-0.243462	6.382560
H	-1.546430	-0.895326	4.781009
C	0.500128	-0.306670	5.048175
H	0.807755	-1.324517	5.330860
H	1.083815	0.381088	5.683422
C	0.842772	-0.042848	3.585583
H	1.921927	-0.180380	3.413436
H	0.337037	-0.785603	2.940455
C	2.830288	1.942208	1.732096
H	3.248346	0.934954	1.548340

C	3.287303	2.362102	3.136092
H	2.875716	3.351842	3.389413
H	2.906546	1.670561	3.897673
C	4.810258	2.416877	3.232795
H	5.110520	2.720762	4.244898
H	5.213462	1.401422	3.082352
C	5.408595	3.348918	2.188925
H	6.504218	3.364256	2.263411
H	5.069195	4.379442	2.384321
C	4.970687	2.930658	0.792180
H	5.387964	1.935017	0.571672
H	5.379457	3.612564	0.032856
C	3.449465	2.877149	0.686981
H	3.138651	2.572128	-0.326693
H	3.059209	3.897580	0.835576
C	0.375920	3.388115	0.962055
H	1.075984	3.699679	0.167616
C	0.457199	4.432163	2.079505
H	-0.193594	4.131685	2.916582
H	1.476332	4.477549	2.488069
C	0.035216	5.814157	1.587375
H	0.080365	6.539066	2.411858
H	0.759068	6.160411	0.830476
C	-1.356365	5.782836	0.968650
H	-1.641090	6.781811	0.610680
H	-2.090442	5.510804	1.745075
C	-1.424244	4.764528	-0.162622
H	-0.754977	5.085734	-0.979663
H	-2.434204	4.721578	-0.592863
C	-1.011166	3.375189	0.308853
H	-1.764558	2.987567	1.010086
H	-1.008261	2.668157	-0.535626
Pd	0.385810	0.036774	-0.134469
H	-0.817743	-0.847614	-0.931800
P	2.010042	-1.308078	-1.381525
C	3.516631	-0.593028	-2.212634
H	3.973117	-1.358511	-2.863765
C	4.579741	-0.127348	-1.218816
H	4.105824	0.555946	-0.495360
H	4.960651	-0.975054	-0.633496
C	5.741338	0.573726	-1.916917
H	6.266181	-0.153630	-2.558125
H	6.477305	0.915362	-1.175588
C	5.262158	1.738974	-2.772152
H	6.108619	2.220311	-3.280246
H	4.814840	2.505581	-2.117440
C	4.217590	1.276815	-3.780018
H	3.852015	2.123902	-4.376181
H	4.682710	0.574636	-4.491872
C	3.054178	0.587176	-3.078390
H	2.301207	0.260229	-3.811439
H	2.535959	1.309700	-2.421388
C	1.136849	-2.117643	-2.842930
H	0.494485	-1.294403	-3.203458
C	1.962130	-2.600583	-4.041086
H	2.607045	-3.442250	-3.740914
H	2.635220	-1.814444	-4.407302
C	1.047842	-3.048238	-5.180224
H	1.647995	-3.391115	-6.034197

H	0.474809	-2.174889	-5.535434
C	0.076367	-4.133439	-4.733803
H	-0.589175	-4.418220	-5.560023
H	0.645399	-5.039828	-4.468649
C	-0.727548	-3.674946	-3.524486
H	-1.374706	-2.827872	-3.808302
H	-1.403791	-4.469622	-3.176541
C	0.187519	-3.231466	-2.388804
H	-0.411893	-2.890246	-1.532133
H	0.779403	-4.098913	-2.047142
C	2.616597	-2.737908	-0.347556
H	1.714843	-3.367079	-0.240689
C	3.718711	-3.613348	-0.947491
H	4.616563	-3.004100	-1.140217
H	3.402203	-4.004116	-1.924691
C	4.087451	-4.766826	-0.017663
H	4.902118	-5.360311	-0.454619
H	3.222959	-5.445648	0.071057
C	4.469021	-4.265881	1.370215
H	4.725931	-5.108450	2.026108
H	5.376552	-3.643987	1.294293
C	3.342048	-3.436386	1.974558
H	2.458993	-4.082619	2.113552
H	3.616456	-3.070390	2.974062
C	2.968714	-2.267374	1.071024
H	3.809508	-1.555338	1.038023
H	2.117065	-1.703190	1.481827

4-monophosphine-PdAlMesH2P.log

SCF (wB97x) = -2181.71931219
E(SCF)+ZPE(0 K)= -2180.750032
H(298 K)= -2180.697033
G(298 K)= -2180.839270
Lowest Frequency = 7.4533cm^{-1}

Al	-1.918946	0.607579	0.061504
H	-1.770903	0.190341	-1.446404
N	-3.836820	0.674150	0.434637
C	-4.507186	1.736011	0.879222
C	-3.909943	2.988051	1.094657
H	-4.559607	3.772034	1.471693
C	-2.593089	3.363786	0.796287
N	-1.668292	2.514662	0.338857
C	-5.982418	1.613987	1.134093
H	-6.520006	1.373000	0.208044
H	-6.395220	2.540455	1.541266
H	-6.201259	0.795194	1.830237
C	-2.226699	4.809603	0.974041
H	-1.364414	4.926067	1.641814
H	-3.062918	5.385209	1.378872
H	-1.928425	5.256461	0.016954
C	-4.556176	-0.526714	0.126924
C	-5.059956	-0.698764	-1.173611
C	-5.713834	-1.892021	-1.481454
H	-6.108538	-2.028872	-2.489996
C	-5.876027	-2.909105	-0.539520
C	-5.375329	-2.700229	0.745994
H	-5.504740	-3.476921	1.502071

C	-4.712041	-1.524658	1.102362
C	-4.905639	0.386322	-2.195302
H	-5.387216	1.322758	-1.879333
H	-5.345580	0.092937	-3.154344
H	-3.847049	0.627000	-2.362156
C	-6.551078	-4.196980	-0.904281
H	-7.032233	-4.663564	-0.037079
H	-5.833321	-4.927625	-1.301718
H	-7.315585	-4.051279	-1.676170
C	-4.197159	-1.325043	2.495231
H	-3.104660	-1.221464	2.511416
H	-4.467232	-2.168565	3.139405
H	-4.595449	-0.410526	2.955767
C	-0.372667	3.020946	-0.007114
C	0.639806	3.078126	0.964935
C	1.907342	3.510972	0.573349
H	2.698701	3.555510	1.324446
C	2.192721	3.879150	-0.742545
C	1.154120	3.844750	-1.674038
H	1.349859	4.144460	-2.705426
C	-0.128701	3.413984	-1.333004
C	0.368770	2.667954	2.378904
H	-0.522784	3.155337	2.795523
H	1.220411	2.900565	3.028912
H	0.184542	1.580625	2.434065
C	3.583531	4.262070	-1.150001
H	4.131779	4.744524	-0.332321
H	3.585952	4.943847	-2.008318
H	4.169193	3.378149	-1.445699
C	-1.217625	3.341415	-2.359976
H	-1.531116	2.301260	-2.529346
H	-0.885833	3.754750	-3.318703
H	-2.120565	3.885482	-2.050150
Pd	0.145035	-0.606040	0.983635
H	-1.471445	-0.244809	1.476974
P	2.236276	-1.117789	0.183229
C	2.424129	-2.937605	-0.158500
H	3.381415	-3.113695	-0.678534
C	1.278818	-3.405913	-1.065044
H	0.325439	-3.165102	-0.563713
H	1.269316	-2.835117	-2.005024
C	1.357869	-4.900626	-1.350199
H	2.278894	-5.116729	-1.917318
H	0.520963	-5.206921	-1.991982
C	1.363457	-5.703384	-0.055298
H	1.445655	-6.778472	-0.264714
H	0.400731	-5.559023	0.460652
C	2.494087	-5.250604	0.860144
H	2.474638	-5.809167	1.805767
H	3.460141	-5.485192	0.383147
C	2.430438	-3.751666	1.138685
H	3.277397	-3.459224	1.775700
H	1.515079	-3.515264	1.709281
C	3.641059	-0.745879	1.348223
H	3.411002	-1.356453	2.238543
C	5.048419	-1.107309	0.872719
H	5.293611	-0.500170	-0.014845
H	5.099263	-2.159714	0.555136
C	6.082582	-0.827624	1.960535

H	7.089237	-1.085833	1.604733
H	5.881462	-1.483171	2.823683
C	6.030520	0.628356	2.410249
H	6.762749	0.811533	3.207992
H	6.324152	1.274400	1.566469
C	4.628192	1.013435	2.868013
H	4.373712	0.444860	3.777100
H	4.591055	2.075804	3.149370
C	3.591644	0.717951	1.790289
H	2.576481	0.964102	2.135184
H	3.782028	1.368042	0.919024
C	2.767622	-0.282974	-1.410469
H	3.353602	0.591947	-1.070354
C	3.667008	-1.110652	-2.336545
H	3.114983	-1.998946	-2.683114
H	4.547158	-1.489841	-1.800596
C	4.097154	-0.300924	-3.556203
H	4.744377	-0.908824	-4.203152
H	4.707286	0.556463	-3.224310
C	2.888073	0.212490	-4.328035
H	3.207043	0.800822	-5.199122
H	2.322492	-0.646975	-4.723715
C	1.986081	1.038437	-3.420191
H	2.532541	1.941416	-3.101165
H	1.099480	1.394608	-3.964181
C	1.559453	0.259741	-2.181649
H	0.903944	-0.578314	-2.472975
H	0.950670	0.887078	-1.514200

7e-PhAl2Pd2P2H2.log

SCF (wB97x) = -3890.58255242
E(SCF)+ZPE(0 K)= -3888.991302
H(298 K)= -3888.906828
G(298 K)= -3889.115992
Lowest Frequency = 5.8811cm^{-1}

Pd	-0.451072	0.831514	1.070185
Al	1.458063	1.340325	-0.483071
H	0.785234	2.193764	0.894417
P	-1.646241	1.593549	2.908208
N	3.370504	1.611848	-0.130481
C	4.178903	2.471604	-0.754771
C	3.747262	3.324802	-1.778433
H	4.516858	3.928007	-2.251214
C	2.454284	3.451946	-2.310201
N	1.417499	2.739276	-1.872207
C	5.641925	2.500773	-0.410375
H	5.816004	2.869003	0.607121
H	6.195521	3.139413	-1.103900
H	6.067685	1.489575	-0.446236
C	2.287257	4.399279	-3.467607
H	1.666023	3.949938	-4.252521
H	3.260114	4.661986	-3.893570
H	1.790011	5.330483	-3.174017
C	3.896755	0.811070	0.921062
C	4.174773	1.371634	2.171026
C	4.708961	0.585319	3.188235
H	4.931566	1.034407	4.155764

C	4.958582	-0.767779	2.967110
H	5.379836	-1.383060	3.761111
C	4.641938	-1.337590	1.735160
H	4.801581	-2.402072	1.568294
C	4.103550	-0.556426	0.716978
C	0.115011	2.989153	-2.384421
C	-0.576631	1.957796	-3.029844
C	-1.863661	2.171840	-3.509788
H	-2.393026	1.356057	-4.001450
C	-2.478397	3.411780	-3.343774
H	-3.486804	3.577485	-3.720946
C	-1.803986	4.432013	-2.676674
H	-2.283637	5.398689	-2.528141
C	-0.517718	4.220512	-2.187255
C	-2.979511	2.801337	2.413612
H	-3.812297	2.146676	2.101470
C	-2.573548	3.586596	1.157794
H	-1.691411	4.211746	1.367885
H	-2.251466	2.883825	0.371548
C	-3.718151	4.467064	0.671054
H	-3.407891	5.031369	-0.219216
H	-4.557020	3.823561	0.353122
C	-4.208155	5.403976	1.768718
H	-3.392824	6.091721	2.048371
H	-5.032604	6.032606	1.405673
C	-4.641671	4.614714	2.997817
H	-5.506871	3.984459	2.732223
H	-4.984658	5.289471	3.794169
C	-3.517243	3.720371	3.513558
H	-3.874728	3.124491	4.365235
H	-2.702297	4.350802	3.903743
C	-2.517581	0.385071	4.073183
H	-1.871013	-0.511555	4.026158
C	-3.881200	-0.036209	3.514761
H	-4.566184	0.828506	3.539598
H	-3.781862	-0.330990	2.457742
C	-4.505660	-1.163157	4.331172
H	-5.480323	-1.441975	3.904662
H	-3.870971	-2.059009	4.252307
C	-4.638713	-0.771997	5.796172
H	-5.337512	0.075913	5.888130
H	-5.072203	-1.594947	6.380492
C	-3.283348	-0.368359	6.360248
H	-2.608916	-1.241108	6.335675
H	-3.370221	-0.075003	7.415376
C	-2.651910	0.763886	5.553626
H	-1.675391	1.014091	5.987273
H	-3.273226	1.668082	5.655287
C	-0.534053	2.503192	4.102026
H	-1.128559	2.780983	4.989481
C	0.582759	1.538230	4.527108
H	0.163198	0.602415	4.928729
H	1.146889	1.253508	3.619660
C	1.527497	2.169187	5.542056
H	0.974318	2.386461	6.471431
H	2.319738	1.456788	5.813897
C	2.126912	3.459297	4.997062
H	2.764324	3.215881	4.131592
H	2.780310	3.931210	5.743819

C	1.034916	4.424826	4.552034
H	1.478188	5.335029	4.125122
H	0.458938	4.748188	5.435446
C	0.087851	3.783136	3.540953
H	0.630799	3.536934	2.614086
H	-0.691969	4.504548	3.260246
Pd	0.451072	-0.831514	-1.070185
Al	-1.458063	-1.340325	0.483071
H	-0.785234	-2.193764	-0.894417
P	1.646241	-1.593549	-2.908208
N	-3.370504	-1.611848	0.130481
C	-4.178903	-2.471604	0.754771
C	-3.747262	-3.324802	1.778433
H	-4.516858	-3.928007	2.251214
C	-2.454284	-3.451946	2.310201
N	-1.417499	-2.739276	1.872207
C	-5.641925	-2.500773	0.410375
H	-5.816004	-2.869003	-0.607121
H	-6.195521	-3.139413	1.103900
H	-6.067685	-1.489575	0.446236
C	-2.287257	-4.399279	3.467607
H	-1.666023	-3.949938	4.252521
H	-3.260114	-4.661986	3.893570
H	-1.790011	-5.330483	3.174017
C	-3.896755	-0.811070	-0.921062
C	-4.174773	-1.371634	-2.171026
C	-4.708961	-0.585319	-3.188235
H	-4.931566	-1.034407	-4.155764
C	-4.958582	0.767779	-2.967110
H	-5.379836	1.383060	-3.761111
C	-4.641938	1.337590	-1.735160
H	-4.801581	2.402072	-1.568294
C	-4.103550	0.556426	-0.716978
C	-0.115011	-2.989153	2.384421
C	0.576631	-1.957796	3.029844
C	1.863661	-2.171840	3.509788
H	2.393026	-1.356057	4.001450
C	2.478397	-3.411780	3.343774
H	3.486804	-3.577485	3.720946
C	1.803986	-4.432013	2.676674
H	2.283637	-5.398689	2.528141
C	0.517718	-4.220512	2.187255
C	2.979511	-2.801337	-2.413612
H	3.812297	-2.146676	-2.101470
C	2.573548	-3.586596	-1.157794
H	1.691411	-4.211746	-1.367885
H	2.251466	-2.883825	-0.371548
C	3.718151	-4.467064	-0.671054
H	3.407891	-5.031369	0.219216
H	4.557020	-3.823561	-0.353122
C	4.208155	-5.403976	-1.768718
H	3.392824	-6.091721	-2.048371
H	5.032604	-6.032606	-1.405673
C	4.641671	-4.614714	-2.997817
H	5.506871	-3.984459	-2.732223
H	4.984658	-5.289471	-3.794169
C	3.517243	-3.720371	-3.513558
H	3.874728	-3.124491	-4.365235
H	2.702297	-4.350802	-3.903743

C	2.517581	-0.385071	-4.073183
H	1.871013	0.511555	-4.026158
C	3.881200	0.036209	-3.514761
H	4.566184	-0.828506	-3.539598
H	3.781862	0.330990	-2.457742
C	4.505660	1.163157	-4.331172
H	5.480323	1.441975	-3.904662
H	3.870971	2.059009	-4.252307
C	4.638713	0.771997	-5.796172
H	5.337512	-0.075913	-5.888130
H	5.072203	1.594947	-6.380492
C	3.283348	0.368359	-6.360248
H	2.608916	1.241108	-6.335675
H	3.370221	0.075003	-7.415376
C	2.651910	-0.763886	-5.553626
H	1.675391	-1.014091	-5.987273
H	3.273226	-1.668082	-5.655287
C	0.534053	-2.503192	-4.102026
H	1.128559	-2.780983	-4.989481
C	-0.582759	-1.538230	-4.527108
H	-0.163198	-0.602415	-4.928729
H	-1.146889	-1.253508	-3.619660
C	-1.527497	-2.169187	-5.542056
H	-0.974318	-2.386461	-6.471431
H	-2.319738	-1.456788	-5.813897
C	-2.126912	-3.459297	-4.997062
H	-2.764324	-3.215881	-4.131592
H	-2.780310	-3.931210	-5.743819
C	-1.034916	-4.424826	-4.552034
H	-1.478188	-5.335029	-4.125122
H	-0.458938	-4.748188	-5.435446
C	-0.087851	-3.783136	-3.540953
H	-0.630799	-3.536934	-2.614086
H	0.691969	-4.504548	-3.260246
H	3.972314	2.431635	2.326040
H	3.835993	-0.988264	-0.249229
H	-0.001962	-5.002199	1.633598
H	0.099593	-0.981098	3.124021
H	-3.835993	0.988264	0.249229
H	-3.972314	-2.431635	-2.326040
H	0.001962	5.002199	-1.633598
H	-0.099593	0.981098	-3.124021

1e-PhAlH2.log

SCF (wB97x) = -770.734658356
E(SCF)+ZPE(0 K)= -770.420016
H(298 K)= -770.400058
G(298 K)= -770.469342
Lowest Frequency = 16.1265cm^{-1}

N	1.433807	0.324259	-0.163658
C	1.263184	1.644348	-0.267141
Al	0.000016	-1.003071	-0.222655
H	0.000229	-1.850412	1.096244
H	-0.000227	-1.737904	-1.614248
N	-1.433713	0.324236	-0.163393
C	0.000003	2.248480	-0.358414
H	-0.000015	3.327119	-0.484188

C	-1.263144	1.644322	-0.266891
C	2.458159	2.554932	-0.316840
H	3.262740	2.125516	-0.923789
H	2.878210	2.726823	0.681117
H	2.183052	3.529116	-0.729732
C	-2.458218	2.554776	-0.316314
H	-3.262313	2.125855	-0.924276
H	-2.183085	3.529483	-0.727936
H	-2.878996	2.725370	0.681556
C	2.731784	-0.230159	0.015438
C	3.225300	-1.126026	-0.939450
C	4.465073	-1.731907	-0.754807
H	4.841677	-2.422199	-1.507495
C	5.219906	-1.457598	0.382463
C	4.723326	-0.578123	1.342820
H	5.298978	-0.371454	2.243478
C	3.484680	0.028252	1.166426
C	-2.731743	-0.230154	0.015617
C	-3.225855	-1.124870	-0.940027
C	-4.465696	-1.730657	-0.755501
H	-4.842786	-2.420065	-1.508755
C	-5.219972	-1.457383	0.382388
C	-4.722783	-0.579055	1.343480
H	-5.298011	-0.373214	2.244598
C	-3.484067	0.027217	1.167202
H	6.187542	-1.934211	0.525754
H	-6.187659	-1.933923	0.525581
H	-3.076634	0.688907	1.930246
H	-2.630193	-1.330800	-1.828478
H	2.629255	-1.332737	-1.827466
H	3.077745	0.690818	1.928970

3-RuN2PH4AlDipp.log

SCF (wB97x) = -2495.28681350
E(SCF)+ZPE(0 K)= -2494.118800
H(298 K)= -2494.057369
G(298 K)= -2494.211162
Lowest Frequency = 23.2343cm^{-1}

Ru	0.381094	-0.763913	-0.661656
Al	-1.609497	0.198959	0.149158
P	2.606994	-0.543236	-0.083176
H	0.251531	0.906257	-0.717773
H	-1.230473	-0.681411	-1.353967
H	-0.390026	-0.539517	1.090180
H	0.915172	-0.692488	-2.186679
N	-2.200109	1.986605	0.433782
C	-3.423572	2.323740	0.885234
C	-4.426046	1.394454	1.178875
H	-5.354002	1.798420	1.571584
C	-4.401477	0.012817	0.935812
N	-3.325416	-0.601771	0.447017
C	-3.760906	3.774242	1.076043
H	-3.186498	4.215486	1.900315
H	-4.823932	3.902268	1.294423
H	-3.508366	4.359182	0.183401
C	-5.636919	-0.781598	1.233202
H	-5.924809	-1.413143	0.385250

H	-6.476709	-0.134851	1.499559
H	-5.449700	-1.466652	2.071108
C	-1.277366	3.051809	0.138646
C	-0.618026	3.715876	1.189725
C	0.245929	4.764346	0.864354
H	0.770291	5.291081	1.661247
C	0.459503	5.133482	-0.456837
H	1.138390	5.952141	-0.692540
C	-0.165319	4.433116	-1.482226
H	0.036658	4.706664	-2.516402
C	-1.035138	3.375056	-1.211079
C	-0.757407	3.264894	2.631096
H	-1.690149	2.692136	2.728127
C	0.389514	2.315179	2.981334
H	0.434456	1.470957	2.279446
H	0.277489	1.912296	3.996715
H	1.354420	2.839027	2.928838
C	-0.826429	4.421965	3.623008
H	0.122501	4.969898	3.678193
H	-1.038642	4.049122	4.631913
H	-1.608011	5.146064	3.359199
C	-1.696983	2.601390	-2.335958
H	-1.791143	1.554255	-2.008408
C	-3.109363	3.112635	-2.621866
H	-3.770959	2.999398	-1.752814
H	-3.561820	2.555689	-3.452890
H	-3.096764	4.175223	-2.900270
C	-0.854408	2.560329	-3.604349
H	-0.801990	3.538322	-4.101044
H	-1.288728	1.855423	-4.322661
H	0.167404	2.225146	-3.387634
C	-3.432920	-1.956943	-0.025867
C	-3.677738	-2.147120	-1.402562
C	-3.739914	-3.456763	-1.880303
H	-3.936051	-3.628896	-2.938040
C	-3.542408	-4.542055	-1.034248
H	-3.582783	-5.555481	-1.429218
C	-3.276718	-4.329691	0.310976
H	-3.098969	-5.181980	0.966925
C	-3.216228	-3.038674	0.843319
C	-3.895056	-0.979892	-2.350025
H	-3.519066	-0.068361	-1.859996
C	-5.383702	-0.756956	-2.620464
H	-5.836088	-1.644027	-3.083761
H	-5.534036	0.089504	-3.302705
H	-5.941253	-0.543333	-1.699787
C	-3.120729	-1.131590	-3.655883
H	-2.050355	-1.280359	-3.468894
H	-3.238254	-0.230178	-4.271275
H	-3.484791	-1.976741	-4.253976
C	-2.889376	-2.851019	2.311051
H	-2.989432	-1.781644	2.549191
C	-1.442889	-3.254684	2.598939
H	-1.283065	-4.320341	2.386937
H	-1.191422	-3.079183	3.653095
H	-0.741999	-2.684213	1.977940
C	-3.848163	-3.626701	3.213617
H	-4.898856	-3.378913	3.015674
H	-3.643530	-3.415029	4.270208

H	-3.739219	-4.709692	3.072463
N	0.401776	-2.746312	-0.640335
N	0.369497	-3.870332	-0.584701
C	3.773741	-1.859915	-0.728762
H	3.621829	-2.690931	-0.014322
C	5.265964	-1.503839	-0.719959
H	5.449863	-0.718574	-1.472598
H	5.576746	-1.080131	0.243105
C	6.119499	-2.723298	-1.059499
H	7.184915	-2.454679	-1.048108
H	5.983423	-3.484134	-0.273241
C	5.729932	-3.316142	-2.407904
H	6.322321	-4.217021	-2.618157
H	5.976546	-2.591527	-3.201263
C	4.238370	-3.623869	-2.463258
H	4.003474	-4.430776	-1.749175
H	3.960287	-4.002489	-3.456067
C	3.398961	-2.399421	-2.115171
H	3.568583	-1.614103	-2.869398
H	2.331499	-2.630511	-2.168576
C	3.438913	1.053234	-0.543936
H	4.497998	0.951685	-0.248155
C	3.390616	1.298288	-2.054315
H	2.334497	1.370470	-2.359138
H	3.803629	0.438723	-2.600500
C	4.139180	2.571011	-2.433320
H	5.209168	2.448939	-2.193529
H	4.083266	2.734046	-3.518393
C	3.587080	3.775773	-1.682200
H	2.545557	3.945909	-2.000475
H	4.147233	4.685883	-1.938970
C	3.607453	3.539800	-0.176975
H	3.168886	4.396849	0.354247
H	4.653926	3.464999	0.164695
C	2.864231	2.261812	0.199237
H	1.794494	2.364185	-0.051911
H	2.912970	2.110453	1.287584
C	2.840788	-0.594680	1.769478
H	2.123650	0.180834	2.090108
C	4.195629	-0.231730	2.382352
H	4.920812	-1.035916	2.179862
H	4.610448	0.684285	1.937429
C	4.052433	-0.068775	3.894299
H	5.015703	0.208318	4.344215
H	3.362086	0.767995	4.096364
C	3.510280	-1.341305	4.536708
H	4.258191	-2.142846	4.421595
H	3.376136	-1.200089	5.617794
C	2.204253	-1.787352	3.886909
H	1.413035	-1.049516	4.105359
H	1.865894	-2.739534	4.318510
C	2.343769	-1.910842	2.373381
H	3.057527	-2.718158	2.136104
H	1.384151	-2.195623	1.921135

1c-xylAlH2.log

SCF (wB97x) = -927.986416364
E(SCF)+ZPE(0 K)= -927.560467

H(298 K)= -927.534018
G(298 K)= -927.615173
Lowest Frequency = 20.8261cm^{-1}

N	-1.422258	-0.202660	0.291899
C	-1.257590	-0.492544	1.583588
Al	0.000004	-0.034467	-1.023691
H	0.000003	1.411033	-1.640001
H	0.000006	-1.265055	-2.003514
N	1.422261	-0.202661	0.291902
C	-0.000001	-0.665850	2.180872
H	-0.000002	-0.913159	3.238023
C	1.257590	-0.492543	1.583591
C	-2.471287	-0.621028	2.458472
H	-3.168039	-1.370738	2.063798
H	-3.031678	0.321938	2.492251
H	-2.200420	-0.898511	3.480141
C	2.471287	-0.621044	2.458475
H	3.168013	-1.370784	2.063812
H	2.200415	-0.898500	3.480150
H	3.031708	0.321904	2.492233
C	-2.740762	0.040739	-0.211639
C	-3.474885	-1.020241	-0.767368
C	-4.735764	-0.742569	-1.300592
H	-5.315176	-1.557791	-1.733784
C	-5.250213	0.549124	-1.287098
C	-4.506375	1.587215	-0.736592
H	-4.904864	2.601641	-0.727938
C	-3.241742	1.353684	-0.192565
C	2.740764	0.040751	-0.211634
C	3.474908	-1.020234	-0.767325
C	4.735785	-0.742557	-1.300550
H	5.315214	-1.557782	-1.733711
C	5.250212	0.549147	-1.287095
C	4.506352	1.587243	-0.736629
H	4.904822	2.601677	-0.728012
C	3.241720	1.353707	-0.192603
C	2.429620	2.463906	0.401133
H	2.222334	2.299755	1.468111
H	2.944458	3.425729	0.307274
H	1.451445	2.549666	-0.091062
C	2.914890	-2.409877	-0.774118
H	2.735603	-2.786052	0.242902
H	1.946452	-2.452356	-1.289464
H	3.596632	-3.108350	-1.270430
C	-2.914842	-2.409875	-0.774206
H	-1.946401	-2.452317	-1.289548
H	-2.735555	-2.786084	0.242801
H	-3.596569	-3.108341	-1.270548
C	-2.429675	2.463872	0.401233
H	-1.451485	2.549660	-0.090927
H	-2.944523	3.425691	0.307388
H	-2.222424	2.299689	1.468213
H	-6.233036	0.748286	-1.710448
H	6.233033	0.748313	-1.710448

7c-xylPd2Al2H2P2.log

SCF (wB97x) = -4205.09131726

E(SCF)+ZPE(0 K)= -4203.274436
H(298 K)= -4203.178631
G(298 K)= -4203.401395
Lowest Frequency = 19.0717cm^{-1}

Pd	-0.486682	0.855641	1.078265
Al	1.429872	1.358777	-0.443244
H	0.745124	2.217421	0.936275
P	-1.686305	1.708138	2.920953
N	3.338455	1.625913	-0.048901
C	4.190251	2.210784	-0.895183
C	3.780418	2.909237	-2.039914
H	4.573907	3.294051	-2.673420
C	2.470712	3.268852	-2.400633
N	1.390365	2.807850	-1.771766
C	5.666854	2.135465	-0.622691
H	5.947257	2.711595	0.267789
H	6.244624	2.517193	-1.469253
H	5.971689	1.099796	-0.423830
C	2.304202	4.234321	-3.539965
H	1.612365	3.833884	-4.292934
H	3.261877	4.450531	-4.022110
H	1.869158	5.182530	-3.199727
C	3.817885	1.239304	1.243090
C	4.031944	2.242389	2.208039
C	4.488562	1.865398	3.473110
H	4.673685	2.638284	4.219500
C	4.697347	0.527347	3.791248
H	5.047898	0.250528	4.784378
C	4.466019	-0.450989	2.832249
H	4.638910	-1.501927	3.065886
C	4.032978	-0.115279	1.545957
C	3.737342	3.676839	1.889991
H	4.253046	4.036471	0.989518
H	4.016231	4.335332	2.720197
H	2.662541	3.809528	1.694218
C	3.824869	-1.169668	0.509589
H	2.778380	-1.189266	0.157686
H	4.070423	-2.165113	0.897916
H	4.439363	-0.985607	-0.385131
C	0.130676	3.451576	-1.999524
C	-0.827914	2.879892	-2.852869
C	-2.047578	3.543636	-3.020808
H	-2.790527	3.104488	-3.687071
C	-2.312157	4.742249	-2.370763
H	-3.266220	5.246885	-2.518283
C	-1.356053	5.289114	-1.521237
H	-1.562309	6.218910	-0.989160
C	-0.133522	4.651157	-1.310400
C	-0.555044	1.606400	-3.585258
H	-1.443555	1.264099	-4.128760
H	-0.249878	0.798464	-2.896140
H	0.261946	1.720530	-4.315346
C	0.866236	5.205546	-0.344089
H	1.069558	4.468802	0.446406
H	0.496683	6.123023	0.127902
H	1.835961	5.431454	-0.810669
C	-3.027237	2.938167	2.492949
H	-3.844584	2.291179	2.131655

C	-2.675800	3.848913	1.308449
H	-1.857572	4.534945	1.575210
H	-2.291707	3.252301	0.468672
C	-3.894421	4.667632	0.892723
H	-3.642007	5.317424	0.045016
H	-4.682538	3.983699	0.533771
C	-4.439194	5.489379	2.054747
H	-3.678388	6.225618	2.363643
H	-5.319502	6.067706	1.742751
C	-4.779627	4.596860	3.240607
H	-5.605630	3.922351	2.958773
H	-5.144333	5.194306	4.087574
C	-3.579860	3.755352	3.665881
H	-3.865656	3.095956	4.496355
H	-2.792159	4.417631	4.060453
C	-2.563179	0.525501	4.122167
H	-1.994721	-0.412079	3.992547
C	-3.996329	0.222663	3.668407
H	-4.610724	1.134851	3.743277
H	-3.995483	-0.071649	2.605976
C	-4.646647	-0.860710	4.522772
H	-5.670551	-1.053656	4.170333
H	-4.091021	-1.800956	4.391269
C	-4.632978	-0.489447	5.997916
H	-5.233393	0.421288	6.158858
H	-5.099578	-1.279800	6.601330
C	-3.202928	-0.236360	6.451393
H	-2.621435	-1.168518	6.343811
H	-3.167814	0.028628	7.516932
C	-2.541453	0.860121	5.621090
H	-1.514411	1.008192	5.976145
H	-3.066856	1.809926	5.806745
C	-0.575015	2.641082	4.098526
H	-1.181857	2.884895	4.987395
C	0.589935	1.743486	4.531467
H	0.228142	0.764489	4.876243
H	1.216368	1.535656	3.644670
C	1.424912	2.410917	5.617540
H	0.805422	2.543921	6.520942
H	2.261972	1.759249	5.906747
C	1.946323	3.762946	5.148901
H	2.659718	3.588816	4.332078
H	2.504246	4.265925	5.951337
C	0.828030	4.656618	4.630641
H	1.244373	5.591218	4.227488
H	0.171646	4.947825	5.468076
C	-0.014404	3.959644	3.564991
H	0.589678	3.750387	2.666131
H	-0.819399	4.633675	3.247450
Pd	0.486682	-0.855641	-1.078265
Al	-1.429872	-1.358777	0.443244
H	-0.745124	-2.217421	-0.936275
P	1.686305	-1.708138	-2.920953
N	-3.338455	-1.625913	0.048901
C	-4.190251	-2.210784	0.895183
C	-3.780418	-2.909237	2.039914
H	-4.573907	-3.294051	2.673420
C	-2.470712	-3.268852	2.400633
N	-1.390365	-2.807850	1.771766

C	-5.666854	-2.135465	0.622691
H	-5.947257	-2.711595	-0.267789
H	-6.244624	-2.517193	1.469253
H	-5.971689	-1.099796	0.423830
C	-2.304202	-4.234321	3.539965
H	-1.612365	-3.833884	4.292934
H	-3.261877	-4.450531	4.022110
H	-1.869158	-5.182530	3.199727
C	-3.817885	-1.239304	-1.243090
C	-4.031944	-2.242389	-2.208039
C	-4.488562	-1.865398	-3.473110
H	-4.673685	-2.638284	-4.219500
C	-4.697347	-0.527347	-3.791248
H	-5.047898	-0.250528	-4.784378
C	-4.466019	0.450989	-2.832249
H	-4.638910	1.501927	-3.065886
C	-4.032978	0.115279	-1.545957
C	-3.737342	-3.676839	-1.889991
H	-4.253046	-4.036471	-0.989518
H	-4.016231	-4.335332	-2.720197
H	-2.662541	-3.809528	-1.694218
C	-3.824869	1.169668	-0.509589
H	-2.778380	1.189266	-0.157686
H	-4.070423	2.165113	-0.897916
H	-4.439363	0.985607	0.385131
C	-0.130676	-3.451576	1.999524
C	0.827914	-2.879892	2.852869
C	2.047578	-3.543636	3.020808
H	2.790527	-3.104488	3.687071
C	2.312157	-4.742249	2.370763
H	3.266220	-5.246885	2.518283
C	1.356053	-5.289114	1.521237
H	1.562309	-6.218910	0.989160
C	0.133522	-4.651157	1.310400
C	0.555044	-1.606400	3.585258
H	1.443555	-1.264099	4.128760
H	0.249878	-0.798464	2.896140
H	-0.261946	-1.720530	4.315346
C	-0.866236	-5.205546	0.344089
H	-1.069558	-4.468802	-0.446406
H	-0.496683	-6.123023	-0.127902
H	-1.835961	-5.431454	0.810669
C	3.027237	-2.938167	-2.492949
H	3.844584	-2.291179	-2.131655
C	2.675800	-3.848913	-1.308449
H	1.857572	-4.534945	-1.575210
H	2.291707	-3.252301	-0.468672
C	3.894421	-4.667632	-0.892723
H	3.642007	-5.317424	-0.045016
H	4.682538	-3.983699	-0.533771
C	4.439194	-5.489379	-2.054747
H	3.678388	-6.225618	-2.363643
H	5.319502	-6.067706	-1.742751
C	4.779627	-4.596860	-3.240607
H	5.605630	-3.922351	-2.958773
H	5.144333	-5.194306	-4.087574
C	3.579860	-3.755352	-3.665881
H	3.865656	-3.095956	-4.496355
H	2.792159	-4.417631	-4.060453

C	2.563179	-0.525501	-4.122167
H	1.994721	0.412079	-3.992547
C	3.996329	-0.222663	-3.668407
H	4.610724	-1.134851	-3.743277
H	3.995483	0.071649	-2.605976
C	4.646647	0.860710	-4.522772
H	5.670551	1.053656	-4.170333
H	4.091021	1.800956	-4.391269
C	4.632978	0.489447	-5.997916
H	5.233393	-0.421288	-6.158858
H	5.099578	1.279800	-6.601330
C	3.202928	0.236360	-6.451393
H	2.621435	1.168518	-6.343811
H	3.167814	-0.028628	-7.516932
C	2.541453	-0.860121	-5.621090
H	1.514411	-1.008192	-5.976145
H	3.066856	-1.809926	-5.806745
C	0.575015	-2.641082	-4.098526
H	1.181857	-2.884895	-4.987395
C	-0.589935	-1.743486	-4.531467
H	-0.228142	-0.764489	-4.876243
H	-1.216368	-1.535656	-3.644670
C	-1.424912	-2.410917	-5.617540
H	-0.805422	-2.543921	-6.520942
H	-2.261972	-1.759249	-5.906747
C	-1.946323	-3.762946	-5.148901
H	-2.659718	-3.588816	-4.332078
H	-2.504246	-4.265925	-5.951337
C	-0.828030	-4.656618	-4.630641
H	-1.244373	-5.591218	-4.227488
H	-0.171646	-4.947825	-5.468076
C	0.014404	-3.959644	-3.564991
H	-0.589678	-3.750387	-2.666131
H	0.819399	-4.633675	-3.247450
